# *Gastrodia elata* in neurological disorders: a systematic review of anti-inflammatory mechanisms and cross-disease therapeutic potential

**DOI:** 10.3389/fnagi.2026.1967946

**Published:** 2026-10-26

**Authors:** Yi-yu Chu, Bo-yu Wang, Cai-yan Qu, Yu Lan, Li-ye Wang, Meng Mao

**Affiliations:** 1 Beijing University of Chinese Medicine, Beijing, China; 2 Guang’anmen Hospital, China Academy of Chinese Medical Sciences, Beijing, China; 3 Dongfang Hospital, Beijing University of Chinese Medicine, Beijing, China

**Keywords:** *Gastrodia elata*, gastrodin, neuroinflammation, neurological diseases, preclinical research

## Abstract

**Background:**

Neuroinflammation serves as a common pathological substrate across various neurological disorders, including neurodegenerative diseases, cerebrovascular diseases, and neuroimmune conditions. Key pathogenic mechanisms underlying these disorders include aberrant microglial activation, sustained release of pro-inflammatory cytokines, and dysregulation of critical signaling cascades, particularly the NLRP3 inflammasome, NF-κB, and Nrf2/HO-1 pathways, all of which collectively contribute to progressive neuronal damage and functional decline.

**Objectives:**

This review aims to systematically evaluate the preclinical evidence for the neuroprotective effects of *Gastrodia elata* Blume and its major bioactive constituents—gastrodin, parishin, p-hydroxybenzyl alcohol, and *G. elata* polysaccharides—in the treatment of Parkinson’s disease, Alzheimer’s disease, acute ischemic stroke, vascular dementia, and epilepsy, with a particular focus on their modulation of inflammation-related signaling pathways.

**Methods:**

A comprehensive literature search was performed in PubMed, Web of Science, ScienceDirect, China National Knowledge Infrastructure (CNKI), and Wanfang databases from inception to May 2026. Original studies investigating *G. elata* or its components in animal or cellular models of the aforementioned diseases and reporting inflammatory mechanisms were included.

**Results:**

*G. elata* and its active constituents regulate multiple neuroinflammation-associated signaling pathways, including Nrf2/HO-1, NF-κB/NLRP3, JAK2/STAT3, and MAPK, thereby suppressing inflammatory responses. Mechanistically, these effects are primarily manifested as: (1) inhibition of microglial M1 polarization and consequent reduction of pro-inflammatory cytokine release; (2) blockade of inflammation-induced neuronal death through suppression of ferroptosis, pyroptosis, and apoptosis; (3) enhancement of Nrf2 nuclear translocation, leading to upregulation of antioxidant enzymes and attenuation of oxidative stress-mediated inflammation (e.g., ROS-driven cascades); and (4) modulation of the gut microbiota–brain axis to reduce both peripheral and central low-grade chronic inflammation.

**Conclusion:**

Despite certain limitations in the existing evidence, this review clearly identifies future research priorities, including investigation of non-gastrodin active components, causal validation using gene-knockout models, and development of brain-targeted delivery strategies. These findings provide a valuable reference for the mechanism-guided development of neuroprotective agents derived from *G. elata*.

## Introduction

Neurological diseases such as Parkinson’s disease (PD), Alzheimer’s disease (AD), acute ischemic stroke (AIS), vascular dementia (VaD), and epilepsy rank among the major contributors to global disability and death (GBD 2019 Meningitis Antimicrobial Resistance Collaborators, 2023). Although these conditions differ substantially in their causes and pathological characteristics, a growing body of evidence has converged on neuroinflammation as a critical pathological link that underlies the progression of each of these disorders (Glass et al., 2010; Ransohoff, 2016).

Neuroinflammation is fundamentally an immune response of the central nervous system (CNS) to various endogenous or exogenous stimuli. However, when the inflammatory response becomes persistently dysregulated, it transitions into a pathological malignant cascade (Shi and Yong, 2025). In this process, excessive microglial activation, NLRP3 inflammasome assembly, sustained activation of the NF-κB signaling pathway, and overproduction of pro-inflammatory cytokines (e.g., IL-1β, TNF-α, IL-6) not only directly inflict neuronal damage but also exacerbate oxidative stress, disrupt blood–brain barrier (BBB) integrity, and impair neurogenesis, ultimately leading to cognitive and motor dysfunction (Heneka et al., 2018; Iadecola and Anrather, 2011; Ising et al., 2019).

With the growing body of research on natural products for the treatment of neurological diseases, their multi-component, multi-target characteristics are gradually overcoming the clinical bottleneck posed by the difficulty of addressing complex inflammatory networks through single-target interventions, thereby opening new avenues for the development of novel anti-inflammatory drug candidates (Shi Z. et al., 2024). *Gastrodia elata* Blume (*G. elata*), the dried tuber of an orchidaceous plant, is a well-known traditional Chinese medicine first recorded in Shennong’s Classic of Materia Medica as a top-grade herb, with the functions of “calming wind to relieve spasm, suppressing liver yang, and dispelling wind to unblock meridians.” Modern pharmacological studies have confirmed that *G. elata* and its principal bioactive constituents—including gastrodin, parishin, 4-hydroxybenzyl alcohol (4-HBA), and *G. elata* polysaccharide (GEP)—possess significant anti-inflammatory, antioxidant, anti-apoptotic, and neuroprotective activities (Liu et al., 2018a; Tang et al., 2015). Notably, *G. elata* and its active components can simultaneously modulate multiple signaling pathways, such as Nrf2/HO-1, NF-κB/NLRP3, JAK2/STAT3, and MAPK. By regulating microglial polarization, inhibiting ferroptosis and pyroptosis, promoting PINK1/Parkin-mediated mitophagy, upregulating the expression of antioxidant enzymes, and reshaping the gut microbiota–brain axis, they exert broad neuroprotective effects in animal models of various neurological diseases, including PD, AD, acute ischemic stroke, VaD, and epilepsy (Shao et al., 2025; Wang et al., 2007; Zhang et al., 2022). The five neurological disorders addressed in this review, together with the inflammatory pathways they share, are illustrated in Figure 1.

**FIGURE 1:**
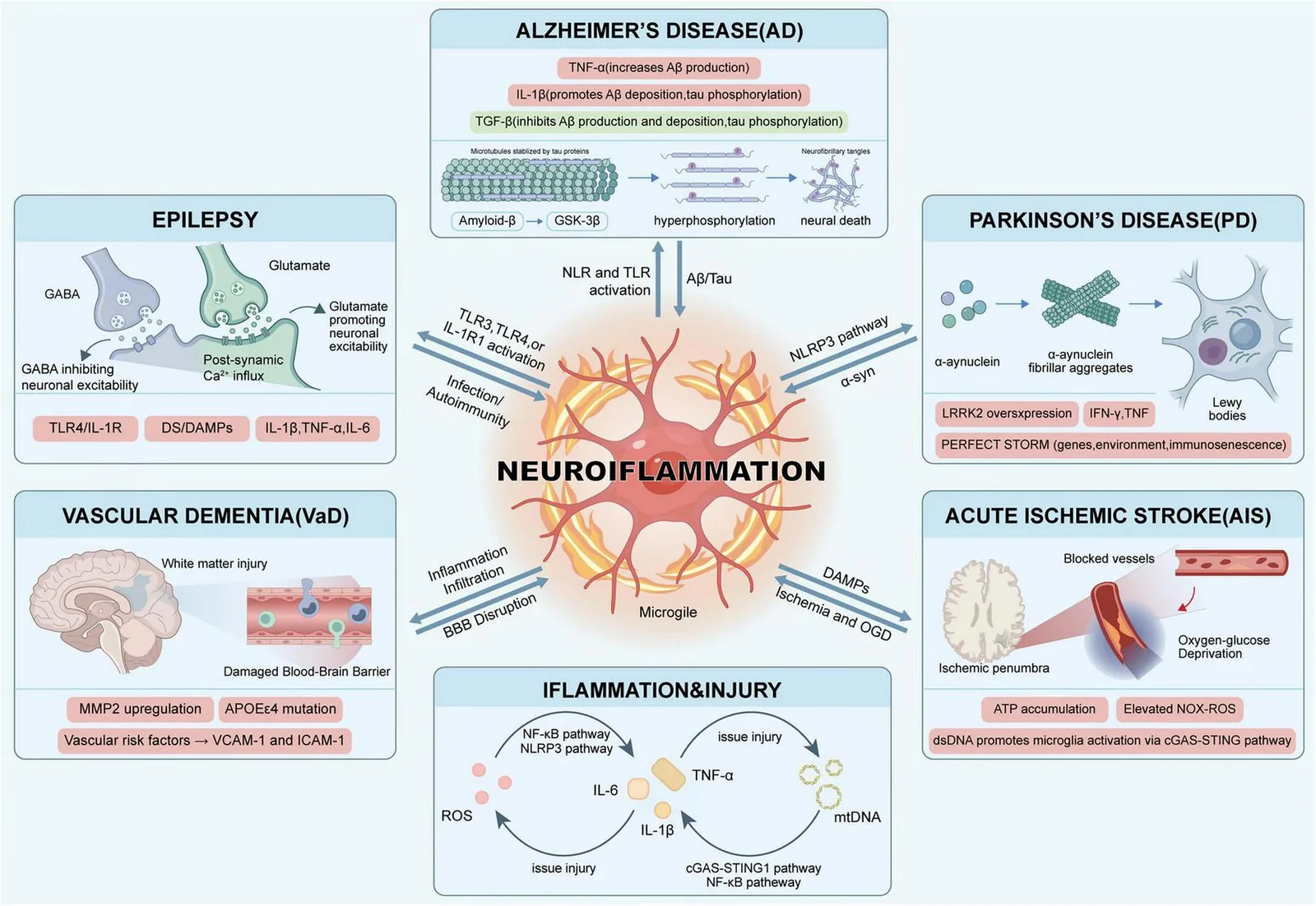
Possible pathogenesis of inflammation-induced neurological disease.

Despite the considerable number of preclinical investigations into the effects of *G. elata* and its bioactive constituents on the five neurological disorders addressed herein, a comprehensive review that systematically consolidates their mechanisms of action across these diseases from an inflammation-centric perspective has remained elusive. Over recent years, several review articles have summarized the neuropharmacological profiles of *G. elata* and its active components, the pharmacological actions of gastrodin (Dai et al., 2024), and its therapeutic promise in central nervous system disorders (Wang D. et al., 2025; Xiao et al., 2023). Additional contributions have focused on the neuropharmacological potential of *G. elata* and its constituents (Jang et al., 2015), the progress of gastrodin in the treatment of neurological diseases (Guo et al., 2025), the role of *G. elata* as a neuroprotective agent (Udi and Omuruka, 2025), and the pleiotropic effects of gastrodin glycosides (Mastrostefano et al., 2025). Nevertheless, the majority of existing studies are confined to a single disease entity or a solitary component, and a comparative synthesis of the shared and distinctive mechanisms across different disease models remains insufficient.Moreover, although the aforementioned reviews have furnished valuable insights into the neuroprotective actions of *G. elata*, none has systematically interrogated its anti-inflammatory mechanisms from a cross-disease comparative standpoint, nor have they integrated pharmacokinetic data, multi-pathway regulatory networks, and the gut microbiota–brain axis into a unified framework encompassing neurodegenerative, cerebrovascular, and neuroimmune disorders.

In this context, we systematically searched PubMed, Web of Science, ScienceDirect, CNKI, and Wanfang databases for original studies investigating *G. elata* or its active constituents in animal and cellular models of neuroinflammation, and subsequently synthesized the preclinical evidence pertaining to its therapeutic effects in Parkinson’s disease, Alzheimer’s disease, acute ischemic stroke, vascular dementia, and epilepsy. Our integrative analysis reveals that *G. elata* establishes a systematic, closed-loop neuroprotective network via a multi-level “microbiota–metabolism–epigenetics” regulatory axis, with mitochondrial homeostasis serving as the central hub, thereby synergistically integrating anti-inflammatory and antioxidant actions and ultimately abrogating cell death pathways. This review offers three distinctive contributions that distinguish it from prior works. First, from a mechanistic perspective, to the best of our knowledge, it is the first to systematically compare inflammatory signatures across five neurologically distinct disease categories within a unified analytical framework. Second, from a dimensional standpoint, it proposes the “microbiota–metabolism–epigenetics” regulatory axis as an integrative paradigm, elucidating how *G. elata* orchestrates both peripheral and central anti-inflammatory effects. Third, from a translational viewpoint, it critically evaluates pharmacokinetic bottlenecks—including the high aqueous solubility and low blood–brain barrier permeability of gastrodin, as well as disease state–dependent alterations in absorption—discusses the paucity of clinical evidence for non-gastrodin components, and proposes actionable strategies, such as brain-targeted delivery systems, intranasal administration, and conditional gene-knockout validation, to expedite clinical translation. Finally, we discuss the inherent limitations of the current evidence base and outline future directions for mechanistic refinement and formulation innovation, with the aim of informing the continued development and clinical application of *G. elata*.

## Materials and methods

### Search strategy and selection criteria

Databases: PubMed, Web of Science, ScienceDirect, China National Knowledge Infrastructure (CNKI), and Wanfang Database were systematically searched.

Keywords: The following search terms were used: *G. elata*, gastrodin, parishin, neuroinflammation, Parkinson’s disease, Alzheimer’s disease, cerebral ischemia, vascular dementia, epilepsy, and animal model. Boolean operators (AND/OR) were applied as appropriate.

The search strategies, PRISMA checklist, and source data for Tables 1, 2 and Figure 4 are provided in the Supplementary material.

**TABLE 1:** Summary of mechanisms by which *Gastrodia elata* and its active components intervene in neurological diseases.

Drug	Disease	Animal species/strain	Model	Intervention	Dose	Dosing regimen	Results	References
Water extracts of GE (WGE) and its bioactive compounds, gastrodin and 4-HBA	PD	LexAop-LRRK2-WT and LexAop-LRRK2-G2019S	Fly model of PD carrying the LRRK2-G2019S mutation	p.o.	Different concentrations (0.1, 0.5, or 1.0%, w/w)	3 months	G2019S hyperphosphorylation↓Lrrk2 protein↓ Akt/GSK3β/Nrf2 signaling axis↑ Nrf2↑Mad↓GFP↑	Lin et al., 2021
		Transgenic LRRK2-G2019S mice	LRRK2-G2019S transgenic mice model	–	0.5 g/kg body weight per day	3 months	pSmad3/Smad3↓	
*G. elata* polysaccharide/GEP-medicated serum (GMS)	PD	8-week-old male C57BL/6 mice/SH-SY5Y neuroblastoma cells	MPTP-induced PD mice	Oral administration	100, 200 mg/kg/day	2 weeks	Tyrosine hydroxylase expression↑ Microglial activation↓NLRP3↓IL-1β, IL-6, TNF-α↓ ROS↓ mitochondrial membrane potential↑ Autophagy in SH-SY5Y cells↑	Li S. et al., 2025
GEP	PD	Male C57BL/6 mice	MPTP-induced PD mice	Oral administration	100, 201 mg/kg/day	3 weeks	Bcl-2/Bax ratio↑ Cleaved-caspase-3↓Mitochondrial apoptosis↓ GFAP↓TLR4/NF-κB↓TNF-α,IL-1β,and IL-6↓	Gan et al., 2024
Gastrodin	PD	Obtained from Caenorhabditis Genetics Center	The *C. elegans*	–	Different concentraions (25, 50, 100, 200 μM)	24 h	DAF-2↓DAF-16↑ α-synuclein protein↓	Yan et al., 2019
Gastrodin	Neuroinflammation	Three-month old male SD rats	Lipopolysaccharide (5 mg/kg) induced model of neuroinflammation	Intraperitoneal injection	25 mg/kg	3, 7, 14 days	TLR4↓NF-κB (P65)↓ NLRP3↓ASC↓Caspase-1↓IL-1β↓IL-18↓	Zheng et al., 2022
Gastrodin	PD	Drosophila	–	In their standard diet	0, 0.08, 0.4, and 2 mM	–	SOD↑CAT↑ Delays the onset of Parkinson-like phenotypes in Pink1B9	He et al., 2021
Gastrodin	PD	C57BL/6 mice (8 weeks old, male)/PC12 (MD04) cell	MPTP/MPP+-induced PD mice	Oral	30 mg/kg	15 daus	Bcl2/Bax ratio↑ VMAT2↑vesicle volume↑ ROS↓dysfunction of DAH↓ MEK/ERK/CREB↑VMAT2↑	Zhao et al., 2024
20C	PD	\PC12 cell SH-SY5Y cell	A53T α-syn transgenic mice	–	50 mg/kg 2.5 to 40 μM	24 h	A53T α-Syn aggregation *in vitro*↓ The formation of α-Syn aggregates↓ Protect mitochondria	Peng et al., 2023
HBA	PD	SH-SY5Y cell	6-OHDA-induced oxidative stress	–	40, 80, 120, 160 μmol/L	1 h	c-Jun N-terminal kinase↓Bcl-2/Bax ratio↑Apaf-1↓caspase-9, caspase-3, and PARP cleavage↓	Lai et al., 2022
GE fermented by *Lactobacillus plantarum*, *Acetobacter pasteurianus*, and *Saccharomyces* (FGE)	AD	Male SPF-grade KM mice	D-Gal/AlCl3-induced Alzheimer’s disease-like mice	Intragastric drug administration	Control, model, donepezil (1 mg/kg⋅bw), FGE-L (120 mg/kg⋅bw), FGE-M (240 mg/kg⋅bw), and FGE-H (720 mg/kg⋅bw) groups	7 weeks	Aβ↓Tau↓P-Tau↓AchE↓ MDA↓ Ach↑ChAT↑SOD↑	Wang et al., 2024
Ginger-processed and fermented *G. elata* BI. (FGGE)	AD	SPF-grade male Sprague–Dawley (SD) rats	AlCl3-induced Alzheimer’s disease rat	Gavage	L, and H groups were 0.32 and 0.72 g/kg/day	40 days	GSH↑CAT↑ CREB↑BDNF↑SYN↑PSD95↑ Firmicutes-to-Bacteroidota (F/B) ratio↑ The abundance of the Euryarchaeota phylum↑	Huang et al., 2025
Gastrodin	AD	C57BL/6 mice BV-2 cells	LPS-treated C57BL/6 mice	Intraperitoneal injection	100 mg/kg/day 1.3 mg/kg/day	5 days	TLR4/TRAF6/NF-κB↓ Microglia and astrocytes↓p-Stat3↓ TNF-α和 IL-1β mRNA↓Arg-1 mRNA↑	Wang et al., 2024
Gastrodin	AD	SH-SY5Y cells bEnd.3 cells	3xTg-AD transgenic mice	–	未获取原文	–	ADRA1↑ADRA1/NF-κB/NLRP3↓ IL-1β and IL-18 release↓ Tight junction protein↑	Li B. et al., 2025
Gastrodin	AD	–	3xTg-AD mice	Intragastric administration	low (50 mg/kg) and high (100 mg/kg)	30 days	IL-6, TNF-↓MMP8↓ FERMT2↓ ECM remodeling↑integrity of the BBB↑	Wang L. et al., 2025
Gastrodin	AD	C57BL/6-SJL mice SH-SY5Y cells	Tg2576-AD mice	Gavage	60 mg/kg daily	3 weeks	PKR/eIF2α↓BACE1↓ SOD and CAT activities↑MDA↓	Zhang et al., 2016
EEGE included phosphorylated (p)-hydroxybenzyl alcohol, p-hydroxybenzaldehyde and 4,4’-dihydroxydi phenylmethane	AD	–	*Caenorhabditis elegan*s	–	0.25, 0.5, 1, and 2 mg/ml	–	SOD↑CAT↑MDA↓ROS↓ Aβ plaque aggregation↓insulin/IGF-1 signaling (IIS):hsp-12.3 and hsp-12.6 (增强抗应激能力)↑	Shi et al., 2023
PHBA	AD	–	*Caenorhabditis elega*ns model of Alzheimer’s disease	–	0.125, 0.25, 0.5, 1, 2, and 4 mM	–	Nuclear localization of DAF-16↑ SKN-1↑SOD−3↑GST−4↑ ROS↓Aβ aggregation↓	Yu et al., 2024a
4,4’-methylenediphenol	AD	\	*Caenorhabditis elegan*s model of Alzheimer’s disease	–	0.125, 0.25, 0.5, 1, 2, and 4 mM	–	Nuclear localization of DAF-16↑ SKN-1↑SOD−3↑GST−4↑ ROS↓Aβ aggregation↓	Yu et al., 2024b
HBA	AD	ICR mice/SH-SY5Y cell	Aβ42-induced Alzheimer’s disease mouse model	Administered intragastrically	5, 15 mg/kg 20, 40, 80, 120,and 160 μM	Once a day for 18 days	TNF-α↓IL-1β↓ BDNF&GDNF mRNA↓protein↓ Nrf2↑	Ding et al., 2019
GPP	AD	–	H_2_O_2_ induced PC12 cell	–	50, 100, and 200 μg/ml	–	SOD, CAT, and GSH-Px↑MDA↓ROS↓ NO, IL-6 和 TNF-α↓ Caspase9↓	Tan et al., 2022
miRNA	AD	Male C57BL/6 mice SH-SY5Y cells	D-Gal/AlCl3-induced Alzheimer’s disease-like mice	Administered intragastrically	Low (3 mg/kg), medium (6 mg/kg), and high (9 mg/kg)	40 days	GSK-3β↓hyperphosphorylation of Tau↓ Gas-miR-36-5p regulates GSK-3β expression by directly binding to the 3′-untranslated regions	Lu et al., 2023
miRNA	AD	Female ICR mice	ICR mice	Gavage	Total RNAs of fresh *G. elata* (30 μg/kg), *Gastrodia* decoction (3 mg/kg) and *Gastrodia* powder (3 mg/kg)	Per 2 h with six times during 12 h	Protein A20↓	Xia et al., 2020
Gastrodin	AD	Human neuroblastoma SH-SY5Y cells	Okadaic acid (OA) induced AD model	–	–	–	T-AOC and SOD↑MDA↓Bax↓Bcl↑ p-AKT (Ser473)↑p-GSK-3β (Ser9)↑ p-Tau (Ser396)↓p-Tau (Thr181)↓	Li et al., 2026
TM extract	IS	Male Sprague–Dawley rats	1% dimethyl sulfoxide induced CI rats	Intraperitoneally administered	0.25 g/kg (DO + TM-0.25 g), 0.5 g/kg (DO + TM-0.5 g), or 1 g/kg (DO + TM-1 g)	After cerebral ischemia onset	(p-JNK)/JNK↓TRAF3↓T3JAM↓p-NF-κB p65)/NF-κB p65↓ (Iba1)↓CD86↓TNF-α↓(IL)-1β↓IL-6↓ Iba1 coexpression (TLR4\CD86p-NF-κB p65)↓ IL-10, nerve growth factor and vascular endothelial growth factor A↑	Huang et al., 2024
Protein components in rhizoma gastrodiae (GEPS)	IS	Male C57BL/6 mice HT22 cells	MCAO induced focal cerebral ischemia mouse	Intraperitoneally administered	\	7 days before treatment	ERK↓PI3K/Akt↑ Blc-2↑Bax\caspase-3↓ MDA↓SOD↑GSH−px↑ BDNF↑GAP43↑	Duan et al., 2023
*G. elata* ethanol extract (GEE)	IS	Male Sprague–Dawley rats SH-SY5Y cells	Middle cerebral artery occlusion/reperfusion (MCAO/R) model	Gavage	40, 80, 160 mg/kg	7 days before surgery	pAkt↑Nrf2↑HO-1 and NQO1 mRNA↑SOD\GSH↑ DHE↓MDA↓H2O2↓ GPX4↑COX2↓(铁凋亡抑制)	Wang et al., 2026
Gastrodin	IS	Male Sprague–Dawley rats	Focal cerebral I/R surgery model	Gavage	Sham, I/R, I/R+15 mg/kg GAS, I/R + 30 mg/kg GAS, I/R +60 mg/kg GAS, and I/R +12 mg/kg Nim	7 days before/after I/R surgery	IL-1β, COX-2 and iNOS↓ Caspase3↓	Liu et al., 2016
Gastrodin	IS	The male Sprague Dawley rats	MCAO induced focal cerebral ischemia mouse	Intraperitoneal injections	100 mg/kg body weight	10 days (3 days before surgery and an additional 7 days post-surgery)	FOXO3a, SOD1, and SOD2↑ TNF-α↓IL-1β↓IL-6↓inflammatory factors and TLR9 expression↓ p-JAK2↓p-STAT3↓	Zhang et al., 2024a
Gastrodin	IS	Male Sprague–Dawley rats/SH-SY5Y cells	HIBD model/OGD/R model	Injected intraperitoneally	50, 100, 200 mg/kg/ Gastrodin injection	Immediately after modeling	TNF-α↓IL-6↓TGF-β↑ SOD, GSH-PX, CAT↑MDA↓ Nrf2↑HO−1↑P62↑Keap1↓	Wu et al., 2025
Gastrodin	IS	Adult male C57BL/6J mice/cardiac microvascular endothelial cells	Myocardial I/R animal model	Intraperitoneally administration	100 mg/kg	Once a day, for 3 days	NLRP3/caspase-1 signaling pathway↓IL-1β↓	Sun et al., 2019
Neutral polysaccharide from *Gastrodia elata*	IS	10-week-old male C57BL/6 J mice	MCAO/R model	–	20 mg/mL	–	ROS, Fe^2+^, MDA↓ GSH, SOD↑GPX4↑ NRF2, HO-1↑	Zhang et al., 2024b
Parishin C	IS	Male Wistar rats (8–10 weeks old and weighing 200–220 g)	MCAO/R model	Intraperitoneal injection	25 mg/kg/day	For 3 weeks prior to MCAO	CAT, GSH-Px, SOD↑MDA↓ TNF-α减少对小胶质细胞的激活IL-6, IL-1β↓	Wang et al., 2021
The phenolic components of *G. elata*	IS	Adult male Sprague-Dawley rats/Primary human astrocytes (Sciencell, HA-1800)	MCAO/R model	Intragastric administration	4 mg/kg	For 7 days before MCAO	Nrf2↑HO-1, NQO-1, BDNF↑	Shi et al., 2018
3,4-dihydroxybenzaldehyde	IS	Male Sprague–Dawley rats	Middle cerebral artery occlusion/reperfusion (MCAO/R) model	Gavage	10 mg/kg	7 days	Mitochondrial respiratory chain enzyme complexes I–IV↑mPTP↑MMP mitochondrial Cyt-c content↑	Luo et al., 2023
4-hydroxybenzaldehyde	IS	Healthy male Sprague-Dawley rats	Rat model of cerebral ischemia-reperfusion injury/cellular oxidative stress response model	4-hydroxyben zaldehyde	10 and 20 mg/kg	–	Malondialdehyde and 4-hydroxy-2-nonenal↓ Capture O_2_⋅− and ⋅ OH *in vitro*	Feng et al., 2025a
4-Hydroxybenzyl Alcohol	IS	Male Sprague–Dawley rats	MCAO/R model	Intraperitoneally administration	–	At 1 h prior to or 1, 6 h post-MCAO	Nrf2↑HO−1↑GCLM↑ ROS↓PARP-1↓Zn2+-induced cell death↓ p67↓PAR↓ (in astrocytes)	Luo et al., 2018b
Gastrodin	IS	Male C57BL/6 mice and BV2 cells	Middle cerebral artery occlusion (MCAO) model/oxygen glucose deprivation (OGD)-treated	Intraperitoneally injection	100 mg/kg	3 consecutive days (10, 34, and 58 h of reperfusion)	miR-21–5p↑TXNIP↓NLRP3 inflammasome↓	Zhang et al., 2019
*G. elata* ethyl acetate extract(EGEE)	VaD	Male Sprague-Dawley rats and HT-22 cells	Two-vessel occlusion (2-VO) model/oxygen glucose deprivation (OGD)-treated	Intragastric administration	high-dose EEGE (102 mg/kg), and low-dose EEGE (34 mg/kg)	28 days	p-AMPK/AMPK, SIRT1, TREM2, IGF1↑ p-FoxO1/FoxO1 and p-NF-κB/NF-κB↓ TNF, IL-1β↓	Tao et al., 2026
Gastrodin	VaD	Male adult Sprague–Dawley rats\TNA2 astrocytes	MCAO reperfusion model/oxygen glucose deprivation (OGD)-treated	Intraperitoneal injection	–	6 h prior to MCAO	Caspase-1 and IL-18↓ p-STAT3, NLRP3, and NLRC4↓ STAT3 pathway and NF-κB activation in TNA2 astrocytes↓	Sui et al., 2019
Gastrodin	VaD	3-day-old Sprague–Dawley (SD) rats	Experimental HIBD model	Intraperitoneal injections	100 mg/kg	1 h before ligation of carotid artery, immediately, and 12 h after hypoxia	TNF-α↓ Notch-1, NICD RBP-JK and Hes-1↓ Sirt3↑NIT and Hes-1↓ 保	Guo et al., 2021
Gastrodin	VaD	Seventy-eight one-day-old Sprague-Dawley (SD) rats	Experimental HIBD model	Intraperitoneal injections	100 mg/kg	1 h before ligation of carotid artery, immediately, and 13 h after hypoxia	ACE, AT1, NOX-2, iNOS, and TNF-α↓	Liu et al., 2018b
Gastrodin	VaD	Male Sprague–Dawley rats/HT22 cells	Bilateral common carotid artery occlusion (BCCAO)/hypoxia model	Intragastric administration	GAS low-dose (25 mg/kg) GAS high-dose (50 mg/kg)	8 weeks	SIRT3↑TRAM↑dysfunction-mediated oxidative stress and senescence↓	Chen et al., 2024
Gastrodin	VaD	–	Two-vessel occlusion (2-VO) model	Intragastric administration	22.5 or 90 mg/kg/day	4 weeks	p-Tau↓Aβ1-40 and Aβ1-42 plaques↓	Shi et al., 2020
Gastrodin	VaD	Adult male Sprague-Dawley rats	Bilateral common carotid artery occlusion (BCCAO) model	Oral	15, 30, and 60 mg/kg	28 consecutive days	Aβ1-40/42, APP, and β-site APP-cleaving enzyme 1↓Aβ-related protein, a disintegrin and metalloprotease 10↑ Beclin-1, LC3-II, and p62↓Bcl↑Bax↓	Liu et al., 2018c
HBA, gas	VaD	Adult male Sprague–Dawley (SD) rats	MCAO Reperfusion Model	Intraperitoneal injection	25 and 50 mg/kg	4 weeks	Aβ1-42, P-Tau396, and P-Tau217 proteins↓	Wu et al., 2023
Gastrodin	VaD	6–8 week old male Wistar rats/SH-SY5Y cells	Two-vessel occlusion (2-VO) model/induced by hydrogen peroxide	–	–	–	MDA↓NFE2L2, ADH7, GPX2 and GPX3↑ Forkhead box O (FoxO) family and NFE2L2↑ROS↓	Li and Zhang, 2015
Gastrodin	VaD	Male Sprague-Dawley (SD) rats	Desflurane anesthesia model	Gavage	0.5 g/kg	1 h before desflurane anesthesia	IL-1β↓ p38↓	Wang et al., 2018
Gastrodin	PD	Human neuroblastoma SH-SY5Y cells	1-methyl-4-phenylpyridinium (MPP(+))-induced	–	1, 5, 25 μM	4 h prior to exposure to	p38↑HO−1↑Nrf2 nuclear translocation↑	Jiang et al., 2014
Gastrodin	–	HT-22 cells	Exposed to 5 mM glutamate for 24 h	–	1, 5, 25 μM	24 h	Nrf2↑HO−1↑GPX4↑ PTGS2↓	Jiang et al., 2020
Gastrodin	VaD	Male Sprague–Dawley rats/HT22 cells	Bilateral common carotid artery occlusion (BCCAO) model	Combined with ferroptosis agonist	GAS low-dose group (25 mg/kg), GAS high-dose group (50 mg/kg)/25, 50 and 100 μmol/L	8 weeks	Nrf2↑GPX4↑COX2↓ Fe^2+^↓ROS,MDA↓GSH↑	Li et al., 2022
HBA	VaD	HT22 cells	OGD/R model	–	0, 12.5, 25.0, 50.0 or 100.0 μM	24 h before or without OGD/R injury	Choline-activated phospholipase D2/sirtuin 1/peroxisome proliferator-activated receptor-γ coactivator 1α pathway护线粒体	Xiao et al., 2025
Gastrodin	VaD	Adult male Sprague-Dawley rats/BV-2 microglia	MCAO reperfusion model OGD/R	Intraperitoneal injection	50, 100, and 200 mg/kg 20, 40, 80 μM	12 h	MMP2 and MMP9↓ BBB impairment↓ and inflammation↓	Li et al., 2019
4-HBd	VaD	Adult male Sprague–Dawley rats/Murine brain endothelial (bEnd.3) cells	MCAO/R model/hydrogen peroxide (H_2_O_2_) model	Intragastric administration	10 or 20 mg/kg 3.125–50 μg/mL	7 days 24 h	MDA,ROS↓NO↓iNOS↓SOD↑ MMP-2 and MMP-9↓occludin and claudin-5↑AQP-4↓	Feng et al., 2025b
Gastrodin	AD	Male Kunming mice	ICV injection of Aβ1-42	Intraperitoneal (i.p.) injection	100 mg/kg	15 days (from day 3 to day 17)	AQP4↑Aβ↓tau,P-tau↓ BDNF, SYN, and PSD-95↑	Luo et al., 2022
Gastrodiae rhizoma	Seizure	Adult male C57BL/6 mice, aged between 8 and 10 weeks	A pilocarpine-induced status epilepticus (SE) model	Intragastric administration	883.56 mg/kg	14 days as pretreatment	p-mTOR↓SEMA3F↑ Mitigate astrogliosis↓	Yip et al., 2020
Gastrodin	Seizure	Healthy adult male C57BL/6 mice	PTZ Model	Intraperitoneal (i.p.) injection	50, 100, 200 mg/kg	24 h after injection	IL-1β,TNF-α↓IL-10↑ CD11b-positive microglia↑MKP-1 phosphorylation↑MAPK signal pathway↓	Chen et al., 2017
Gastrodin	Seizure	Male SD rats	PTZ-induced epilepsy rat model	Intraperitoneal (i.p.) injection	15, 30, or 60 mg/kg	Every day	AchE↓IL-1β, TNF-α and IL-6↓ SOD, CAT, and GSH-PX↑Nrf2↑ NOX2↓	Zhou et al., 2025
Parishin B	Seizure	7 dpf zebrafish larvae	Pentylenetetrazol (PTZ)-induced seizure model	–	0.0625, 0.125, or 0.25 mg/mL	60 min	Restore dopamine and norepinephrine levels il1b, tnfa, and nfkb1↑IL-6,SOD↑ ACLY↑	Sun et al., 2026
*G. elata* Bl	Seizure	Male C57BL/6J mice	Cocaine model	p.o.	500 or 1,000 mg/kg	1 h before cocaine injection	GABAA receptors↑	Shin et al., 2011
Gastrodin	Seizure	Adult male Sprague-Dawley rats	Lithium-Pilocarpine seizure model	i.p.	50 mg/kg,	20 min before pilocarpine injection	GABAA receptor α1↑	Yang et al., 2021
Trimethylcitryl-beta-D-galactopyranoside	Seizure	–	–	–	10 microg/ml	–	GABA transaminase activity↑	Choi and Lee, 2006
4-hydroxybenzaldehyde 4-hydroxy-3-methoxybenzaldehyde	Seizure	–	–	–	IC(50) = 4.1 and 5.4 microg/ml, respectively	\	GABA transaminase activity↑	Ha et al., 2001
Vanillin (VAN) and p-hydroxybenzaldehyde (p-HB)	Seizure	IMR32 human neuroblastoma cells	Glutamate-induced apoptosis	–	–	–	Intracellular Ca^2+^ rise and apoptosis↓	Lee et al., 1999
Methanol extract from *G. elata* (MEGE	Seizure	HT22 hippocampal cells	Glutamate-mediated oxidative stress	–	–	–	Phosphorylated p38↓dephosphorylated PI3K↓ROS↓BDNF↑	Han et al., 2014
GEAT decoction	Seizure	SPF adult male Kunming mice	MES, PTZ, and 3-MP injection induced acute seizures	po.	50, 100, 200 mg/kg	7 days	CAT and SOD activities↑GSH↑MDA↓ TNF-α, IL-1β, and IL-6↓	He et al., 2023
A series of peptide analogs 1–30 of 3,4,5-trimethoxycinnamic acid (TMCA)	Seizure	Male KunMing-strain mice	the maximal electroshock (MES) and the sc-pentylenetetrazole (PTZ) models	i.p.	75, 50, 25, 12.5 mg/kg	Before test	GABA aminotransferase (GABA-AT)/gamma-aminobutyric acid type A receptor (GABAAR)↑	Zhao et al., 2026
*Uncaria rhynchophylla* (MIQ) Jack and *G. elata* Bl.	Seizure	Male Sprague-Dawley (SD) rats	Kainic acid (KA) induced epileptic seizures model	i.p.	UR 1 g/kg alone or UR 1 g/kg plus GE 1 g/kg	–	Free radical scavenging activities↓	Hsieh et al., 1999
*G. elata* extract	Seizure	Sprague-Dawley rats	Pilocarpine-induced epileptic rat model	p.o.	3 and 10 g/kg	Daily for 7 days	VPA levels in the brain↑ DOPAC and 5-HT levels↓	Yang and Tsai, 2022

Type A: studies using *G. elata* alone or a well-defined single constituent (e.g., gastrodin, parishin, 4-HBA, GEP). Type B: studies using herbal combinations or co-interventions (e.g., GEAT decoction, *G. elata* with *Uncaria rhynchophylla*, gastrodin combined with valproic acid). For Type B studies, the observed effects cannot be exclusively attributed to *G. elata* or its constituents, and these findings are interpreted as supportive rather than direct evidence in the mechanistic synthesis.

**TABLE 2:** Overview of registered clinical studies on *Gastrodia*-related interventions: human use and translational readiness.

NCT-number	Study start	Disease	Interventions	Phase	Study design	Country
NCT04035824	2019/7/29	Hypertension	DRUG: *Gastrodia* and *Uncaria* granule; DRUG: Placebo of *Gastrodia* and *Uncaria* granule	4	Allocation: RANDOMIZED | Intervention Model: PARALLEL | Masking: DOUBLE (PARTICIPANT, CARE_PROVIDER) | Primary Purpose: TREATMENT	China
NCT06785623	2025/1/21	Depression; cancer patients and depression and suicide; life quality	DIETARY_SUPPLEMENT: *G. elata* Blume water extract	NA	Allocation: RANDOMIZED | Intervention model: PARALLEL | Masking: SINGLE (PARTICIPANT) | Primary purpose: SUPPORTIVE_CARE	Taiwan, Province of China
NCT02156024	2014/6/5	Masked hypertension	DRUG: *Gastrodia* and *Uncaria* Drink	4	Allocation: RANDOMIZED | Intervention model: PARALLEL | Masking: SINGLE (PARTICIPANT) | Primary purpose: TREATMENT	China
NCT07422142	2026/2/19	Delirium	DRUG: gastrodin injection; placebo	1	Allocation: RANDOMIZED | Intervention model: PARALLEL | Masking: QUADRUPLE (PARTICIPANT, CARE_PROVIDER, INVESTIGATOR, OUTCOMES_ASSESSOR) | Primary purpose: TREATMENT	China
NCT07283562	2025/12/16	Sensorineural Hearing Loss; Vertigo	DRUG: gastrodin Injection; DRUG: conventional therapy	NA	Allocation: RANDOMIZED | Intervention model: PARALLEL | Masking: SINGLE (OUTCOMES_ASSESSOR) | Primary purpose: TREATMENT	China
NCT00297245	2006/2/28	Cognitive decline	DRUG: gastrodin	4	Allocation: RANDOMIZED | Intervention model: PARALLEL | Masking: DOUBLE | Primary purpose: PREVENTION	China
NCT06012994	2023/8/28	Cardiac surgical procedures	DRUG: gastrodin	NA	Observational Model: COHORT | Time perspective: RETROSPECTIVE	China
NCT06012968	2023/8/28	Cardiac surgical procedures	DRUG: gastrodin	NA	Observational Model: COHORT | Time perspective: PROSPECTIVE	China
NCT03221894	2017/7/19	Alzheimer disease	DIETARY_SUPPLEMENT: GRAPE granules	NA	Observational model: COHORT | Time perspective: RETROSPECTIVE	China

The studies listed in this table provide contextual information regarding the safety, tolerability, and translational feasibility of *Gastrodia*-related interventions in human populations. However, they should not be interpreted as direct evidence of clinical efficacy for the five neurological disorders that are the primary focus of this review, as the majority of these trials address conditions such as hypertension, cardiac surgery-related delirium, and hearing disorders. This table is intended to complement the preclinical evidence by illustrating the current landscape of human investigation and to highlight areas where translational research is most advanced.

Inclusion criteria: Studies were included if they met the following criteria: (1) original research articles (including both *in vivo* animal studies and *in vitro* cellular experiments); (2) intervention with *G. elata* extract or its clearly defined active monomeric components (e.g., gastrodin, parishin, 4-hydroxybenzyl alcohol), without restrictions on dosage, route of administration, or treatment duration; (3) use of well-established animal models of neurological diseases or cellular models of inflammation; and (4) reporting of mechanistic outcome measures related to neuroinflammation (e.g., levels of inflammatory cytokines, expression of signaling pathway proteins, microglial polarization status, or cell death markers).

Exclusion criteria: Clinical studies, reviews, meta-analyses, conference abstracts, case reports, and articles not published in English or Chinese were excluded.

Selection procedure: The literature screening was performed independently by two reviewers (initials Yi-yu Chu and initials Bo-yu Wang). In the first stage, both reviewers screened the titles and abstracts of all retrieved records against the pre-defined inclusion and exclusion criteria. Subsequently, full-text articles of potentially eligible studies were independently assessed by the same two reviewers for final inclusion. Any disagreements that arose during either the title/abstract or full-text screening phases were resolved through discussion between the two reviewers; if consensus could not be reached, a third reviewer (initials Cai-yan Qu) was consulted to arbitrate and reach a final decision. The entire screening process was documented, and the reasons for exclusion at the full-text stage are presented in the PRISMA flow diagram.

Flowchart: The literature screening process was illustrated using a PRISMA 2020 flow diagram (Figure 2). The characteristics of the studies finally included, including the drug, disease, animal species/strain, model, intervention, dose, dosing regimen, results and corresponding references, are summarized in Table 1.

**FIGURE 2:**
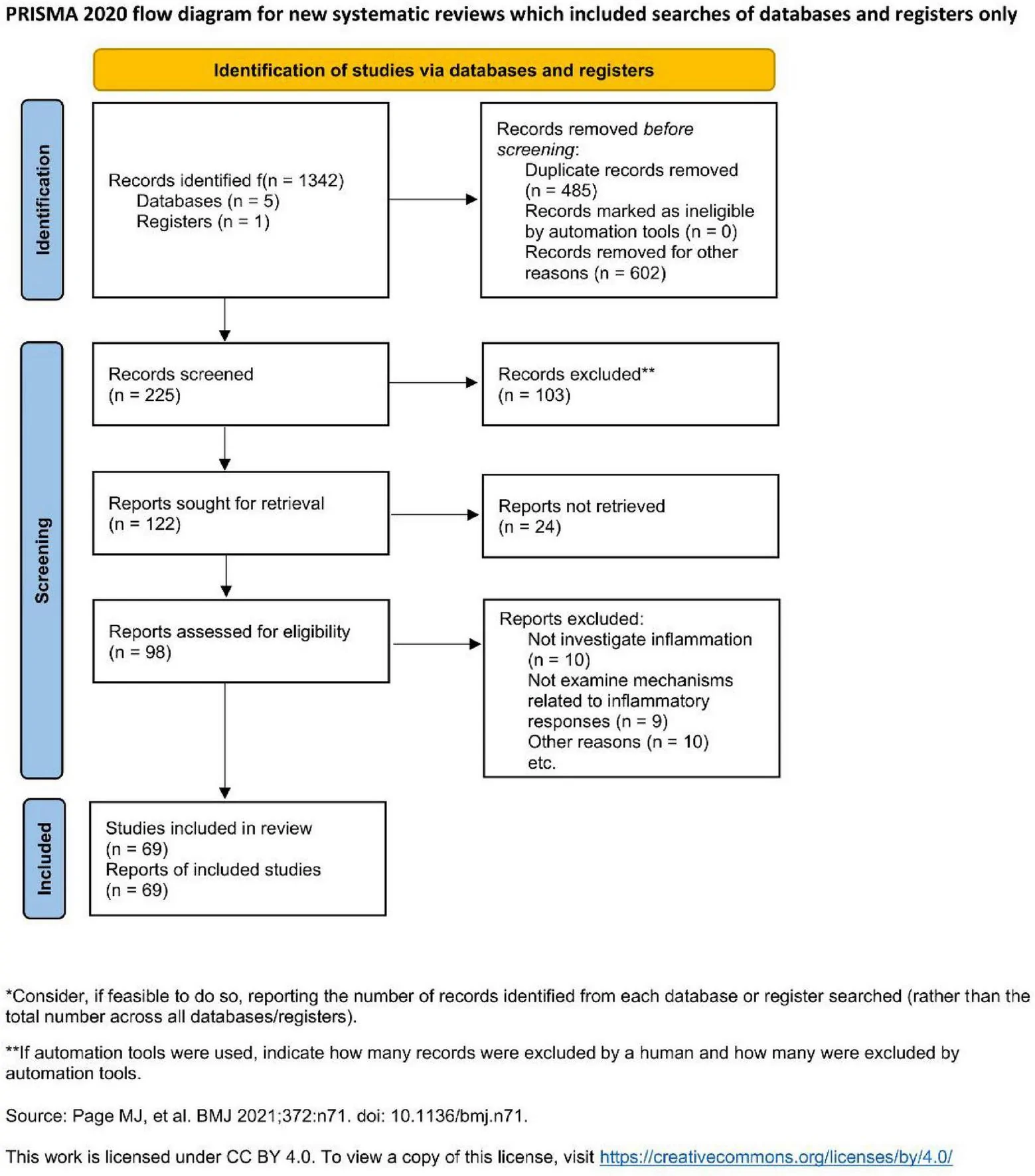
Search strategy and methods.

### Risk of bias assessment

The methodological quality of the included animal studies was assessed using SYRCLE’s risk of bias tool (Hooijmans et al., 2014), which was adapted from the Cochrane risk of bias tool for animal intervention studies. Only *in vivo* mammalian experiments were eligible for assessment. For studies containing both eligible mammalian experiments and ineligible *in vitro* or non-mammalian experiments, only the mammalian *in vivo* components were assessed.

The tool comprises ten items addressing six types of bias: sequence generation, baseline characteristics, and allocation concealment (selection bias); random housing and blinding of caregivers and/or investigators (performance bias); random outcome assessment and blinding of outcome assessors (detection bias); incomplete outcome data (attrition bias); selective outcome reporting (reporting bias); and other sources of bias. Other sources of bias included, where reported, ethical approval, funding and conflicts of interest, and major design or reporting inconsistencies.

Each item was judged as “low risk,” “high risk,” or “unclear risk” of bias according to the SYRCLE signaling questions. A judgment of low risk required explicit and adequate reporting of the relevant methodological safeguard. A judgment of unclear risk was assigned when the available information was insufficient to permit a definitive judgement; no assumptions were made about unreported procedures. A judgment of high risk was assigned when the publication explicitly described an inappropriate procedure or provided direct evidence of bias.

An overall risk of bias judgment was assigned to each study using prespecified rules: overall high risk if at least one item was judged as high risk; overall unclear risk if no item was high risk but three or more items were unclear; and overall low risk if no item was high risk and no more than two items were unclear. The overall judgment was used for descriptive purposes only and was not used as a threshold for study inclusion.

Two reviewers (Yi-yu Chu and Bo-yu Wang) independently assessed all included studies. Disagreements were resolved by discussion, with consultation of a third reviewer (Cai-yan Qu) when necessary. Inter-rater agreement was quantified using Cohen’s kappa statistic. Study-level results were visualized as a traffic-light plot and a summary bar plot using the robvis package (version 0.3.1) in R (version 4.6.1).

## Active components of *G. elata* and their pharmacokinetic characteristics

The pharmacological activity of *G. elata* is primarily attributable to its small-molecule constituents, among which gastrodin and parishin have been the most extensively investigated. Pharmacokinetic studies serve as a critical bridge linking the chemical composition of *G. elata* to its *in vivo* efficacy, facilitating the elucidation of its absorption, distribution, metabolism, and excretion (ADME) properties and providing a scientific basis for rational clinical use.

To date, research on gastrodin has covered the entire ADME process and has systematically revealed the *in vivo* metabolic transformation between gastrodin and parishin—specifically, parishin can be gradually hydrolyzed to generate gastrodin, thereby prolonging the duration of action of the active components. In recent years, the widespread adoption of highly sensitive analytical techniques such as LC-MS/MS has enabled increasingly robust simultaneous quantification of multiple active constituents in *G. elata*, offering important support for understanding the overall pharmacokinetic characteristics of its multi-component synergistic effects.

The following sections systematically review the ADME research progress of the active ingredients of *G. elata* from four perspectives: absorption, distribution, metabolism, and excretion.

## Absorption

### Absorption rate and bioavailability

Gastrodin is rapidly absorbed following oral administration. A ^3^H-labeling study demonstrated that radioactivity in rat blood increased markedly within 5 min after intragastric administration, peaked at approximately 50 min, and declined to only 1.1% remaining in the gastrointestinal tract by 8 h, indicating rapid and complete absorption (Lu et al., 1985). Lin et al. (2008) using microdialysis combined with LC-MS/MS, showed that gastrodin and its active metabolite p-hydroxybenzyl alcohol (HBA) quickly entered the bloodstream and distributed to brain tissue, providing direct pharmacokinetic evidence for its central effects.

The absolute oral bioavailability of gastrodin exhibits significant species differences. Huang et al. (2023) reported that oral bioavailability was higher in Wistar rats (84.7%) and Kunming mice (70.8%) than in Sprague-Dawley (SD) rats (60.6%) and C57 mice (49.9%). Because gastrodin is structurally highly similar to glucose and undergoes negligible passive diffusion across the intestinal epithelium, its oral absorption relies primarily on active transport mediated by intestinal glucose transporters (IGTs). Consequently, the expression pattern of these transporters in the gut directly determines absorption efficiency. Further investigation by Huang et al. (2023) revealed that the distribution profile of SGLT1/GLUT2 along the intestine (duodenum ≈ jejunum > ileum > colon) in SD rats and C57 mice most closely resembles that in humans, making these two strains preferred models for studying humanized intestinal glucose transporter-related pharmacokinetics.

### Influence of dosage form and compatibility on absorption

Dosage form and compatibility significantly influence the absorption kinetics of gastrodin. Tang et al. (2015) compared the pharmacokinetic profiles of rats following intragastric administration of free gastrodin, parishin, or *G. elata* extract. They found that the elimination half-life (t_1_/_2_) and mean residence time (MRT) of the latter two were markedly prolonged—parishin can be gradually metabolized to gastrodin *in vivo*, while the extract contains both free gastrodin and parishin conjugates, both of which continuously release gastrodin and thereby extend its duration of action (Tang et al., 2015). Further comprehensive pharmacokinetic modeling revealed that the oral bioavailability of parishin is approximately 14%. The overall pharmacokinetic characteristics of gastrodin injection and the extract were similar, whereas parishin and gastrodin capsules required multi-parameter evaluation (Tang et al., 2015). Additionally, SGLT1 promotes the intestinal absorption of gastrodin, whereas glucose and GLT inhibitors attenuate this process (Cai et al., 2013).

Disease states can also markedly alter the absorption of active ingredients from *G. elata*. Using UPLC-Q-TOF-MS and UPLC-QQQ-MS, Shao et al. (2025) compared the pharmacokinetic profiles of normal and cerebral ischemic rats after administration of *G. elata* extract. They observed that the gastrointestinal absorption of parishin A, B, C, E, and gastrodin was significantly reduced in the cerebral ischemia model rats, suggesting that the ischemic pathological state inhibits the intestinal uptake of *G. elata* components (Shao et al., 2025). In human pharmacokinetic studies, Ju et al. (2010) established an HPLC-PDA method for determining gastrodin concentrations in human plasma. After oral administration of 200 mg gastrodin capsules, the main pharmacokinetic parameters were as follows: Cmax 1484.55 ± 285.05 ng/mL, Tmax 0.81 ± 0.16 h, AUC_0_→∞5619.41 ± 1972.88 (ng/mL)⋅h, and AUC0→∞7210.26 ± 3472.74 (ng/mL)⋅h (Ju et al., 2010). These results are consistent with trends observed in animal experiments and further validate the oral absorption characteristics of gastrodin.

### Distribution

^3^H-labeling experiments revealed the tissue distribution profile of gastrodin and its metabolites. The highest radioactivity was observed in the kidneys, indicating that the drug or its breakdown products preferentially accumulate in this organ, which is consistent with the fact that gastrodin is primarily excreted via urine. Moderate radioactivity was detected in the liver, lungs, and uterus. Although overall radioactivity in the brain was low, its concentration did not peak until 2 h after administration, suggesting that gastrodin enters the brain slowly but can maintain a certain level of exposure (Lu et al., 1985).

The plasma protein binding rate of gastrodin is only 4.3%, whereas that of its active metabolite p-hydroxybenzyl alcohol (HBA) is as high as 69.3%. This difference may affect the tissue distribution and clearance rates of the two compounds (Lin et al., 2008). Brain tissue distribution is a core consideration in the evaluation of central nervous system (CNS) drugs. Shao et al. (2025) compared the brain-targeting characteristics of *G. elata* extract in normal and cerebral ischemic rats using UPLC-Q-TOF-MS and UPLC-QQQ-MS. A total of 53 components were identified, of which six were detectable in both plasma and brain tissue. Notably, although intestinal uptake of these components was impaired in cerebral ischemia model rats, their accumulation in brain tissue was markedly increased, a phenomenon primarily attributed to disruption of blood–brain barrier (BBB) integrity under ischemic conditions (Shao et al., 2025). To overcome the inherent bottlenecks of high water solubility and low BBB permeability of gastrodin, Wang et al. (2007) explored the intranasal administration route. Their results demonstrated that gastrodin can be directly transported into the brain via the olfactory pathway, achieving non-invasive brain-targeted delivery and providing a novel strategy for the treatment of CNS disorders (Wang et al., 2007).

### Metabolism

The primary metabolic pathway of gastrodin *in vivo* is hydrolysis to its aglycone, p-hydroxybenzyl alcohol (HBA). Using microdialysis coupled with LC-MS/MS, Lin et al. simultaneously detected gastrodin and HBA in rat blood, brain tissue, and bile, confirming that gastrodin is metabolized to HBA and that the latter can cross the blood–brain barrier and bind to benzodiazepine receptors, thereby mediating the central pharmacological effects of gastrodin (Lin et al., 2008). The gut microbiota plays a pivotal role in gastrodin metabolism. Nepal et al. (2019) anaerobically incubated gastrodin with the intestinal contents of normal rats and found that gastrodin was almost completely metabolized to HBA in a time- and concentration-dependent manner. In contrast, HBA production was markedly reduced when gastrodin was incubated with intestinal contents from antibiotic-pretreated rats. *In vivo* pharmacokinetic studies further demonstrated that antibiotic pretreatment significantly altered the metabolic conversion of gastrodin to HBA: although gastrodin was rapidly absorbed into the bloodstream and metabolized to HBA after oral administration, the total elimination time was approximately 6 h (Nepal et al., 2019). Beyond gastrodin, parishin also exhibits complex metabolic characteristics. Tang et al. (2015) reported that nine metabolites are generated following intravenous injection of parishin. By combining regression analysis with the area under the curve (AUC_0_-∞) and dose of gastrodin injection, the conversion rate of parishin to gastrodin was calculated to be 50%. Notably, the metabolic profiles of each parishin metabolite were similar across different doses, providing an important basis for understanding its multi-active metabolite network (Tang et al., 2015).

### Excretion

Gastrodin is primarily excreted in its unchanged form via the kidneys into the urine, with a minor fraction eliminated through the hepatobiliary pathway. It is almost undetectable in feces, and no enterohepatic circulation occurs in rats. A łH-labeling study showed that within 24 h after oral administration, the radioactivity excreted in urine, feces, and bile accounted for 66.1%, 0.63%, and 3.06% of the total administered dose, respectively, further confirming that urine is the predominant excretion route, whereas fecal and biliary excretion contribute minimally (Lin et al., 2007). Human pharmacokinetic studies revealed that following oral administration of 200 mg *G. elata* capsules, the distribution half-life (t_1/2_α) is 3.78 h, the elimination half-life (t_1/2_β) is 6.06 h, and the oral clearance (CL/F) is 62.50 L/h. These parameters are consistent with animal experiment results, indicating that gastrodin is rapidly distributed and moderately eliminated in the body (Ju et al., 2010).

## Preclinical evidence of *G. elata* treating specific neurological diseases through inflammatory mechanisms

### Parkinson’s disease

#### Inflammation in Parkinson’s disease

Parkinson’s disease (PD) is the second most common neurodegenerative disorder after Alzheimer’s disease. The global number of PD patients increased from approximately 3.15 million in 1990 to about 11.77 million in 2021, representing a 2.74-fold increase (Zhu et al., 2024). Inflammation can act as either a protective factor or a pathogenic risk factor in PD. As a risk factor, inflammation not only contributes to the non-motor symptoms of PD but has also become an important entry point for early diagnosis (Savica et al., 2009). Chronic immune stimulation leads to sustained, low-grade production of circulating inflammatory mediators and cytokines, which in turn promotes the onset and progression of PD (Lindqvist et al., 2012). Both innate and adaptive immunity are involved in this process. In innate immunity, α-synuclein (α-syn) interacts with inflammatory responses in a manner that varies with disease severity and α-syn species, generally exhibiting synergistic effects. Among the pathways linking α-syn to inflammation, the NLRP3 inflammasome pathway is the most prominent (Franceschi et al., 1999; Scheiblich et al., 2021). In adaptive immunity, the IFN-γ and TNF pathways have garnered increasing attention (Govindarajan et al., 2020). The “perfect storm” hypothesis attributes PD etiology to the interplay of genetic factors, environmental exposures, and immune senescence, with the first two being closely linked to age-related decline of the immune system (Gordon et al., 2019). Collectively, these findings indicate that inflammation is a critical contributing factor in the pathogenesis of PD.

#### Mechanisms of *G. elata* and its active ingredients in alleviating PD-related inflammation

Clinical studies have indicated that the classic prescription *G.elata*-*Uncaria rhynchophylla* decoction can serve as an adjunctive treatment for patients with Parkinson’s disease (PD), suggesting that *G. elata* has the potential to delay PD progression. The following sections systematically elucidate its anti-inflammatory mechanisms from the perspective of its active components. Multiple genetic variants that modulate the risk of specific diseases have been identified, some of which also regulate immune function, including LRRK2 (Zimprich et al., 2004), PRKN, and PINK1. *G. elata* water extract (WGE) has demonstrated efficacy in the LRRK2-G2019S mutation model. Lin et al. (2021) reported that WGE inhibited Lrrk2 protein overexpression and its G2019S phosphorylation by restoring Akt/GSK3β/Nrf2 pathway activity in astrocytes and sheath glial cells, while also antagonizing the BMP signaling pathway. Recent studies have shown that Lrrk2 participates in M1-type macrophage polarization by modulating cellular responses to cytokines and Toll-like receptors (e.g., IFN-γ and LPS). In the LRRK2-G2019S mouse model, WGE further suppressed Smad2/3 signaling, restored motor function, protected dopaminergic neurons, and attenuated excessive microglial activation.

The mechanisms of *G. elata* polysaccharides (GEP) involve mitochondrial quality control and inflammatory regulation. Recent evidence indicates a close relationship between mitophagy and inflammation. Mitochondrial components and metabolites—collectively termed mitochondrial damage-associated molecular patterns (mtDAMPs), such as mtDNA, mtRNA, ROS, ATP, and cytochrome c—are released into the cytoplasm due to altered mitochondrial permeability, thereby activating inflammatory responses via the cGAS–STING1 pathway and the inflammasome pathway (Marchi et al., 2023). GEP promoted mitophagy by activating the PINK1/Parkin pathway, reduced ROS levels, decreased NLRP3 inflammasome components, and downregulated the pro-inflammatory cytokines IL-1β, IL-6, and TNF-α. Another study demonstrated that GEP also reduced the production of MPTP-induced IL-1β, IL-6, and TNF-α in PD fruit flies by inhibiting the TLR4/NF-κB pathway (Shi and Yong, 2025). Furthermore, GEP upregulated the Bcl-2/Bax ratio, thereby reducing cleaved caspase-3 and inhibiting mitochondrial apoptosis (Gan et al., 2024).

Gastrodin, the most extensively studied active ingredient, exerts anti-inflammatory effects in PD models primarily through NLRP3 inflammasome inhibition and TLR4-NF-κB-NLRP3 axis regulation (Yao et al., 2020; Zheng et al., 2022).

Gastrodin significantly prolongs the lifespan of Pink1^*B*9^ mutant fruit flies, improves climbing ability, enhances resistance to oxidative stress, increases the activities of antioxidant enzymes (SOD, CAT), and upregulates the expression of antioxidant genes, thereby boosting the organism’s defense against oxidative stress (Zheng et al., 2022). In terms of neuroprotection, gastrodin protects dopaminergic neurons through the insulin-like DAF-2/DAF-16 pathway and the MEK/ERK/CREB pathway (Yan et al., 2019; Zhao et al., 2024). Activation of the MEK/ERK/CREB axis enhances VMAT2 expression, restores vesicular function, and maintains dopamine homeostasis. Concurrently, CREB nuclear translocation promotes BDNF expression, further supporting neuronal survival. Notably, dopamine homeostasis imbalance in the brain leads to extracellular dopamine accumulation, its conversion into endogenous neurotoxins, and exacerbation of dopaminergic neuronal injury through accelerated ROS and reactive metabolite generation (Hastings et al., 1996; Yao et al., 2022). Consequently, the protective effect of gastrodin in Parkinson’s disease largely depends on its activation of the MEK/ERK/CREB (MAPK) pathway.

Other components of *G. elata* also contribute to maintaining mitochondrial structural and functional integrity and preventing cell death. Compound 20C alleviates α-synuclein neurotoxicity by reducing its β-sheet content and regulates the expression of mitochondrial fission/fusion-related genes (Dnm1l, Mfn1, Mfn2, Mrps24) to preserve mitochondrial integrity (Peng et al., 2023; Xu et al., 1994). Another constituent, p-hydroxybenzyl alcohol (HBA), antagonizes 6-OHDA-induced mitochondrial cytochrome c release and caspase cascade activation, thereby exerting neuroprotective effects (Lai et al., 2022).

Importantly, the gut microbiota plays a significant role in the pathogenesis of PD and is closely linked to the initiation of inflammation (Lai et al., 2022). *G. elata* polysaccharide (GEP) modulates PD-related gut microbiota, including *Akkermansia*, *Lactobacillus*, *Bacteroides*, *Prevotella*, and *Faecalibacterium*, increases colonic short-chain fatty acid (SCFA) content, and upregulates the expression of the intestinal barrier protein occludin, thereby reducing both systemic and central inflammation (Gan et al., 2024).

In summary, *G. elata* exerts broad neuroprotective effects in PD models through multi-component, multi-target regulation of neuroinflammation, oxidative stress, and mitochondrial function.

### Alzheimer’s disease

#### Inflammation in Alzheimer’s disease

Alzheimer’s disease (AD) is the most common neurodegenerative disorder, accounting for approximately 60%–70% of global dementia cases. Clinically, it is characterized by progressive memory loss, cognitive dysfunction, and personality and behavioral changes. Amyloid-β (Aβ) plaques and neurofibrillary tangles (NFTs) formed by hyperphosphorylated tau protein are its core pathological hallmarks. Dysfunction of the meningeal lymphatic system reduces Aβ clearance efficiency, thereby exacerbating neuroinflammation (Nasb et al., 2024; Thakur et al., 2023). Microglia are activated in the early stages of AD, increase in number, and are closely associated with Aβ plaques, NFTs, and complement factors (Hickman et al., 2008). Activated microglia produce immune mediators such as TNF-α, IL-1, IL-6, and ROS through Toll-like receptor (TLR) and NOD-like receptor (NLR) pathways (Colton and Wilcock, 2010; Ha et al., 2012).

In AD-related neuroinflammation, IL-1 serves as a driving force for the spread of neuritic plaques within the cerebral cortex (Mrak and Griffin, 2007). In AD mouse models, overexpression of IL-1β promotes amyloid deposition, concurrently upregulates p38 MAPK and glycogen synthase kinase-3β (GSK-3β), and exacerbates tau phosphorylation (Ghosh et al., 2013). Ultimately, the number of plaque-associated microglia increases four- to six-fold, and microglial activation at inflammatory sites becomes more robust.TNF-α, through activation of NF-κB, increases the production of β-secretase and γ-secretase, thereby enhancing Aβ generation (Sun et al., 2014; Yamamoto et al., 2007). IL-10 and transforming growth factor-β (TGF-β) participate in anti-inflammatory responses and immune suppression. IL-6 exhibits dual functions, acting as either a pro-inflammatory or anti-inflammatory cytokine depending on its concentration and microenvironment (Scheller et al., 2011). TGF-β displays neuroprotective effects by inhibiting Aβ production and deposition, alleviating neuroinflammation, and suppressing GSK-3β-mediated tau phosphorylation (Caraci et al., 2012).

#### Mechanisms of *G. elata* and its active ingredients in alleviating AD-related inflammation

*G. elata* has the potential to alleviate Alzheimer‘s disease (AD) pathology. FGGE (a fermented product of *G. elata*) improves the behavioral performance of AlCl3-induced AD models, an effect primarily associated with enhanced antioxidant stress, alleviated hippocampal inflammation, regulation of gene expression, and modulation of the gut microbiota (Huang et al., 2025). Another processed product, FGE, improves neurotransmitter release by increasing acetylcholine (ACh) and choline acetyltransferase (ChAT) content in brain tissue, alleviates oxidative stress damage (restores SOD activity), reduces D-Gal/AlCl_3_-induced AD pathological changes (Aβ, Tau, p-Tau), and ameliorates cognitive function and anxiety-like behavior (Wang et al., 2024). Of note, during the inflammatory resolution phase, macrophages upregulate ChAT expression via the MyD88 pathway, and the acetylcholine they produce promotes neutrophil clearance (Luo et al., 2024).

In Aβ_42_-induced SH-SY5Y cells, gastrodin downregulates ADRA1/NF-κB/NLRP3 protein expression, thereby reducing the release of IL-1β and IL-18 (Li B. et al., 2025). Additionally, gastrodin restores tight junction protein expression in bEnd.3 cells, improves blood–brain barrier (BBB) integrity, and prevents further exacerbation of neuroinflammation (Mou et al., 2022). Furthermore, gastrodin inhibits the expression of FERMT2 in astrocytes as well as the inflammatory factors TNF-α, IL-6, and MMP8, thereby maintaining normal extracellular matrix viscoelasticity and ensuring BBB structural integrity (Wang L. et al., 2025). In Tg2576 mice, gastrodin increases SOD and CAT activities, reduces MDA and ROS levels, and downregulates BACE1 expression by inhibiting the PKR/eIF2α pathway, consequently decreasing Aβ generation (Zhang et al., 2016). Moreover, *G. elata* reduces Akt/GSK-3β-related tau hyperphosphorylation, which may fundamentally mitigate the occurrence of inflammation (Li et al., 2026). In LPS-induced AD models, gastrodin has also been shown to inhibit microglial activation through TLR4/TRAF6/NF-κB pathway suppression (Wang et al., 2024).

In addition to gastrodin, other components of *G. elata* also participate in counteracting neuroinflammation and inflammatory damage during AD progression. The ethyl acetate extract of *G. elata* (EEGE) alleviates oxidative stress and inhibits Aβ aggregation in Caenorhabditis elegans, an effect associated with IIS-regulated genes such as hsp-12.3 and hsp-12.6 (Shi et al., 2023). The IIS pathway involves three transcription factors: SKN-1, DAF-16, and HSF-1. Both 4,4′-methylenediphenol and another compound (likely referring to HBA, as per the original citations) promote DAF-16 nuclear translocation, upregulate the expression of SKN-1, SOD-3, and GST-4, thereby enhancing antioxidant stress capacity and inhibiting Aβ toxicity or aggregation (Yu et al., 2024a, b). HBA also increases Nrf2 and neurotrophic factors (BDNF and GDNF) while reducing inflammatory factors such as TNF-α and IL-1β, thereby preventing Aβ_42_ oligomer-induced synaptic and cognitive deficits (Ding et al., 2019) *G. elata* polyphenols (GPP) exhibit antioxidant and cytoprotective effects in PC12 cells (Tan et al., 2022).

*G. elata*-specific miRNAs, including Gas-miR01 and Gas-miR02, regulate inflammatory responses by targeting the NF-κB pathway key protein A20 and downregulating its expression (Xia et al., 2020). Gas-miR36-5p targets GSK-3β, inhibits tau hyperphosphorylation, and ameliorates oxidative stress, which may explain its role in preventing the progression of inflammation and associated damage (Lu et al., 2023). These findings suggest that *G. elata* not only acts through its small-molecule components but also via its miRNAs in cross-kingdom regulation. In addition to its anti-inflammatory mechanisms, *G. elata* components can directly intervene in Aβ aggregation and tau pathology, working synergistically to form a multi-target strategy against AD.

### Acute ischemic stroke

#### Inflammation in acute ischemic stroke

Acute ischemic stroke (AIS) is the most common type of stroke, accounting for approximately 62.4%–87% of all stroke cases worldwide and representing a major global public health burden (Moskowitz et al., 2010). Ischemia/reperfusion (I/R) injury exacerbates brain damage and neurological deficits (Xiong et al., 2018). The primary goal of treatment is tissue salvage, as the ischemic penumbra retains salvage potential compared to the ischemic core (Moskowitz et al., 2010). However, reperfusion of the penumbra introduces multiple challenges, including excitotoxicity and inflammation (Anrather and Iadecola, 2016). The development of neuroinflammation significantly contributes to neuronal injury during the acute phase of cerebral ischemia (Wu et al., 2020), and neuroinflammatory mechanisms are closely intertwined with oxidative stress (Wong and Crack, 2008).

Following ischemic stroke, the inflammatory cascade is activated immediately after vascular occlusion (De Meyer et al., 2016; Iadecola and Anrather, 2011). Blood stasis and rheological changes induce the expression of P-selectin on vascular endothelium and platelets, further exacerbating ischemic injury. Acute oxygen-glucose deprivation (OGD) leads to substantial ATP accumulation, thereby increasing NADPH oxidase-dependent reactive oxygen species (ROS) production (De Meyer et al., 2016). ROS directly damage cellular structures and upregulate the expression of pro-inflammatory factors (e.g., IL-1β, TNF-α) while downregulating the expression of protective genes (e.g., PI3K, Akt, PKC, CAMKII), thereby aggravating injury (Turovsky et al., 2021).

Simultaneously, necrotic neurons release damage-associated molecular patterns (DAMPs, or “danger signals”), which activate microglia via TLR4 and stimulate the NF-κB pathway to produce inflammatory cytokines (Yang et al., 2019). DAMPs can also be released directly by immune cells: ATP is released by neutrophils during neutrophil extracellular trap (NETosis) formation, and double-stranded DNA (dsDNA)—which is normally confined to the nucleus of intact cells—is released into microglia and astrocytes within three days after middle cerebral artery occlusion (MCAO) (Li et al., 2020) dsDNA activates microglia through the cGAS–STING pathway, promoting microglial pyroptosis, production of TNF-α and IL-6, and neutrophil infiltration. The NLRP3 inflammasome is a widely studied node in pro-inflammatory signaling (Ozaki et al., 2015; Tuttolomondo et al., 2020). Upon activation, microglia exhibit distinct gene expression profiles: the classical M1 phenotype exerts pro-inflammatory effects, whereas the M2 phenotype displays anti-inflammatory properties (Prinz and Priller, 2014).

Although the regenerative capacity of the central nervous system (CNS) is limited, neurogenesis can still occur in the subventricular zone (SVZ) and the subgranular zone (SGZ) of the dentate gyrus (Jin et al., 2006). Following ischemic stroke, neural stem cells migrate to the peri-infarct region and differentiate into neurons (Zhang et al., 2001). However, in an inflammatory microenvironment lacking trophic factors, many immature neurons fail to survive (Arvidsson et al., 2002). Brain-derived neurotrophic factor (BDNF) plays a critical role in development and exerts neuroprotective functions primarily by binding to tyrosine kinase receptors and activating the phosphatidylinositol 3-kinase (PI3K)/Akt and mitogen-activated protein kinase/extracellular signal-regulated kinase (MAPK/ERK) pathways. These pathways activate transcription factors such as AP1, NF-κB, and FOXOs, and induce the expression of genes encoding antioxidant enzymes, anti-apoptotic proteins, and proteins involved in ion homeostasis (Mattson, 2008).

#### Mechanisms of *G. elata* and its active ingredients in alleviating inflammation in acute ischemic stroke

*G. elata* extract has demonstrated multiple protective effects in cerebral ischemia models. It promotes microglial polarization from the M1 to the M2 phenotype and attenuates the inflammatory cascade by inhibiting the JNK/TLR4/T3JAM/NF-κB signaling pathway (Huang et al., 2024). The protein extract of *G. elata* (GEPS) counteracts MCAO-induced inhibition of the PI3K/AKT pathway and activation of the MAPK signaling pathway, increases the expression of BDNF and GAP43 in the penumbra, and protects neurons (Duan et al., 2023). The ethanol extract of *G. elata* (GEE) activates the Nrf2 pathway by promoting AKT phosphorylation, thereby exerting antioxidant and anti-ferroptosis effects and ameliorating cerebral ischemic injury (Wang et al., 2026).

Gastrodin, the most extensively studied active ingredient of *G. elata*, exhibits multiple effects in ischemia/reperfusion (I/R) models. It significantly inhibits I/R-induced upregulation of the pro-inflammatory cytokine IL-1β, reduces the expression of pro-oxidant enzymes COX-2 and iNOS in the ischemic brain, and alleviates neuronal apoptosis by suppressing the caspase-3-mediated apoptotic signaling pathway (Liu et al., 2016). *In vitro* experiments have shown that gastrodin stabilizes mitochondrial membrane potential, increases ATP content, and reduces DNA leakage, thereby inhibiting TLR9-mediated production of inflammatory factors (TLR9, IL-6, TNF-α, and IL-1β). Moreover, gastrodin enhances its anti-inflammatory and neuroprotective effects by inhibiting the JAK2/STAT3 signaling pathway (Zhang et al., 2024a). It also increases P62 expression, decreases Keap1 expression, and promotes Nrf2 nuclear translocation and binding to ARE, thereby exerting antioxidant effects, counteracting acute injury, and preventing neuronal apoptosis (Wu et al., 2025). Additionally, gastrodin significantly inhibits Zn^2+^-induced cell death, a mechanism associated with the suppression of p67 expression and PAR formation in astrocytes (Luo et al., 2018a).

The phenolic components of *G. elata* (mainly 4-hydroxybenzaldehyde and 4-hydroxybenzyl alcohol) upregulate the expression of HO-1, NQO-1, and BDNF by activating the Nrf2/ARE pathway, thereby enhancing endogenous antioxidant capacity and improving the prognosis after cerebral ischemia-reperfusion in rats (Shi et al., 2018), 3,4-Dihydroxybenzaldehyde (DBD) restores mitochondrial permeability transition pore (mPTP) levels by enhancing neural mitochondrial respiratory chain function, reduces the loss of mitochondrial membrane potential (MMP) and the translocation of cytochrome c to the cytoplasm, ameliorates mitochondrial dysfunction, and inhibits apoptosis (Luo et al., 2023). p-Hydroxybenzaldehydealso exerts mitochondrial protective effects; it can be converted into the metabolite 4-hydroxybenzoic acid through oxidative metabolic pathways in the brain. Its protective mechanism against blood–brain barrier oxidative stress injury involves capturing/clearing reactive oxygen species and forming stable intermediates to terminate free radical chain reactions (Feng et al., 2025a). 4-Hydroxybenzyl alcohol increases the resistance of astrocytes and neurons to Zn^2+^ toxicity, thereby exerting neuroprotective effects after cerebral ischemia (Luo et al., 2018b).

In addition to gastrodin and the aforementioned phenolic compounds, other components of *G. elata* also exhibit distinct anti-inflammatory effects. *G. elata* neutral polysaccharides inhibit ferroptosis-mediated neuroinflammation by upregulating NRF2/HO-1 expression and promoting NRF2 nuclear translocation (Zhang et al., 2024b). Parishin C alleviates cerebral ischemia-induced brain tissue injury by reducing oxidative stress and inflammatory responses (Wang et al., 2021).

### Vascular dementia

#### Inflammation in vascular dementia

Vascular dementia (VaD) is a major type of dementia comparable to Alzheimer’s disease (AD), accounting for approximately 15%–30% of all dementia cases and representing the second most common form of dementia after AD. With the acceleration of global population aging, the disease burden of VaD continues to increase (Wolters and Ikram, 2019). The etiology of VaD is highly heterogeneous, with causes including cerebral ischemia, cerebral hemorrhage, small vessel lesions, and toxic substance deposition; consequently, its research models are diverse (Hort et al., 2019). Although the initiating factors vary, most studies have observed early emergence of inflammatory responses and elevated oxidative stress markers, suggesting that neuroinflammation and oxidative damage are common pathways in VaD progression. Therefore, reducing inflammation and oxidative stress represents a feasible therapeutic strategy for VaD.

Acute and chronic cerebrovascular injuries are major triggers of VaD-related neuroinflammation. Hypoperfusion-induced ischemia and hypoxia promote oxidative stress (Ionescu-Tucker and Cotman, 2021), generate large amounts of reactive oxygen species (ROS), and activate glial cells (Sha et al., 2021). Long-term activated microglia release pro-inflammatory factors such as IL-1β, IL-6, and TNF-α, leading to mitochondrial dysfunction and neuronal injury (Lyman et al., 2014).

Neuroinflammation also disrupts endothelial tight junctions and impairs blood–brain barrier (BBB) integrity. In chronic hypoperfusion models, white matter injury upregulates MMP2 expression (Simons and Nave, 2015), MMP2 released by endothelial cells and glial cells subsequently alters tight junction structures, inducing BBB injury (Sha et al., 2021; Yang et al., 2007). Damaged BBB promotes infiltration of inflammatory mediators into the brain parenchyma, creating an inflammatory amplification cycle and exacerbating white and gray matter damage (Tian et al., 2022). Inhibition of MMP2 reduces BBB disruption, microglial activation, and white matter lesions (Narantuya et al., 2010).

Vascular dementia often overlaps with Alzheimer’s disease pathology. The APOEε4 allele is associated with the risk of both disorders (Esiri et al., 2015). accelerate Aβ deposition and accumulation of damage-associated molecular patterns (DAMPs), and induce neuroinflammation (Achariyar et al., 2016), Mutations in the APOEε4 gene impair lymphatic function (Taoka et al., 2017). Additionally, vascular risk factors promote the upregulation of VCAM-1 and ICAM-1 on endothelial cells, facilitate the infiltration of leukocytes into the brain parenchyma, and further aggravate various inflammatory lesions (e.g., excitotoxicity, glial scarring, chronic hypertension, microbleeds, and perivascular space enlargement), collectively driving the progression of VaD (Jayaraj et al., 2019; Pober and Sessa, 2014).

#### Mechanisms of *G. elata* and its active components in intervening in VaD inflammation

*G. elata* and its active components regulate inflammation, oxidative stress, Aβ metabolism, and blood–brain barrier (BBB) integrity through multiple targets, demonstrating clear protective effects in vascular dementia (VaD) models. The ethyl acetate extract of *G. elata* (EEGE) effectively downregulates serum levels of IL-6, TNF-α, and IL-1β by modulating the IGF1-TREM2 signaling axis and the AMPK-SIRT1-FoxO1-NF-κB pathway, thereby exerting antagonistic effects against VaD (Tao et al., 2026).

Gastrodin (GAS) is the most thoroughly studied active ingredient. GAS inhibits OGD-stimulated astrocyte activation and inflammasome formation in MCAO-reactive astrocytes by suppressing the STAT3 and NF-κB signaling pathways, and reduces the expression of caspase-1 and IL-18 (Sui et al., 2019). It also inhibits the NOTCH-1 signaling pathway and decreases iNOS and TNF-α expression in activated microglia (Guo et al., 2021), while simultaneously inhibiting the RAS pathway (except AT2 and SIRT3) and the pro-inflammatory mediator TNF-α to exert neuroprotective effects (Liu et al., 2018b).

GAS reduces Aβ_1–40/42_, APP, and BACE1 levels, increases ADAM10 and IDE expression, thereby decreasing Aβ generation and promoting its clearance, and antagonizes VaD-associated cognitive impairment (Liu et al., 2018c; Shi et al., 2020) HBA also alleviates these pathological changes. Furthermore, GAS and HBA improve energy metabolism disorders (glycolysis, tricarboxylic acid cycle, and pentose phosphate pathway) and alleviate oxidative damage-induced mitochondrial dysfunction (Wu et al., 2023).

Both GAS and HBA exert antioxidant effects through the Nrf2 pathway and mitochondrial protection; notably, GAS activates the Nrf2/Keap1-GPx4 axis to inhibit ferroptosis in hippocampal neurons (Li et al., 2022), while HBA regulates the choline-PLD2/Sirt1/PGC-1α pathway to maintain mitochondrial homeostasis (Li and Zhang, 2015; Wu et al., 2023). Furthermore, activation of the Nrf2/Keap1-GPx4 signaling pathway inhibits ferroptosis in hippocampal neurons, representing another protective mechanism of GAS (Li et al., 2022).

In VaD, chronic hypoperfusion-induced ischemia and hypoxia promote oxidative stress via TLR2/MyD88/NF-κB signaling cascade activation (Chen et al., 2024). Neuroinflammation disrupts endothelial tight junctions and impairs BBB integrity through upregulation of MMP2 and MMP9 expression. Damaged BBB promotes infiltration of inflammatory mediators into the brain parenchyma, creating an inflammatory amplification cycle and exacerbating white and gray matter damage (Jayaraj et al., 2019). Ferroptosis-mediated neuroinflammation is closely associated with white matter injury—oligodendrocytes are highly sensitive to ferroptosis, and their damage directly leads to white matter integrity disruption and cognitive decline. GAS, through activation of the Nrf2/Keap1-GPx4 signaling pathway, may suppress ferroptosis in hippocampal neurons, thereby potentially protecting both gray matter neurons and white matter integrity (Li et al., 2022). VaD often overlaps with Alzheimer’s disease pathology. In the BCCAO-induced VaD rat model, gastrodin has been shown to improve cognitive deficits by reducing Aβ1-40/1-42 levels and regulating Aβ-related proteins (Liu et al., 2018b). Additionally, gastrodin alleviates tau hyperphosphorylation through the AKT/GSK-3β signaling pathway in cellular models (Li et al., 2026). These findings suggest that gastrodin may exert synergistic effects on both vascular and neurodegenerative pathologies in VaD-AD mixed pathology. GAS pretreatment reduces the number of positive cells and the content of matrix metalloproteinase 2 (MMP2) and matrix metalloproteinase 9 (MMP9) following nerve injury, significantly improves neurobehavioral deficits, reduces cerebral infarct volume, reverses BBB dysfunction, and alleviates inflammation (Li et al., 2019) 4-HBd exhibits a marked brain-targeting effect and maintains BBB integrity through the RNS/ROS-MMP-TJ signaling pathway (Feng et al., 2025b). Notably, focused ultrasound (FUS)-mediated BBB opening combined with GAS injection alleviates memory deficits and neuropathology in AD-like model mice (Luo et al., 2022), suggesting that this combined strategy holds potential application value in VaD-AD mixed pathology.

### Epilepsy

#### Inflammation in epilepsy

Epidemiological data show that approximately 51.7 million people worldwide suffer from epilepsy. In 2021, there were about 3.3 million new cases of idiopathic epilepsy (with a 95% confidence interval of 2.4 million to 4.1 million cases). The age-standardized incidence rate was 42.8 cases per 100,000 people (31.2–53.7 cases) (GBD Epilepsy Collaborators, 2025). The causes of epilepsy are highly heterogeneous. They can result from genetic susceptibility or be caused by structural lesions such as brain trauma, infection, tumor, and stroke. However, a large amount of evidence in recent years has shown that regardless of the initiating factors, neuroinflammation is a common pathological pathway for the occurrence and progression of epilepsy (Ravizza et al., 2024). Any factor that causes disorders in the physiological structure or function of the brain may lead to epileptic seizures. This article focuses on structural/metabolic epilepsy caused by central nervous system (CNS) infections or autoimmunity, which can occur in all age groups (Nissen et al., 2020). Viral infections can induce post-infection encephalitis or encephalomyelitis, which in turn can lead to autoimmune encephalitis mediated by T cells and/or autoantibodies. Abnormalities in the glutamatergic and GABAergic neurotransmitter systems are important links in the onset of epilepsy.

After the occurrence of non-infectious epileptic events and before epileptic seizures, endogenous inflammation-related factors (mediating factors DS/DAMPs and effector factors IL-1β, TNF-α, IL-6, etc.) are induced in the BBB endothelium. A large amount of experimental evidence indicates that the activation of inflammatory signaling pathways is directly involved in the occurrence of epilepsy. Genetic ablation or drug inhibition of TLR3, TLR4, or IL-1R1 can significantly reduce acute or chronic recurrent epileptic seizures in animal models, and can improve disease outcomes when applied before epileptic seizures. Furthermore, during the occurrence of epilepsy, antagonizing COX-2 and prostaglandin E2 can alleviate cell loss and reduce comorbidity (Perucca et al., 2023).

#### Mechanisms of *G. elata* and its active components in counteracting epilepsy-related inflammation

*G. elata* and its active components exert antiepileptic effects in various epilepsy models by regulating neuroinflammation, the GABAergic system, and glutamatergic excitotoxicity. *G. elata* inhibits astrocyte activation via modulation of the mTOR signaling pathway, thereby exerting antiepileptic effects (Yip et al., 2020). Gastrodin exerts its antiepileptic effects by inhibiting MAPK/NF-κB signaling, reducing IL-1β and TNF-α while increasing IL-10 levels (Chen et al., 2017). It also targets P2RY12 to inhibit excessive microglial activation (Cai et al., 2026) and regulates the NOX2/Nrf2 balance to alleviate neuroinflammation. Parishin B exerts antiepileptic effects through coordinated inhibition of neuroinflammation, regulation of neurotransmitter homeostasis, and Acly-related energy-lipid metabolism (Sun et al., 2026).

GABA receptor signaling, particularly the GABAA receptor subtype, mediates the antiepileptic protection of *G. elata* (Shin et al., 2011). Gastrodin reverses GABAA receptor degradation following pilocarpine-induced seizures (Yang et al., 2021). Additionally, 4-hydroxybenzaldehyde and 4-hydroxy-3-methoxybenzaldehyde strongly inhibit GABA transaminase (GABA-T), whereas 4-hydroxybenzyl alcohol does not exhibit a significant effect (Ha et al., 2001). GABA receptors also regulate central immune functions, including cell migration, cytokine secretion, and immune cell activation (Bhandage and Barragan, 2021). The antiepileptic effect of *G. elata* is closely linked to its modulation of the GABAergic system. 4-Hydroxybenzaldehyde, a major phenolic constituent of *G. elata*, exhibits GABA transaminase (GABA-T) inhibitory activity superior to that of the known anticonvulsant valproic acid (Ha et al., 2001). A novel citryl glycoside isolated from *G. elata* also shows GABA-T inhibitory effects (Choi and Lee, 2006). In the hippocampus of seizure-sensitive gerbils, gastrodin significantly decreased the immunoreactivities of GABA-T, succinic semialdehyde dehydrogenase (SSADH), and succinic semialdehyde reductase (SSAR)—key enzymes in the GABA shunt—suggesting that gastrodin may elevate GABA concentration, at least in part, through inhibition of the GABA shunt (An et al., 2003). The interplay between the GABAergic system and inflammation is bidirectional: GABA receptor activation suppresses excessive immune cell activation, while inflammatory cytokines (such as TNF-α and IL-1β) downregulate GABA receptor expression. Thus, *G. elata* components may form a synergistic antiepileptic protective network by simultaneously enhancing GABAergic transmission and suppressing neuroinflammation. Parishin B, one of the major bioactive components of *G. elata*, has recently been evaluated in a PTZ-induced zebrafish epilepsy model. Parishin B significantly reduced PTZ-induced seizure activity and hyperlocomotion without apparent toxicity. Mechanistically, it restored neurotransmitter balance (including GABA, dopamine, and norepinephrine), inhibited neuroinflammation, enhanced antioxidant capacity, and modulated TCA cycle, lipid metabolism, and ACLY-related pathways as revealed by multi-omics analysis (Sun et al., 2026).

Inhibition of glutamate-induced apoptosis and intracellular calcium elevation is considered a potential mechanism by which vanillin and 4-hydroxybenzaldehyde exert antiepileptic effects in IMR-32 human neuronal cells (Lee et al., 1999). The methanol extract of *G. elata* exerts neuroprotective and antioxidant effects by upregulating the PI3K signaling pathway and reducing glutamic acid-induced ROS levels (Han et al., 2014).

GEAT decoction significantly reduces the severity of epileptic seizures, decreases the accumulation of the inflammatory mediators TNF-α, IL-1β, and IL-6, alleviates oxidative stress, and ameliorates pentylenetetrazole (PTZ)-induced anxiety-like behaviors and cognitive deficits (He et al., 2023). *G. elata* in combination with *U. rhynchophylla* or *Acorus calamus* also reduces seizure occurrence (Hsieh et al., 1999) Furthermore, *G. elata* can enhance the effects of other drugs: co-treatment of *G. elata* extract with valproic acid (VPA) improves drug-resistant epilepsy by regulating the OATP transporter and increasing VPA levels in the brain (Yang and Tsai, 2022). *G. elata* extract also shows synergistic effects with conventional antiepileptic drugs. Yang and Tsai (2022) demonstrated that co-administration of *G. elata* extract with valproic acid (VPA) improved drug-resistant epilepsy in a pilocarpine-induced rat model by increasing brain VPA levels via organic anion transporting polypeptide (OATP) regulation. The combination exhibited synergistic anticonvulsant effects, protecting hippocampal neurons and altering DOPAC and 5-HT levels (Yang and Tsai, 2022).

Parishin B exerts antiepileptic effects through coordinated inhibition of neuroinflammation, regulation of neurotransmitter homeostasis, and ACLY-associated energy-lipid metabolism (Zhao et al., 2026).

## Evidence attribution statement

Among the included studies, a subset employed herbal compound formulas (e.g., *G. elata* with *U. rhynchophylla*, GEAT decoction) or co-interventions combining gastrodin/parishin with other active agents (e.g., valproic acid). These interventions are categorized as Type B studies in the present analysis. For Type B studies, the observed pharmacological effects—including anti-neuroinflammatory responses, improvements in behavioral scores, and modulation of signaling pathways—cannot be exclusively or unequivocally attributed to *G. elata* or its monomeric constituents. Therefore, in the subsequent mechanistic synthesis and evidence integration, findings from Type B studies are not employed as direct evidence for establishing the specific mechanisms of action of *G. elata*. Instead, they are interpreted as supportive auxiliary evidence that corroborates the potential efficacy of *G. elata*-related intervention strategies under complex pathological conditions.

### Risk of bias within the included studies

Across the 44 currently assessed studies, the overall risk of bias was unclear in 43 studies (97.7%) and high in one study (2.3%); no study met the prespecified criteria for overall low risk. The dominant pattern was therefore incomplete methodological reporting rather than consistent evidence of low-risk study conduct.

The most substantial reporting gaps concerned core safeguards against selection and performance bias. Sequence generation, baseline characteristics, allocation concealment, and random housing were judged as unclear in all 44 studies (100%). For blinding of caregivers and/or investigators, 42 studies (95.5%) were unclear, one study (2.3%) was low risk, and one study (2.3%) was high risk. Random outcome assessment was unclear in 43 studies (97.7%) and low risk in one study (2.3%).

Detection, attrition, and reporting bias were also predominantly unclear. Blinding of outcome assessors was unclear in 39 studies (88.6%), low risk in four studies (9.1%), and high risk in one study (2.3%). Incomplete outcome data were unclear in 43 studies (97.7%) and low risk in one study (2.3%). Selective reporting was unclear in 43 studies (97.7%) and high risk in one study (2.3%). In contrast, other sources of bias were judged as low risk in 41 studies (93.2%) and unclear in three studies (6.8%), largely because most publications reported ethical approval, public funding, and/or no competing interests.

Low-risk judgements outside the “other sources of bias” item were isolated. One study reported blinded administration, one reported random selection of animals for outcome assessment, four reported blinded outcome assessment, and one provided sufficiently complete animal accounting to be judged at low risk for incomplete outcome data. These isolated safeguards did not result in an overall low-risk judgment because key information on randomization, allocation concealment, housing, and other methodological procedures was missing.

The only study judged at overall high risk explicitly stated that the authors were aware of group allocation, experimental conduct, and outcome assessment. In addition, a prespecified 4-h maximal electroshock endpoint described in the Methods was not reported in the corresponding results table. This study was therefore judged at high risk for performance bias, detection bias, and selective reporting.

### Clinical translation landscape of *G. elata* bioactive constituents

Despite growing preclinical evidence, clinical research on *G. elata* remains confronted with substantial bottlenecks. The vast majority of available studies are preclinical in nature, with only a limited number of clinical investigations having been conducted to date—and among these, the overwhelming majority have focused exclusively on the monomeric component gastrodin. This pronounced disparity between preclinical and clinical evidence may account for the currently incomplete understanding of the structure–activity relationship and the lack of a clear correlation between the complex phytochemical profile of *G. elata* and its observed pharmacological effects (Zhou X. et al., 2025). Currently registered clinical studies on *G. elata* fall broadly into two categories: those investigating the traditional decoction formulation and those evaluating gastrodin as a single agent. The intervention modalities for gastrodin are diverse, encompassing intravenous infusion and local acupoint injection. Nevertheless, these studies are generally limited in sample size and predominantly assess combinatorial therapeutic regimens, with a notable paucity of high-quality evidence-based medicine validation for monotherapy use.

Despite the aforementioned challenges, emerging clinical evidence has begun to validate the anti-inflammatory and neuroprotective potential of *G. elata* observed in preclinical settings. In a randomized, double-blind, placebo-controlled trial involving patients undergoing coronary artery bypass grafting (CABG), perioperative infusion of gastrodin significantly reduced the incidence of postoperative delirium and was associated with improved discharge outcomes. These findings provide preliminary clinical corroboration of the neuroprotective effects of gastrodin, although larger-scale studies are warranted to further substantiate its efficacy (Bai et al., 2025). The majority of registered clinical investigations have also concentrated on cognitive impairment, depression, and other brain disorders, with certain trials having progressed to Phase 4 (Table 2). The research foundation established for gastrodin may facilitate the entry of additional *G. elata*-derived components into clinical evaluation. Concurrently, various strategies have been explored to address the low clinical translation rate of *G. elata*, a challenge largely attributable to its poor blood–brain barrier (BBB) permeability. In this regard, focused ultrasound (FUS) has been employed to enhance the BBB penetration of gastrodin, ultimately improving therapeutic outcomes in Parkinson’s disease models (Wang et al., 2022). Wang et al. (2007) demonstrated that gastrodin can be transported directly into the brain via the olfactory pathway following intranasal administration, achieving non-invasive brain-targeted delivery (Wang et al., 2007).

*G. elata* and *U. rhynchophylla* are commonly prescribed together in traditional Chinese medicine practice. Notably, *U. rhynchophylla* shares key pharmacokinetic limitations with *G. elata*, including low oral bioavailability and poor blood–brain barrier (BBB) permeability. Consequently, formulation strategies developed to enhance the brain delivery of rhynchophylline—the major bioactive alkaloid of *U. rhynchophylla*—may offer valuable insights applicable to the clinical translation of *G. elata* constituents. Indeed, rhynchophylline encapsulated in Tween 80-coated mPEG-PLGA nanoparticles has been demonstrated to effectively cross the BBB and exert significant neuroprotective effects (Xu et al., 2020). Furthermore, the intranasal administration of rhynchophylline via a thermosensitive gel formulation (Lin et al., 2023) provides a novel reference approach for targeted therapy of *G. elata* in Parkinson’s disease and other central nervous system disorders.

## Summary and discussion

### Comparison of common mechanisms and differences across diseases

A useful way to conceptualize the mechanistic evidence assembled in this review is to consider three distinct levels of disease specificity (Figure 3).

**FIGURE 3:**
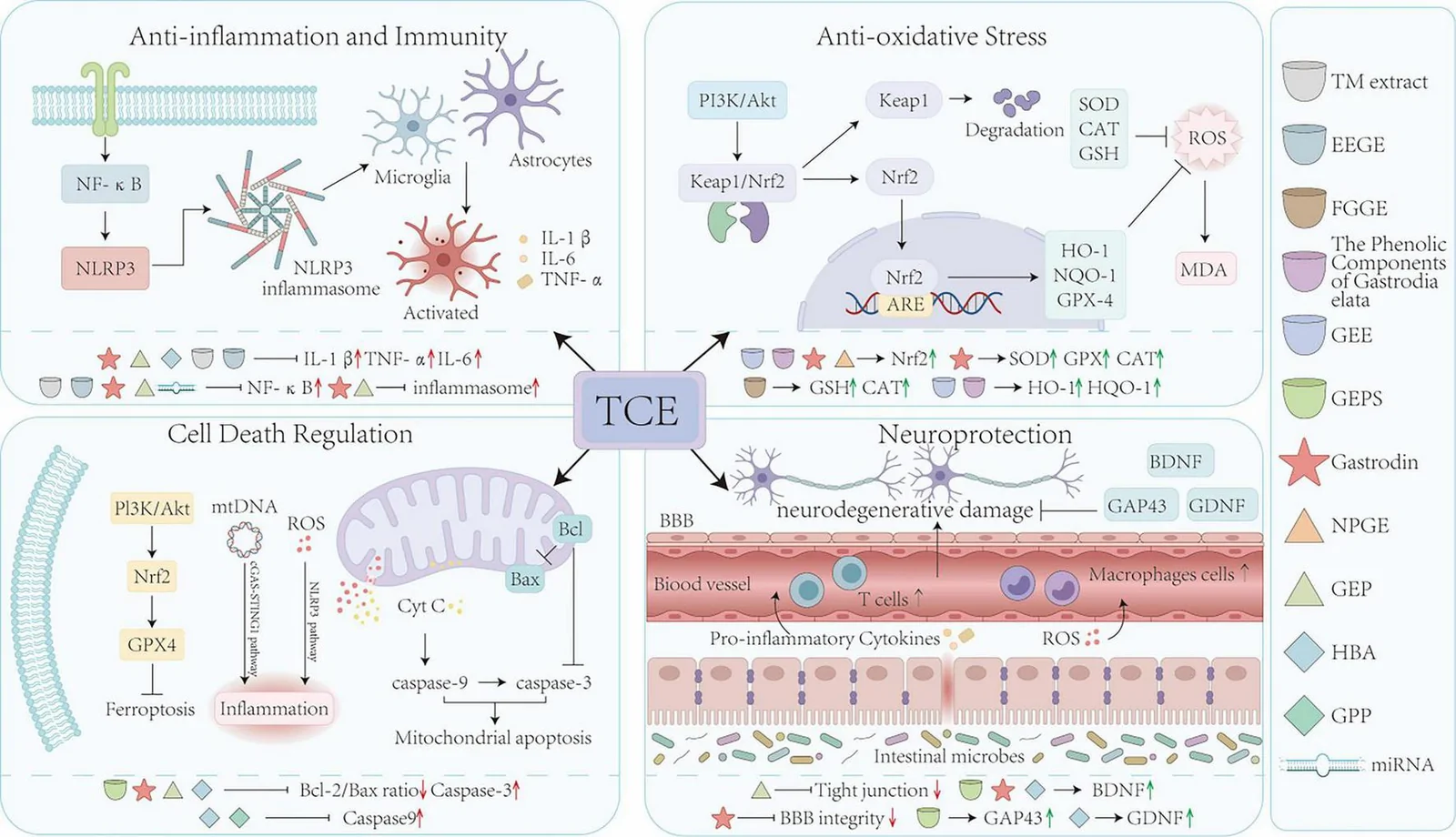
Possible mechanisms of *Gastrodia elata* and its active components in the intervention of neurological diseases.

At the broadest level, certain pathways appear to represent core inflammatory hubs that are engaged across multiple disease contexts. The NF-κB/NLRP3 inflammasome axis stands out as the most consistently documented across all five disorders. Its upstream triggers are disease-specific—α-synuclein aggregates in Parkinson’s disease, amyloid-β in Alzheimer’s disease, damage-associated molecular patterns in ischemic stroke, chronic hypoperfusion in vascular dementia, and seizure activity in epilepsy—yet these divergent initiators converge on a common downstream inflammatory cascade. Likewise, the Nrf2/HO-1 antioxidant pathway is repeatedly implicated in Parkinson’s disease, ischemic stroke, and vascular dementia, where it serves to counteract oxidative stress-driven neuroinflammation.

At an intermediate level, some mechanisms exhibit a clear disease-preferential distribution. The MAPK/ERK/CREB axis, for instance, is prominently engaged by gastrodin in Parkinson’s disease models to support dopaminergic neuronal homeostasis, whereas in epilepsy the same MAPK module is suppressed to attenuate seizure-induced inflammation—a finding that underscores the functional plasticity of this signaling cascade in response to different pathological milieus. The GABAergic system, by contrast, is predominantly modulated in epilepsy models, where *G. elata*-derived compounds enhance inhibitory neurotransmission through GABA transaminase inhibition.

At the exploratory level, certain frameworks remain insufficiently validated across diseases. The so-called “microbiota–metabolism–epigenetics” regulatory axis, although mechanistically attractive, is currently supported chiefly by studies in Parkinson’s and Alzheimer’s diseases, with comparatively little evidence in ischemic stroke, vascular dementia, or epilepsy. This construct is therefore best viewed as a promising working hypothesis rather than an established mechanism, and its relevance across the full spectrum of neurological disorders examined here warrants dedicated investigation.

A synthesis of the anti-inflammatory mechanisms of *G. elata* and its bioactive constituents reveals that the NF-κB/NLRP3, Nrf2/HO-1, and MAPK signaling pathways have been extensively documented across the five neurological diseases examined in this review. Nevertheless, the upstream activating signals, intervention nodes, and downstream effector responses associated with these pathways exhibit marked divergence, primarily attributable to the distinct pathological features inherent to each disease.

The NF-κB/NLRP3 pathway serves as the central inflammatory amplification hub across the neurological disorders examined in this review. In Parkinson’s disease, α-synuclein aggregates trigger NF-κB signaling via TLR4 activation, leading to upregulation of NLRP3 inflammasome components, an effect counteracted by *G. elata* polysaccharides (GEP) through TLR4/NF-κB inhibition, which reduces IL-1β, IL-6, and TNF-α production (Gan et al., 2024). In Alzheimer’s disease, amyloid-β (Aβ) drives neuroinflammation through the ADRA1/NF-κB/NLRP3 axis, and gastrodin directly suppresses ADRA1 expression (Li B. et al., 2025), thereby blocking both NF-κB nuclear translocation and NLRP3 inflammasome assembly. In acute ischemic stroke, damage-associated molecular patterns (DAMPs) activate NF-κB via TLR4, whereas *G. elata* extract promotes microglial M1-to-M2 polarization through inhibition of the JNK/TLR4/T3JAM/NF-κB cascade (Huang et al., 2024). In epilepsy, gastrodin attenuates IL-1β and TNF-α levels via MAPK/NF-κB pathway suppression (Chen et al., 2017). In vascular dementia, chronic hypoperfusion-induced oxidative stress drives inflammatory responses through the TLR2/MyD88/NF-κB axis (Chen et al., 2024), while gastrodin confers neuroprotection by inhibiting STAT3 and NF-κB signaling (Sui et al., 2019).

The Nrf2/HO-1 pathway is a core effector mechanism underlying the combined antioxidant and anti-inflammatory activities of *G. elata*. In ischemic stroke, *G. elata*-derived neutral polysaccharides activate Nrf2/HO-1 signaling to counteract ferroptosis-driven neuroinflammation (Zhang et al., 2024b). In Parkinson’s disease, the water extract of *G. elata* exerts protective effects through restoration of Akt/GSK3β/Nrf2 pathway activity (Lin et al., 2021). In vascular dementia, gastrodin activates the Nrf2/Keap1-GPx4 axis to inhibit ferroptosis in hippocampal neurons (Li et al., 2022). Of particular interest, the upstream regulatory logic of Nrf2 activation diverges according to disease context: PI3K/Akt-dependent signaling predominates in ischemic injury, whereas neurodegenerative conditions engage more complex cross-regulatory networks involving the DAF-16/insulin signaling pathway (Yu et al., 2024a).

The MAPK pathway, encompassing ERK, JNK, and p38, exhibits functional duality in mediating the anti-inflammatory effects of *G. elata*. In epilepsy, gastrodin exerts its anticonvulsant effect partly through inhibition of MAPK (notably ERK1/2) phosphorylation and suppression of CREB activation, thereby mitigating neuroinflammation (Chen et al., 2017). In ischemic stroke, *G. elata* extract disrupts the TLR4/NF-κB cascade via JNK phosphorylation inhibition (Huang et al., 2024). In stark contrast, in Parkinson’s disease, gastrodin engages the MEK/ERK/CREB axis to upregulate VMAT2 expression and sustain dopamine homeostasis—an effect that is mechanistically converse to MAPK inhibition. Thus, the MAPK pathway appears to mediate context-dependent and cell type-specific responses to *G. elata* treatment, with the ultimate biological outcome—whether neuroprotection, anti-inflammation, or survival promotion—being shaped by the pathological milieu and the prevailing upstream signaling input (Zhao et al., 2024).

Collectively, the foregoing comparative analysis delineates a compelling principle: rather than uniformly suppressing or activating any given signaling cascade, the bioactive components of *G. elata* exert pathway-selective modulation in accordance with the prevailing pathophysiological context. In oxidative stress-dominant conditions, such as ischemic stroke and vascular dementia, the Nrf2-mediated antioxidant defense axis is preferentially engaged; in protein aggregation-driven disorders, including Alzheimer’s and Parkinson’s diseases, therapeutic priority is placed on intercepting the NF-κB/NLRP3 inflammatory axis; and in neuronal hyperexcitability-related conditions such as epilepsy, the emphasis shifts toward fine-tuning MAPK/CREB signaling equilibrium alongside GABAergic neurotransmission (An et al., 2003). This “context-adaptive pathway selectivity” may constitute the molecular foundation underpinning the pleiotropic therapeutic effects of *G. elata* across neurologically distinct disease entities.

A further layer of complexity emerges from the observation that the same signaling pathway responds differentially to *G. elata* constituents depending on the cell type in question—microglia, astrocytes, or neurons. This cell type-specificity is exemplified by the NF-κB pathway: gastrodin inhibits TLR4/TRAF6/NF-κB signaling to suppress M1 polarization in microglia (Wang et al., 2024), engages the STAT3 pathway to attenuate inflammasome activation in astrocytes (Sui et al., 2019), and acts through the ADRA1/NF-κB/NLRP3 axis to directly counteract Aβ42-induced injury in neurons (Li B. et al., 2025). A similar paradigm applies to Nrf2 activation, which is mediated by Akt/GSK3β in astrocytes but operates through a P62/Keap1-dependent mechanism in neurons (Lin et al., 2021). In the epileptic brain, the impact of gastrodin on GABA metabolism is particularly evident as reduced GABA-T immunoreactivity in hippocampal neurons (An et al., 2003). Collectively, these findings argue against a unitary “pathway switch” model of *G. elata* action, and instead support a paradigm in which the therapeutic effects arise from the concerted engagement of cell-selective signaling nodes. Advancing this field will require future research to systematically dissect cell type-specific pathway regulation, complementing conventional tissue-level analyses with single-cell resolution approaches.

### Core mechanism: the NF-κB/NLRP3 pathway as a common node of anti-inflammatory effects

*G. elata* and its active components exert anti-neuroinflammatory effects in multiple neurological disease models—including Parkinson’s disease, Alzheimer’s disease, acute ischemic stroke, vascular dementia, and epilepsy—by regulating the NF-κB and NLRP3 inflammasome pathways, suggesting that these two pathways constitute the core molecular basis for its “same treatment for different diseases” (Li S. et al., 2025; Shao et al., 2021; Wang et al., 2024). NF-κB is not only a key transcription factor driving M1 macrophage polarization but also regulates the expression of various pro-inflammatory genes, including TNF-α, IL-1β, IL-6, IL-12, and cyclooxygenase-2 (Wang et al., 2014). Moreover, it serves as the initiation signal for NLRP3 inflammasome activation by inducing the transcription of NLRP3 and pro-IL-1β in response to pathogen-associated molecular patterns (PRRs) and cytokines (Afonina et al., 2017). The regulation of the NF-κB pathway by *G. elata* occurs primarily through the classical TLR4-NF-κB axis, yet the intervention nodes differ among active components. *G. elata* extract indirectly inhibits NF-κB activation by suppressing the interaction between JNK and TLR4 (Huang et al., 2024), gastrodin directly downregulates the TRAF6 molecule within the pathway (Wang et al., 2024), and in AD models, gastrodin simultaneously blocks NF-κB activation and NLRP3 inflammasome over-assembly by inhibiting ADRA1 expression (Li B. et al., 2025). Notably, *G. elata* miRNAs (Gas-miR01, Gas-miR02) downregulate the expression of A20, an NF-κB negative-feedback regulatory protein (Priem et al., 2020). This suggests that certain components of *G. elata* may exhibit “bidirectional regulatory” characteristics: on one hand, they suppress excessive inflammation; on the other, by downregulating A20, they might potentially interfere with the resolution of inflammation — a possibility that warrants further investigation (Xia et al., 2020). It is important to emphasize that glial cell activation plays a protective role in neural repair. Excessive inhibition of astrocytic boundary formation can impede wound healing, exacerbate inflammatory spread, compromise blood–brain barrier (BBB) repair, and ultimately lead to poor prognosis (O’Shea et al., 2024). In current research, all tested components of *G. elata* demonstrate anti-inflammatory effects; nevertheless, their potential impact on the physiological functions of glial cells requires careful evaluation.

### Multi-pathway cross-regulation and cell model selection

In addition to the NF-κB/NLRP3 axis, components such as gastrodin also regulate neuroinflammation through other signaling pathways, including MAPK (JNK, p38 MAPK, PI3K-Akt), JAK2-STAT3, NOTCH-1, and RAS (Duan et al., 2023; Guo et al., 2021; Huang et al., 2024; Wang et al., 2018; Zhang et al., 2024a). Extensive crosstalk exists among these pathways. Among them, the NF-κB pathway serves as a central hub for inflammatory amplification, forming a complex regulatory network with JNK, PI3K-Akt, JAK2-STAT3, and NOTCH-1 (Guo et al., 2024). SH-SY5Y and PC12 cells are currently the most commonly used *in vitro* models in neurological disease research (Wiatrak et al., 2020; Xicoy et al., 2017), NOTCH can exert atypical biological functions by regulating the JAK/STAT, PI3K/AKT, and NF-κB pathways via NICD in lineage-restricted progenitor cells, fate-specific differentiation, and tumorigenesis (Shi Q. et al., 2024). However, whether NOTCH functions as an upstream unified regulatory node for the aforementioned pathways requires further experimental verification.

### Cross-mechanisms of antioxidant stress and cell death regulation

The antioxidant stress effects of *G. elata* are primarily reflected in increased activities of antioxidant enzymes such as SOD, CAT, and GSH, as well as inhibition of peroxidases including COX-2 and iNOS. This effect is associated with DAF-16 nuclear translocation and upregulation of SKN-1, SOD-3, and GST-4 expression (Yu et al., 2024a) Nrf2 is a key target of the antioxidative stress action of *G. elata* (Wang Y. et al., 2025; Zhang et al., 2024b; Zhou et al., 2025). The PI3K-Akt pathway promotes dissociation of the Keap1/Nrf2 complex, induces Nrf2 nuclear translocation, and activates downstream antioxidant enzymes such as HO-1, NQO-1, and GPx4 (Lu et al., 2016). *G. elata* inhibits ferroptosis-mediated neuroinflammation through the PI3K-Akt-Nrf2-GPX4/HO-1 pathway (Wang et al., 2026). Furthermore, components of *G. elata* reduce caspase-9/3-mediated mitochondrial apoptosis by regulating the Bcl-2/Bax ratio (Gan et al., 2024) and inhibiting mitochondrial cytochrome c release (Lai et al., 2022). In terms of neurotrophic support, *G. elata* promotes the release of neurotrophic factors (BDNF, GDNF) and growth-promoting factors (GAP43), thereby exerting neuroprotective effects (Ding et al., 2019; Duan et al., 2023). Additionally, it maintains blood–brain barrier (BBB) integrity by regulating the extracellular matrix and MMP expression (Luo et al., 2023; Wang L. et al., 2025).

### Remote regulation of the gut microbiota–gut–brain axis

Short-chain fatty acids (SCFAs, e.g., butyrate, propionate, and acetate) generated by gut microbial fermentation of dietary fiber inhibit microglial activation and reduce pro-inflammatory cytokine levels by stimulating free fatty acid receptors FFAR2/FFAR3 or by inhibiting histone deacetylases (HDACs) (de Vos et al., 2022; Krautkramer et al., 2021; Loh et al., 2024). *G. elata* polysaccharides, gastrodin, and fermented *G. elata* extract (FGGE) each regulate neuroinflammation via the “microbiota–gut–brain axis” (Fasina et al., 2022; Gan et al., 2024; Huang et al., 2025). In PD rats, *G. elata* polysaccharides increased colonic SCFA content and upregulated expression of the intestinal barrier protein occludin, thereby improving gut barrier function (Gan et al., 2024). *G. elata* is also effective in treating colitis (Zhang et al., 2025). However, it remains unclear whether *G. elata* components can directly cross the blood–brain barrier (BBB) to act on the central nervous system, and the relative contribution of peripheral versus central mechanisms to its overall neuroprotective effects has yet to be determined. At present, the available evidence for the gut microbiota–brain axis is predominantly associative in nature, and causal relationships have not been firmly established. Whether the observed changes in gut microbiota composition directly mediate the neuroprotective effects of *G. elata* or merely represent epiphenomena of the disease state remains to be determined. Current status and bottlenecks of clinical translation.

Clinical research on *G. elata* has mainly focused on compound preparations, particularly *G. elata*–Uncaria decoction. This formulation has demonstrated antihypertensive efficacy and improvement of related symptoms in patients with masked hypertension (Franklin et al., 2017; Zhang et al., 2020). Gastrodin entered clinical trials relatively early due to its well-established research foundation. The QZTL capsule, which contains gastrodin, improves lower-limb functional recovery in patients after ischemic stroke (Yu et al., 2021). Perioperative infusion of gastrodin significantly reduces the incidence of postoperative delirium in patients undergoing coronary artery bypass grafting (CABG) and improves discharge outcomes (Bai et al., 2025). The gastrodin-based nutritional health formula Assonal^®^ alleviates pain, improves function, and enhances quality of life in patients with radiculopathy and carpal tunnel syndrome (Invernizzi et al., 2026). Although gastrodin has shown favorable safety and preliminary efficacy in clinical trials, independent clinical studies on other active ingredients of *G. elata* — including parishin, p-hydroxybenzyl alcohol, and polysaccharides — remain seriously limited. Most existing trials are small-scale and lack high-quality randomized controlled evidence.

### Limitations of the review

Several methodological limitations of this review should be acknowledged. First, although we conducted a systematic literature search across five databases, the restriction to English- and Chinese-language publications may have introduced language bias, potentially excluding relevant studies published in other languages. Second, the substantial heterogeneity among the included preclinical studies—encompassing different disease models, animal species and strains, intervention protocols (dosage, route, and duration of administration), and outcome measures—precludes quantitative comparisons and limits the generalizability of the mechanistic conclusions. Third, the predominance of studies investigating gastrodin, relative to other *G. elata* constituents such as parishin and 4-HBA, reflects a significant evidence gap that should be interpreted as a limitation of the available literature rather than of this review per se. Fourth, the possibility of publication bias cannot be excluded, as negative or null findings are less likely to be published, which may result in an overestimation of the therapeutic effects.

An additional limitation concerns the methodological quality of the primary evidence base. As shown in the SYRCLE risk-of-bias assessment (Figures 4, 5), the vast majority of the included animal studies were judged at unclear risk of bias (97.7%), with only one study rated as high risk and none meeting the prespecified criteria for overall low risk. The most substantial reporting gaps were observed in items pertaining to selection bias (sequence generation, baseline characteristics, allocation concealment) and performance bias (random housing, blinding of caregivers/investigators), all of which were judged as unclear in 100% of the studies. Detection bias (blinding of outcome assessors) and attrition bias (incomplete outcome data) were also predominantly unclear. These findings should not be automatically interpreted as evidence that the experiments were conducted poorly; rather, they reflect a widespread failure to report the methodological safeguards required to judge study conduct. Nevertheless, the missing items are not peripheral details—randomization, allocation concealment, and blinding are central protections against systematic error in animal experiments. When these procedures are not reported, the direction and magnitude of potential bias cannot be determined reliably, and treatment effects may be overestimated. Only one study was judged at overall high risk, as it explicitly stated that the authors were aware of group allocation, experimental conduct, and outcome assessment, and a prespecified endpoint was omitted from the results. Notably, this study contributes only a small fraction of evidence to the present synthesis, so its high risk of bias is unlikely to materially affect the overall conclusions.

**FIGURE 4:**
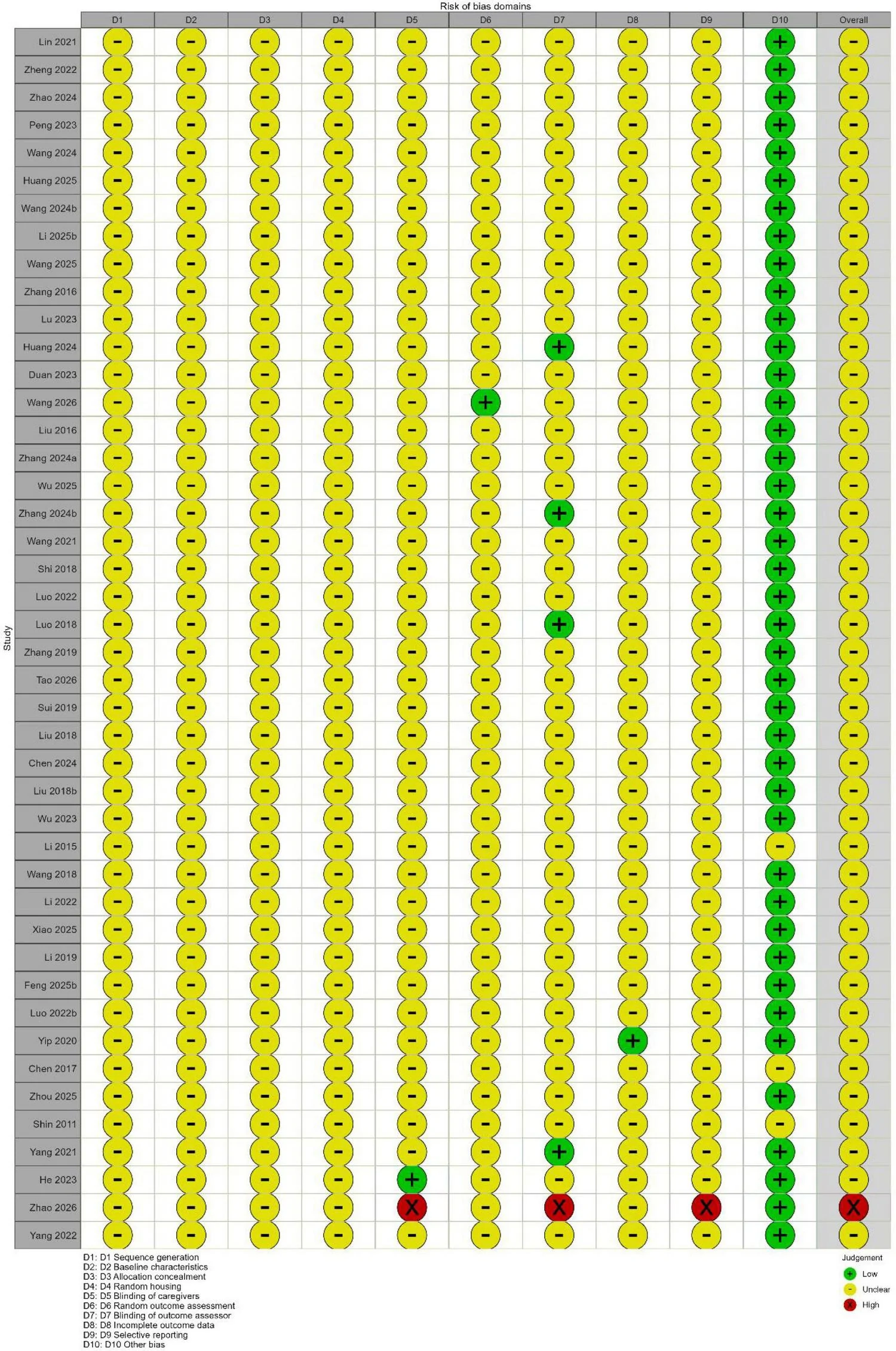
Risk of bias of the included animal studies: traffic light plot. Study-level judgements for the 10 SYRCLE domains (D1–D10) and the overall judgment for each of the 44 included studies. Green, low risk; yellow, unclear risk; red, high risk.

**FIGURE 5:**
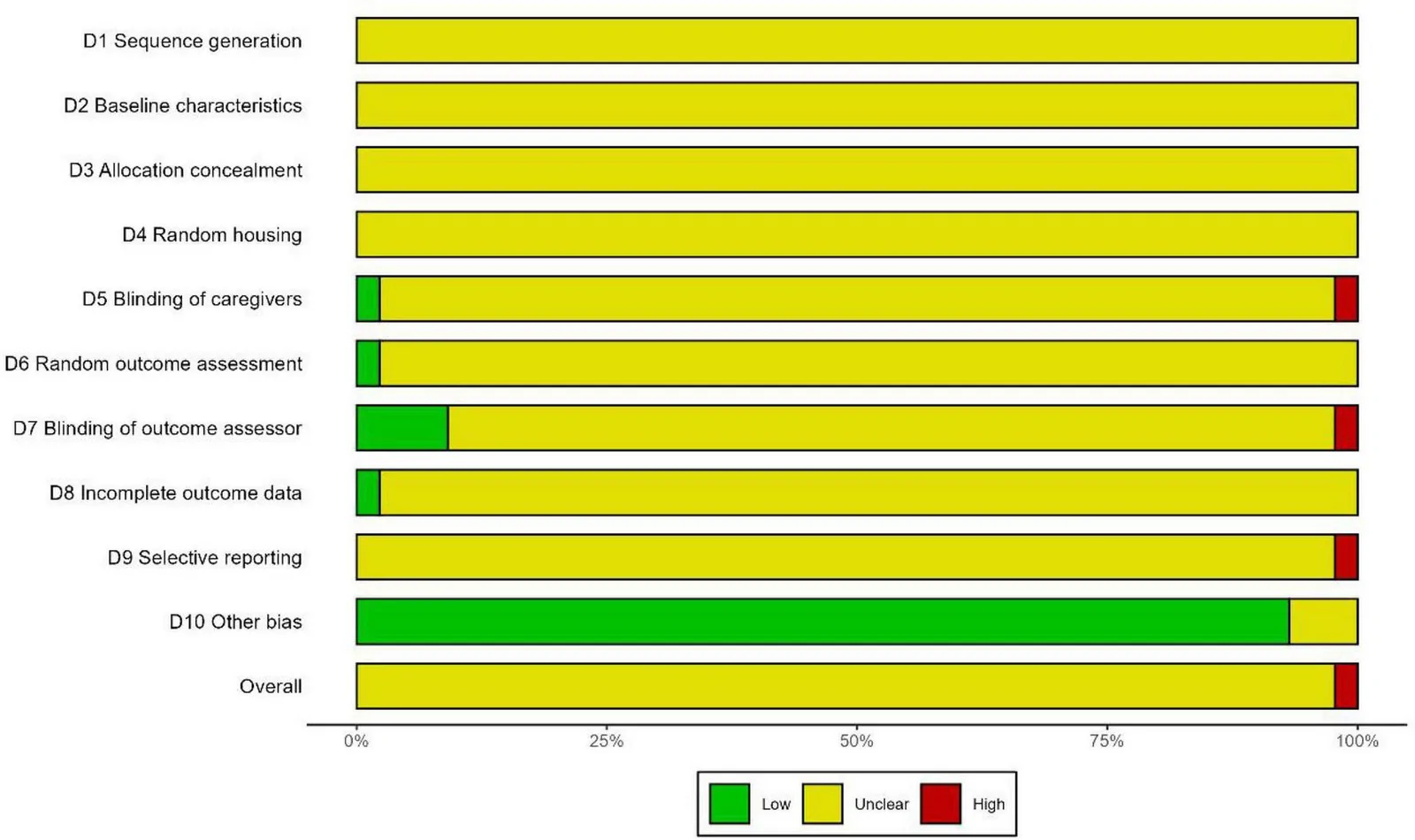
Risk of bias of the included animal studies: summary bar plot. Proportion of studies judged as low, unclear, or high risk of bias for each SYRCLE domain and overall.

In light of these limitations, the conclusions of this review should be interpreted with appropriate caution. The consistency and biological plausibility of effects across multiple independent studies provide some confidence in the anti-inflammatory potential of *G. elata* and its constituents, yet incomplete reporting limits confidence in the precision and internal validity of the underlying animal experiments. Domain-level results should be emphasized rather than relying solely on the summary judgement, because the overall category conceals substantial variation across individual domains. To improve the reproducibility and interpretability of future research, investigators should adhere to the ARRIVE 2.0 reporting guidelines (Percie du Sert et al., 2020). At a minimum, authors should report the method used to generate the allocation sequence, whether allocation was concealed, whether animals were randomly housed, who was blinded and at which stage, how animals were selected for each outcome, the number of animals excluded or lost with reasons, and whether analyses followed a prespecified protocol. Prospective registration of animal study protocols, sample-size justification, complete animal flow diagrams, and access to study-level data would further strengthen the evidence base. These limitations should be taken into consideration when interpreting the findings of this review, and they underscore the need for more standardized, well-powered, and transparently reported preclinical studies in the future.

### Dual roles of neuroinflammation and future directions

Neuroinflammation plays both protective and detrimental roles in disease progression. Microglial activation in multiple sclerosis is predominantly harmful (das Neves et al., 2021), whereas in Alzheimer‘s disease and Parkinson’s disease it exhibits biphasic characteristics: early moderate activation facilitates clearance of misfolded proteins, but persistent stimulation leads to aberrant microglial function (Leng and Edison, 2021). Therefore, an ideal anti-inflammatory strategy should selectively suppress the neurotoxicity and pro-inflammatory activity of microglia while preserving their phagocytic capacity to clear pathogenic proteins (Shi and Yong, 2025). *G. elata* has been shown to remove α-synuclein and Aβ plaques (Peng et al., 2023; Wang et al., 2024), exhibits anti-inflammatory and neuroprotective properties, and synergistically alleviates central inflammation by maintaining blood–brain barrier (BBB) integrity (Li B. et al., 2025). The bidirectional regulatory potential of gastrodin on microglial activation warrants further investigation.

Moreover, *in vivo* distribution studies of active ingredients from *G. elata* suggest that after BBB restoration during the later stages of disease recovery, intracerebral drug exposure may significantly decline (Shao et al., 2025). How to maintain effective central concentrations remains an urgent pharmacokinetic challenge in this field.

## Conclusion

*G. elata* and its active ingredients have demonstrated extensive and well-defined neuroprotective effects in animal models of Parkinson’s disease, Alzheimer’s disease, acute ischemic stroke, vascular dementia, and epilepsy through a multi-component, multi-target, multi-pathway systemic anti-inflammatory mechanism. The core of their action lies in the precise regulation of the neuroinflammatory microenvironment: dynamically balancing the activities of the Nrf2/HO-1 antioxidant pathway and the NF-κB/NLRP3 inflammasome pathway, thereby enhancing endogenous antioxidant capacity while inhibiting the release of pro-inflammatory factors such as IL-1β, TNF-α, and IL-6. They also coordinately regulate microglial M1/M2 polarization, inhibit ferroptosis, pyroptosis, and apoptosis, promote the expression of antioxidant enzymes, and remodel beneficial gut microbiota and repair the intestinal barrier via the gut microbiota–gut–brain axis, thus breaking the vicious cycle of inflammation and neuronal damage at multiple nodes.

Although current research faces challenges such as uneven distribution of evidence for active ingredients, insufficient causal validation of mechanisms, and pharmacokinetic bottlenecks, the following strategies are expected to facilitate the clinical translation of *G. elata*: deepening conditional gene knockout validation to clarify pathway necessity; expanding mechanistic and clinical studies on non-gastrodin active ingredients (e.g., parishin, p-hydroxybenzyl alcohol, and polysaccharides); developing brain-targeted drug delivery systems or intranasal formulations; and promoting multicenter, randomized, double-blind clinical trials. *G. elata* holds promise for evolving from a traditional Chinese medicine into a precise and effective therapeutic agent for neurological diseases, offering a “same treatment for different diseases” strategy for various brain disorders that share neuroinflammation as a common pathological basis.

## Data Availability

The original contributions presented in this study are included in this article/Supplementary material, further inquiries can be directed to the corresponding authors.

## References

[B1] Achariyar T. M. Li B. Peng W. Verghese P. B. Shi Y. McConnell E. et al. (2016). Glymphatic distribution of CSF-derived apoE into brain is isoform specific and suppressed during sleep deprivation. *Mol. Neurodegener.* 11:74. 10.1186/s13024-016-0138-8 27931262 PMC5146863

[B2] Afonina I. S. Zhong Z. Karin M. Beyaert R. (2017). Limiting inflammation-the negative regulation of NF-κB and the NLRP3 inflammasome. *Nat. Immunol.* 18, 861–869. 10.1038/ni.3772 28722711

[B3] An S. J. Park S. K. Hwang I. K. Choi S. Y. Kim S. K. Kwon O. S. et al. (2003). Gastrodin decreases immunoreactivities of gamma-aminobutyric acid shunt enzymes in the hippocampus of seizure-sensitive gerbils. *J. Neurosci. Res.* 71, 534–543. 10.1002/jnr.10502 12548709

[B4] Anrather J. Iadecola C. (2016). Inflammation and stroke: An overview. *Neurotherapeutics* 13, 661–670. 10.1007/s13311-016-0483-x 27730544 PMC5081118

[B5] Arvidsson A. Collin T. Kirik D. Kokaia Z. Lindvall O. (2002). Neuronal replacement from endogenous precursors in the adult brain after stroke. *Nat. Med.* 8, 963–970. 10.1038/nm747 12161747

[B6] Bai Y. X. Wu H. L. Xie W. L. Li X. Han J. J. Liu J. et al. (2025). Efficacy and safety of gastrodin in preventing postoperative delirium following cardiac surgery: A randomized placebo controlled clinical trial. *Crit. Care.* 29:108. 10.1186/s13054-025-05331-9 40069830 PMC11895371

[B7] Bhandage A. K. Barragan A. (2021). GABAergic signaling by cells of the immune system: More the rule than the exception. *Cell. Mol. Life Sci.* 78, 5667–5679. 10.1007/s00018-021-03881-z 34152447 PMC8316187

[B8] Cai A. Li Z. Liu R. Huang H. Liu P. Zhang R. et al. (2026). Gastrodin alleviates neuronal damage in epileptic cell models by targeting P2RY12 to inhibit microglial hyperactivation. *PLoS One* 21:e0346877. 10.1371/journal.pone.0346877 41996416 PMC13089871

[B9] Cai Z. Huang J. Luo H. Lei X. Yang Z. Mai Y. et al. (2013). Role of glucose transporters in the intestinal absorption of gastrodin, a highly water-soluble drug with good oral bioavailability. *J. Drug. Target.* 21, 574–580. 10.3109/1061186x.2013.778263 23480725

[B10] Caraci F. Spampinato S. Sortino M. A. Bosco P. Battaglia G. Bruno V. et al. (2012). Dysfunction of TGF-β1 signaling in Alzheimer’s disease: Perspectives for neuroprotection. *Cell. Tissue. Res.* 347, 291–301. 10.1007/s00441-011-1230-6 21879289

[B11] Chen L. Liu X. Wang H. Qu M. (2017). Gastrodin attenuates pentylenetetrazole-induced seizures by modulating the mitogen-activated protein kinase-associated inflammatory responses in mice. *Neurosci. Bull.* 33, 264–272. 10.1007/s12264-016-0084-z 27909971 PMC5567506

[B12] Chen Y. X. Yang H. Wang D. S. Chen T. T. Qi X. L. Tao L. et al. (2024). Gastrodin alleviates mitochondrial dysfunction by regulating SIRT3-mediated TFAM acetylation in vascular dementia. *Phytomedicine* 128, 155369. 10.1016/j.phymed.2024.155369 38547618

[B13] Choi J. H. Lee D. U. (2006). A new citryl glycoside from gastrodia elata and its inhibitory activity on GABA transaminase. *Chem. Pharm. Bull.* 54, 1720–1721. 10.1248/cpb.54.1720 17139109

[B14] Colton C. Wilcock D. M. (2010). Assessing activation states in microglia. *CNS Neurol. Disord. Drug. Targets.* 9, 174–191. 10.2174/187152710791012053 20205642

[B15] Dai Y. Ban W. Yang Z. (2024). Gastrodin, a promising natural small molecule for the treatment of central nervous system disorders, and its recent progress in synthesis, pharmacology and pharmacokinetics. *Int. J. Mol. Sci.* 25:9540. 10.3390/ijms25179540 39273485 PMC11394983

[B16] das Neves S. P. Sousa J. C. Sousa N. Cerqueira J. J. Marques F. (2021). Altered astrocytic function in experimental neuroinflammation and multiple sclerosis. *Glia* 69, 1341–1368. 10.1002/glia.23940 33247866

[B17] De Meyer S. F. Denorme F. Langhauser F. Geuss E. Fluri F. Kleinschnitz C. (2016). Thromboinflammation in stroke brain damage. *Stroke* 47, 1165–1172. 10.1161/strokeaha.115.011238 26786115

[B18] de Vos W. M. Tilg H. Van Hul M. Cani P. D. (2022). Gut microbiome and health: Mechanistic insights. *Gut* 71, 1020–1032. 10.1136/gutjnl-2021-326789 35105664 PMC8995832

[B19] Ding Y. Bao X. Lao L. Ling Y. Wang Q. Xu S. (2019). P-hydroxybenzyl alcohol prevents memory deficits by increasing neurotrophic factors and decreasing inflammatory factors in a mice model of Alzheimer’s disease. *J. Alzheimers Dis.* 67, 1007–1019. 10.3233/jad-180910 30776009

[B20] Duan X. Song N. Ma K. Tong Y. Yang L. (2023). The effects of protein-rich extract from rhizoma gastrodiae against cerebral ischemia/reperfusion injury via regulating MAPK and PI3K/AKT signaling pathway. *Brain. Res. Bull.* 203:110772. 10.1016/j.brainresbull.2023.110772 37793596

[B21] Esiri M. Chance S. Joachim C. Warden D. Smallwood A. Sloan C. et al. (2015). Cerebral amyloid angiopathy, subcortical white matter disease and dementia: Literature review and study in OPTIMA. *Brain Pathol.* 25, 51–62. 10.1111/bpa.12221 25521177 PMC8028928

[B22] Fasina O. B. Wang J. Mo J. Osada H. Ohno H. Pan W. et al. (2022). Gastrodin from gastrodia elata enhances cognitive function and neuroprotection of AD mice via the regulation of gut microbiota composition and inhibition of neuron inflammation. *Front. Pharmacol.* 13:814271. 10.3389/fphar.2022.814271 35721206 PMC9201506

[B23] Feng J. Yang Q. Chen M. Ning L. Wang Y. Luo D. et al. (2025a). Study of the correlation between the anti-ischemic stroke mechanism of 4-hydroxybenzaldehyde and its response to reactive oxygen species in brain metabolism. *J. Pharmacol. Exp. Ther.* 392:103395. 10.1016/j.jpet.2025.103395 40080989

[B24] Feng J. Yang Q. Chen M. Wang Y. Luo D. Hu D. et al. (2025b). Protective effects of 4-HBd on blood-brain barrier integrity in MCAO/R model rats based on brain pharmacokinetic characteristics. *Front. Pharmacol.* 16:1528839. 10.3389/fphar.2025.1528839 40264675 PMC12012380

[B25] Franceschi C. Valensin S. Fagnoni F. Barbi C. Bonafè M. (1999). Biomarkers of immunosenescence within an evolutionary perspective: The challenge of heterogeneity and the role of antigenic load. *Exp. Gerontol.* 34, 911–921. 10.1016/s0531-5565(99)00068-6 10673145

[B26] Franklin S. S. O’Brien E. Staessen J. A. (2017). Masked hypertension: Understanding its complexity. *Eur. Heart J.* 38, 1112–1118. 10.1093/eurheartj/ehw502 27836914

[B27] Gan Q. X. Peng M. Y. Wei H. B. Chen L. L. Chen X. Y. Li Z. H. et al. (2024). Gastrodia elata polysaccharide alleviates Parkinson’s disease via inhibiting apoptotic and inflammatory signaling pathways and modulating the gut microbiota. *Food. Funct.* 15, 2920–2938. 10.1039/d3fo05169b 38385354

[B28] GBD 2019 Meningitis Antimicrobial Resistance Collaborators. (2023). Global, regional, and national burden of meningitis and its aetiologies, 1990-2019: A systematic analysis for the global burden of disease study 2019. *Lancet Neurol.* 22, 685–711. 10.1016/s1474-4422(23)00195-3 37479374 PMC10356620

[B29] GBD Epilepsy Collaborators. (2025). Global, regional, and national burden of epilepsy, 1990-2021: A systematic analysis for the global burden of disease study 2021. *Lancet Public Health* 10, e203–e227. 10.1016/s2468-2667(24)00302-5 40015291 PMC11876103

[B30] Ghosh S. Wu M. D. Shaftel S. S. Kyrkanides S. LaFerla F. M. Olschowka J. A. et al. (2013). Sustained interleukin-1β overexpression exacerbates tau pathology despite reduced amyloid burden in an Alzheimer’s mouse model. *J. Neurosci.* 33, 5053–5064. 10.1523/jneurosci.4361-12.2013 23486975 PMC3637949

[B31] Glass C. K. Saijo K. Winner B. Marchetto M. C. Gage F. H. (2010). Mechanisms underlying inflammation in neurodegeneration. *Cell* 140, 918–934. 10.1016/j.cell.2010.02.016 20303880 PMC2873093

[B32] Gordon D. Dafinca R. Scaber J. Alegre-Abarrategui J. Farrimond L. Scott C. et al. (2019). Single-copy expression of an amyotrophic lateral sclerosis-linked TDP-43 mutation (M337V) in BAC transgenic mice leads to altered stress granule dynamics and progressive motor dysfunction. *Neurobiol. Dis.* 121, 148–162. 10.1016/j.nbd.2018.09.024 30290270

[B33] Govindarajan V. de Rivero Vaccari J. P. Keane R. W. (2020). Role of inflammasomes in multiple sclerosis and their potential as therapeutic targets. *J. Neuroinflammation* 17:260. 10.1186/s12974-020-01944-9 32878648 PMC7469327

[B34] Guo H. Cheng D. Zhang C. Xue F. (2025). The pharmacological effects of gastrodin and the progress in the treatment of neurological disorders. *Am. J. Chin. Med.* 53, 803–831. 10.1142/s0192415x25500302 40374368

[B35] Guo J. Zhang X. L. Bao Z. R. Yang X. K. Li L. S. Zi Y. et al. (2021). Gastrodin regulates the notch signaling pathway and sirt3 in activated microglia in cerebral hypoxic-ischemia neonatal rats and in activated BV-2 microglia. *Neuromolecular. Med.* 23, 348–362. 10.1007/s12017-020-08627-x 33095377

[B36] Guo Q. Jin Y. Chen X. Ye X. Shen X. Lin M. et al. (2024). NF-κB in biology and targeted therapy: New insights and translational implications. *Signal Transduct Target Ther.* 9:53. 10.1038/s41392-024-01757-9 38433280 PMC10910037

[B37] Ha J. H. Shin S. M. Lee S. K. Kim J. S. Shin U. S. Huh K. et al. (2001). In vitro effects of hydroxybenzaldehydes from Gastrodia elata and their analogues on GABAergic neurotransmission, and a structure-activity correlation. *Planta. Med.* 67, 877–880. 10.1055/s-2001-18844 11745032

[B38] Ha S. K. Moon E. Lee P. Ryu J. H. Oh M. S. Kim S. Y. (2012). Acacetin attenuates neuroinflammation via regulation the response to LPS stimuli in vitro and in vivo. *Neurochem. Res.* 37, 1560–1567. 10.1007/s11064-012-0751-z 22447574

[B39] Han Y. J. Je J. H. Kim S. H. Ahn S. M. Kim H. N. Kim Y. R. et al. (2014). Gastrodia elata shows neuroprotective effects via activation of PI3K signaling against oxidative glutamate toxicity in HT22 cells. *Am. J. Chin. Med.* 42, 1007–1019. 10.1142/s0192415x14500633 25004888

[B40] Hastings T. G. Lewis D. A. Zigmond M. J. (1996). Role of oxidation in the neurotoxic effects of intrastriatal dopamine injections. *Proc. Natl. Acad. Sci. U. S. A.* 93, 1956–1961. 10.1073/pnas.93.5.1956 8700866 PMC39890

[B41] He J. Li X. Yang S. Li Y. Lin X. Xiu M. et al. (2021). Gastrodin extends the lifespan and protects against neurodegeneration in the drosophila PINK1 model of Parkinson’s disease. *Food Funct.* 12, 7816–7824. 10.1039/d1fo00847a 34232246

[B42] He X. Yang Y. Yuan X. Sun Y. Li Y. (2023). Chemical composition and anticonvulsant activities of herb pair of gastrodia elata blume-acorus tatarinowii schott decoction on experimentally induced seizures in mice. *Metab. Brain Dis.* 38, 1877–1893. 10.1007/s11011-023-01211-9 37043151

[B43] Heneka M. T. McManus R. M. Latz E. (2018). Inflammasome signalling in brain function and neurodegenerative disease. *Nat. Rev. Neurosci.* 19, 610–621. 10.1038/s41583-018-0055-7 30206330

[B44] Hickman S. E. Allison E. K. El Khoury J. (2008). Microglial dysfunction and defective beta-amyloid clearance pathways in aging Alzheimer’s disease mice. *J. Neurosci.* 28, 8354–8360. 10.1523/jneurosci.0616-08.2008 18701698 PMC2597474

[B45] Hooijmans C. R. Rovers M. M. de Vries R. B. Leenaars M. Ritskes-Hoitinga M. Langendam M. W. S. Y. R. C. L. E. (2014). ’s risk of bias tool for animal studies. *BMC Med. Res. Methodol.* 14:43. 10.1186/1471-2288-14-43 24667063 PMC4230647

[B46] Hort J. Vališ M. Kuča K. Angelucci F. (2019). Vascular cognitive impairment: Information from animal models on the pathogenic mechanisms of cognitive deficits. *Int. J. Mol. Sci.* 20:2405. 10.3390/ijms20102405 31096580 PMC6566630

[B47] Hsieh C. L. Tang N. Y. Chiang S. Y. Hsieh C. T. Lin J. G. (1999). Anticonvulsive and free radical scavenging actions of two herbs, uncaria rhynchophylla (MIQ) Jack and Gastrodia elata Bl., in kainic acid-treated rats. *Life Sci.* 65, 2071–2082. 10.1016/s0024-3205(99)00473-7 10579461

[B48] Huang B. Lin Z. Chen Z. Chen J. Shi B. Jia J. et al. (2023). Strain differences in the drug transport capacity of intestinal glucose transporters in sprague-dawley versus wistar rats, C57BL/6J versus kunming mice. *Int. J. Pharm.* 640:123000. 10.1016/j.ijpharm.2023.123000 37254285

[B49] Huang J. Lai L. Su Y. Chen J. Li P. Du B. (2025). Probiotic-fermented ginger-processed Gastrodia elata BI. Ameliorates AlCl(3)-induced cognitive dysfunction in an Alzheimer’s disease rat model by regulating the gut microbiota and CREB/BDNF pathway. *Food. Res. Int.* 207:116087. 10.1016/j.foodres.2025.116087 40086974

[B50] Huang S. C. Huang H. C. Liao W. L. Kao S. T. Cheng C. Y. (2024). Neuroprotective effects of Gastrodia elata Blume on promoting M2 microglial polarization by inhibiting JNK/TLR4/T3JAM/NF-κB signaling after transient ischemic stroke in rats. *Front. Pharmacol.* 15:1469602. 10.3389/fphar.2024.1469602 39391701 PMC11465390

[B51] Iadecola C. Anrather J. (2011). The immunology of stroke: From mechanisms to translation. *Nat. Med.* 17, 796–808. 10.1038/nm.2399 21738161 PMC3137275

[B52] Invernizzi M. Lippi L. Mulè S. Galla R. Folli A. Tiso D. et al. (2026). Real-world before-after study of gastrodia elata blume-based nutraceutical supplementation in patients with radiculopathy and carpal tunnel syndrome. *Nutrients* 18:1438. 10.3390/nu18091438 42124037 PMC13165183

[B53] Ionescu-Tucker A. Cotman C. W. (2021). Emerging roles of oxidative stress in brain aging and Alzheimer’s disease. *Neurobiol. Aging.* 107, 86–95. 10.1016/j.neurobiolaging.2021.07.014 34416493

[B54] Ising C. Venegas C. Zhang S. Scheiblich H. Schmidt S. V. Vieira-Saecker A. et al. (2019). NLRP3 inflammasome activation drives tau pathology. *Nature* 575, 669–673. 10.1038/s41586-019-1769-z 31748742 PMC7324015

[B55] Jang J. H. Son Y. Kang S. S. Bae C. S. Kim J. C. Kim S. H. et al. (2015). Neuropharmacological potential of gastrodia elata blume and its components. *Evid. Based. Complement. Alternat. Med.* 2015:309261. 10.1155/2015/309261 26543487 PMC4620291

[B56] Jayaraj R. L. Azimullah S. Beiram R. Jalal F. Y. Rosenberg G. A. (2019). Neuroinflammation: Friend and foe for ischemic stroke. *J. Neuroinflammation* 16:142. 10.1186/s12974-019-1516-2 31291966 PMC6617684

[B57] Jiang G. Hu Y. Liu L. Cai J. Peng C. Li Q. (2014). Gastrodin protects against MPP(+)-induced oxidative stress by up regulates heme oxygenase-1 expression through p38 MAPK/Nrf2 pathway in human dopaminergic cells. *Neurochem. Int.* 75, 79–88. 10.1016/j.neuint.2014.06.003 24932697

[B58] Jiang T. Cheng H. Su J. Wang X. Wang Q. Chu J. et al. (2020). Gastrodin protects against glutamate-induced ferroptosis in HT-22 cells through Nrf2/HO-1 signaling pathway. *Toxicol. In. Vitro.* 62:104715. 10.1016/j.tiv.2019.104715 31698019

[B59] Jin K. Wang X. Xie L. Mao X. O. Zhu W. Wang Y. et al. (2006). Evidence for stroke-induced neurogenesis in the human brain. *Proc. Natl. Acad. Sci. U. S. A.* 103, 13198–13202. 10.1073/pnas.0603512103 16924107 PMC1559776

[B60] Ju X. H. Shi Y. Liu N. Guo D. M. Cui X. (2010). Determination and pharmacokinetics of gastrodin in human plasma by HPLC coupled with photodiode array detector. *J. Chromatogr. B Analyt. Technol. Biomed. Life Sci.* 878, 1982–1986. 10.1016/j.jchromb.2010.05.034 20542476

[B61] Krautkramer K. A. Fan J. Bäckhed F. (2021). Gut microbial metabolites as multi-kingdom intermediates. *Nat. Rev. Microbiol.* 19, 77–94. 10.1038/s41579-020-0438-4 32968241

[B62] Lai M. C. Liu W. Y. Liou S. S. Liu I. M. (2022). p-Hydroxybenzyl alcohol antagonized the ROS-dependent JNK/Jun/caspase-3 pathway to produce neuroprotection in a cellular model of Parkinson’s disease. *Nutrients* 14:5002. 10.3390/nu14235002 36501032 PMC9741417

[B63] Lee Y. S. Ha J. H. Yong C. S. Lee D. U. Huh K. Kang Y. S. et al. (1999). Inhibitory effects of constituents of gastrodia elata Bl. On glutamate-induced apoptosis in IMR-32 human neuroblastoma cells. *Arch. Pharm. Res.* 22, 404–409. 10.1007/bf02979066 10489882

[B64] Leng F. Edison P. (2021). Neuroinflammation and microglial activation in Alzheimer disease: Where do we go from here? *Nat. Rev. Neurol.* 17, 157–172. 10.1038/s41582-020-00435-y 33318676

[B65] Li B. Wang L. Xiao Y. Wang Y. Wang Y. Peng Y. et al. (2025). Gastrodin ameliorates tau pathology and BBB dysfunction in 3xTg-AD transgenic mice by regulating the ADRA1/NF-κB/NLRP3 pathway to reduce neuroinflammation. *Phytother. Res.* 39, 1996–2016. 10.1002/ptr.8461 39963073

[B66] Li H. Wu X. Wu G. Guo J. Nie Z. Sheng Q. (2026). Gastrodin alleviates tau hyperphosphorylation associated with AKT/GSK-3β signaling changes in an Alzheimer’s disease cell model. *J. Vis. Exp.* 10.3791/70432 41973724

[B67] Li Q. Cao Y. Dang C. Han B. Han R. Ma H. et al. (2020). Inhibition of double-strand DNA-sensing cGAS ameliorates brain injury after ischemic stroke. *EMBO Mol. Med.* 12:e11002. 10.15252/emmm.201911002 32239625 PMC7136961

[B68] Li S. Bian L. Fu X. Ai Q. Sui Y. Zhang A. et al. (2019). Gastrodin pretreatment alleviates rat brain injury caused by cerebral ischemic-reperfusion. *Brain Res.* 1712, 207–216. 10.1016/j.brainres.2019.02.006 30742808

[B69] Li S. Li M. Li Y. Liang S. Ling L. Wang H. et al. (2025). Gastrodia elata polysaccharide ameliorates Parkinson’s disease by enhancing dopamine levels, inhibiting NLRP3 inflammasome activation, and promoting mitochondrial autophagy. *Am. J. Chin. Med.* 53, 1813–1844. 10.1142/s0192415x25500673 40796532

[B70] Li Y. Zhang Z. (2015). Gastrodin improves cognitive dysfunction and decreases oxidative stress in vascular dementia rats induced by chronic ischemia. *Int. J. Clin. Exp. Pathol.* 8, 14099–14109. PMC4713509 26823723

[B71] Li Y. Zhang E. Yang H. Chen Y. Tao L. Xu Y. et al. (2022). Gastrodin ameliorates cognitive dysfunction in vascular dementia rats by suppressing ferroptosis via the regulation of the Nrf2/Keap1-GPx4 signaling pathway. *Molecules* 27:6311. 10.3390/molecules27196311 36234847 PMC9571513

[B72] Lin H. Xie L. Lv L. Chen J. Feng F. Liu W. et al. (2023). Intranasally administered thermosensitive gel for brain-targeted delivery of rhynchophylline to treat Parkinson’s disease. *Colloids Surf. B Biointerfaces.* 222:113065. 10.1016/j.colsurfb.2022.113065 36473372

[B73] Lin L. C. Chen Y. F. Lee W. C. Wu Y. T. Tsai T. H. (2008). Pharmacokinetics of gastrodin and its metabolite p-hydroxybenzyl alcohol in rat blood, brain and bile by microdialysis coupled to LC-MS/MS. *J. Pharm. Biomed. Anal.* 48, 909–917. 10.1016/j.jpba.2008.07.013 18757149

[B74] Lin L. C. Chen Y. F. Tsai T. R. Tsai T. H. (2007). Analysis of brain distribution and biliary excretion of a nutrient supplement, gastrodin, in rat. *Anal. Chim. Acta.* 590, 173–179. 10.1016/j.aca.2007.03.035 17448342

[B75] Lin Y. E. Lin C. H. Ho E. P. Ke Y. C. Petridi S. Elliott C. J. et al. (2021). Glial Nrf2 signaling mediates the neuroprotection exerted by gastrodia elata Blume in Lrrk2-G2019S Parkinson’s disease. *Elife* 10:e73753. 10.7554/eLife.73753 34779396 PMC8660019

[B76] Lindqvist D. Kaufman E. Brundin L. Hall S. Surova Y. Hansson O. (2012). Non-motor symptoms in patients with Parkinson’s disease - correlations with inflammatory cytokines in serum. *PLoS One* 7:e47387. 10.1371/journal.pone.0047387 23082161 PMC3474801

[B77] Liu B. Gao J. M. Li F. Gong Q. H. Shi J. S. (2018c). Gastrodin attenuates bilateral common carotid artery occlusion-induced cognitive deficits via regulating Aβ-Related proteins and reducing autophagy and apoptosis in rats. *Front. Pharmacol.* 9:405. 10.3389/fphar.2018.00405 29755351 PMC5932202

[B78] Liu B. Li F. Shi J. Yang D. Deng Y. Gong Q. (2016). Gastrodin ameliorates subacute phase cerebral ischemia-reperfusion injury by inhibiting inflammation and apoptosis in rats. *Mol. Med. Rep.* 14, 4144–4152. 10.3892/mmr.2016.5785 27748849 PMC5101922

[B80] Liu Y. Gao J. Peng M. Meng H. Ma H. Cai P. et al. (2018a). A review on central nervous system effects of gastrodin. *Front. Pharmacol.* 9:24. 10.3389/fphar.2018.00024 29456504 PMC5801292

[B79] Liu S. J. Liu X. Y. Li J. H. Guo J. Li F. Gui Y. et al. (2018b). Gastrodin attenuates microglia activation through renin-angiotensin system and Sirtuin3 pathway. *Neurochem. Int.* 120, 49–63. 10.1016/j.neuint.2018.07.012 30075231

[B81] Loh J. S. Mak W. Q. Tan L. K. S. Ng C. X. Chan H. H. Yeow S. H. et al. (2024). Microbiota-gut-brain axis and its therapeutic applications in neurodegenerative diseases. *Signal Transduct Target Ther.* 9:37. 10.1038/s41392-024-01743-1 38360862 PMC10869798

[B82] Lu G. W. Zou Y. J. Mo Q. Z. (1985). [Kinetic aspects of absorption, distribution, metabolism and excretion of 3H-gastrodin in rats]. *Yao. Xue. Xue. Bao.* 20, 167–172. 4072684

[B83] Lu M. C. Ji J. A. Jiang Z. Y. You Q. D. (2016). The Keap1-Nrf2-ARE pathway as a potential preventive and therapeutic target: An update. *Med. Res. Rev.* 36, 924–963. 10.1002/med.21396 27192495

[B84] Lu Z. Fu J. Wu G. Yang Z. Wu X. Wang D. et al. (2023). Neuroprotection and mechanism of gas-mir36-5p from gastrodia elata in an Alzheimer’s disease model by regulating glycogen synthase kinase-3β. *Int. J. Mol. Sci.* 24:17295. 10.3390/ijms242417295 38139125 PMC10744203

[B85] Luo K. Wang Y. Chen W. S. Feng X. Liao Y. Chen S. et al. (2022). Treatment combining focused ultrasound with gastrodin alleviates memory deficit and neuropathology in an Alzheimer’s disease-like experimental mouse model. *Neural. Plast.* 2022, 5241449. 10.1155/2022/5241449 35069727 PMC8776436

[B86] Luo L. Kim S. W. Lee H. K. Kim I. D. Lee H. Lee J. K. (2018a). Gastrodin exerts robust neuroprotection in the postischemic brain via its protective effect against Zn(2+)-toxicity and its anti-oxidative effects in astrocytes. *Anim. Cells. Syst.* 22, 429–437. 10.1080/19768354.2018.1549099 30533266 PMC6282451

[B87] Luo L. Kim S. W. Lee H. K. Kim I. D. Lee H. Lee J. K. (2018b). Anti-Zn(2+)-toxicity of 4-hydroxybenzyl alcohol in astrocytes and neurons contribute to a robust neuroprotective effects in the postischemic brain. *Cell. Mol. Neurobiol.* 38, 615–626. 10.1007/s10571-017-0508-y 28608001 PMC11481900

[B88] Luo S. Lin H. Wu C. Zhu L. Hua Q. Weng Y. et al. (2024). Cholinergic macrophages promote the resolution of peritoneal inflammation. *Proc. Natl. Acad. Sci. U. S. A.* 121:e2402143121. 10.1073/pnas.2402143121 38923993 PMC11228479

[B89] Luo Y. Chen P. Yang L. Duan X. (2023). Metabolomic analysis and pharmacological validation of the cerebral protective effect of 3,4-dihydroxybenzaldehyde on cerebral ischemia-reperfusion injury. *Mol. Med. Rep.* 27:9. 10.3892/mmr.2022.12896 36367170 PMC9685269

[B90] Lyman M. Lloyd D. G. Ji X. Vizcaychipi M. P. Ma D. (2014). Neuroinflammation: The role and consequences. *Neurosci. Res.* 79, 1–12. 10.1016/j.neures.2013.10.004 24144733

[B91] Marchi S. Guilbaud E. Tait S. W. G. Yamazaki T. Galluzzi L. (2023). Mitochondrial control of inflammation. *Nat. Rev. Immunol.* 23, 159–173. 10.1038/s41577-022-00760-x 35879417 PMC9310369

[B92] Mastrostefano A. Alborghetti M. Tiso D. Davinelli S. Medoro A. Scapagnini G. (2025). Pleiotropic actions of gastrodia elata glucosides in the treatment of painful neuropathies and CNS disorders: Focus on mitochondrial dysfunction and modulation of ion channels. *Curr. Neuropharmacol.* 23, 10.2174/011570159x402568251002112117 41140221

[B93] Mattson M. P. (2008). Glutamate and neurotrophic factors in neuronal plasticity and disease. *Ann. N. Y. Acad. Sci.* 1144, 97–112. 10.1196/annals.1418.005 19076369 PMC2614307

[B94] Moskowitz M. A. Lo E. H. Iadecola C. (2010). The science of stroke: Mechanisms in search of treatments. *Neuron* 67, 181–198. 10.1016/j.neuron.2010.07.002 20670828 PMC2957363

[B95] Mou Y. Du Y. Zhou L. Yue J. Hu X. Liu Y. et al. (2022). Gut Microbiota interact with the brain through systemic chronic inflammation: Implications on neuroinflammation, neurodegeneration, and aging. *Front. Immunol.* 13:796288. 10.3389/fimmu.2022.796288 35464431 PMC9021448

[B96] Mrak R. E. Griffin W. S. (2007). Common inflammatory mechanisms in lewy body disease and Alzheimer disease. *J. Neuropathol. Exp. Neurol.* 66, 683–686. 10.1097/nen.0b013e31812503e1 17882012

[B97] Narantuya D. Nagai A. Sheikh A. M. Wakabayashi K. Shiota Y. Watanabe T. et al. (2010). Microglia transplantation attenuates white matter injury in rat chronic ischemia model via matrix metalloproteinase-2 inhibition. *Brain Res.* 1316, 145–152. 10.1016/j.brainres.2009.12.043 20036218

[B98] Nasb M. Tao W. Chen N. (2024). Alzheimer’s disease puzzle: Delving into pathogenesis hypotheses. *Aging. Dis.* 15, 43–73. 10.14336/ad.2023.0608 37450931 PMC10796101

[B99] Nepal M. R. Jeong K. S. Kim G. H. Cha D. H. Kang M. J. Kim J. S. et al. (2019). Role of intestinal microbiota in metabolism of gastrodin in vitro and in vivo. *Metabolites* 9:69. 10.3390/metabo9040069 30965644 PMC6523420

[B100] Nissen M. S. Ryding M. Meyer M. Blaabjerg M. (2020). Autoimmune encephalitis: Current knowledge on subtypes, disease mechanisms and treatment. *CNS Neurol. Disord. Drug. Targets* 19, 584–598. 10.2174/1871527319666200708133103 32640967

[B101] O’Shea T. M. Ao Y. Wang S. Ren Y. Cheng A. L. Kawaguchi R. et al. (2024). Derivation and transcriptional reprogramming of border-forming wound repair astrocytes after spinal cord injury or stroke in mice. *Nat. Neurosci.* 27, 1505–1521. 10.1038/s41593-024-01684-6 38907165 PMC11303254

[B102] Ozaki E. Campbell M. Doyle S. L. (2015). Targeting the NLRP3 inflammasome in chronic inflammatory diseases: Current perspectives. *J. Inflamm. Res.* 8, 15–27. 10.2147/jir.S51250 25653548 PMC4303395

[B103] Peng Y. Ye J. R. Wang S. S. He W. B. Feng Z. P. Sun H. S. et al. (2023). A small molecule 20C from gastrodia elata inhibits α-synuclein aggregation and prevents progression of Parkinson’s disease. *Cell. Death Dis.* 14:594. 10.1038/s41419-023-06116-0 37673867 PMC10482970

[B104] Percie du Sert N. Hurst V. Ahluwalia A. Alam S. Avey M. T. Baker M. et al. (2020). The ARRIVE guidelines 2.0: updated guidelines for reporting animal research. *PLoS Biol.* 18:e3000410. 10.1371/journal.pbio.3000410 32663219 PMC7360023

[B105] Perucca E. White H. S. Bialer M. (2023). New GABA-targeting therapies for the treatment of seizures and epilepsy: II. Treatments in clinical development. *CNS Drugs* 37, 781–795. 10.1007/s40263-023-01025-4 37603261 PMC10501930

[B106] Pober J. S. Sessa W. C. (2014). Inflammation and the blood microvascular system. *Cold. Spring Harb. Perspect. Biol.* 7:a016345. 10.1101/cshperspect.a016345 25384307 PMC4292166

[B107] Priem D. van Loo G. Bertrand M. J. M. (2020). A20 and cell death-driven inflammation. *Trends Immunol.* 41, 421–435. 10.1016/j.it.2020.03.001 32241683

[B108] Prinz M. Priller J. (2014). Microglia and brain macrophages in the molecular age: From origin to neuropsychiatric disease. *Nat. Rev. Neurosci.* 15, 300–312. 10.1038/nrn3722 24713688

[B109] Ransohoff R. M. (2016). How neuroinflammation contributes to neurodegeneration. *Science* 353, 777–783. 10.1126/science.aag2590 27540165

[B110] Ravizza T. Scheper M. Di Sapia R. Gorter J. Aronica E. Vezzani A. (2024). mTOR and neuroinflammation in epilepsy: Implications for disease progression and treatment. *Nat. Rev. Neurosci.* 25, 334–350. 10.1038/s41583-024-00805-1 38531962

[B111] Savica R. Carlin J. M. Grossardt B. R. Bower J. H. Ahlskog J. E. Maraganore D. M. et al. (2009). Medical records documentation of constipation preceding Parkinson disease: A case-control study. *Neurology* 73, 1752–1758. 10.1212/WNL.0b013e3181c34af5 19933976 PMC2788809

[B112] Scheiblich H. Bousset L. Schwartz S. Griep A. Latz E. Melki R. et al. (2021). Microglial NLRP3 inflammasome activation upon TLR2 and TLR5 ligation by distinct α-synuclein assemblies. *J. Immunol.* 207, 2143–2154. 10.4049/jimmunol.2100035 34507948 PMC8490941

[B113] Scheller J. Chalaris A. Schmidt-Arras D. Rose-John S. (2011). The pro- and anti-inflammatory properties of the cytokine interleukin-6. *Biochim. Biophys. Acta.* 1813, 878–888. 10.1016/j.bbamcr.2011.01.034 21296109

[B114] Sha S. Tan J. Miao Y. Zhang Q. (2021). The role of autophagy in hypoxia-induced neuroinflammation. *DNA Cell. Biol.* 40, 733–739. 10.1089/dna.2020.6186 33989049

[B115] Shao J. Xu Q. Luo Y. Dong T. Han X. Chen B. et al. (2025). Pharmacokinetics and brain tissue distribution of gastrodia elata extract in normal and cerebral ischemic rats: A comparative study. *Front. Pharmacol.* 16:1624576. 10.3389/fphar.2025.1624576 40822475 PMC12350473

[B116] Shao S. Xu C. B. Chen C. J. Shi G. N. Guo Q. L. Zhou Y. et al. (2021). Divanillyl sulfone suppresses NLRP3 inflammasome activation via inducing mitophagy to ameliorate chronic neuropathic pain in mice. *J. Neuroinflammation* 18:142. 10.1186/s12974-021-02178-z 34162415 PMC8223331

[B117] Shi A. Xiang J. He F. Zhu Y. Zhu G. Lin Y. et al. (2018). The phenolic components of gastrodia elata improve prognosis in rats after cerebral ischemia/reperfusion by enhancing the endogenous antioxidant mechanisms. *Oxid. Med. Cell. Longev.* 2018:7642158. 10.1155/2018/7642158 29765502 PMC5885496

[B118] Shi F. D. Yong V. W. (2025). Neuroinflammation across neurological diseases. *Science* 388:eadx0043. 10.1126/science.adx0043 40536983

[B119] Shi Q. Xue C. Zeng Y. Yuan X. Chu Q. Jiang S. et al. (2024). Notch signaling pathway in cancer: From mechanistic insights to targeted therapies. *Signal Transduct Target Ther.* 9:128. 10.1038/s41392-024-01828-x 38797752 PMC11128457

[B120] Shi R. Zheng C. B. Wang H. Rao Q. Du T. Bai C. et al. (2020). Gastrodin alleviates vascular dementia in a 2-VO-vascular dementia rat model by altering amyloid and tau levels. *Pharmacology* 105, 386–396. 10.1159/000504056 31752010

[B121] Shi X. Yu X. Yang L. Duan X. (2023). Ethyl acetate extract of Gastrodia elata protects caenorhabditis elegans from oxidative stress and amyloid β peptide toxicity. *Exp. Ther. Med.* 26:405. 10.3892/etm.2023.12104 37522064 PMC10375435

[B122] Shi Z. Zhang Y. Xiao Y. Shi Z. Wei X. Wang B. et al. (2024). The protective effects of gastrodin on neurological disorders: An update and future perspectives. *Front. Pharmacol.* 15:1494277. 10.3389/fphar.2024.1494277 39776583 PMC11703667

[B123] Shin E. J. Bach J. H. Nguyen T. T. Jung B. D. Oh K. W. Kim M. J. et al. (2011). Gastrodia elata Bl attenuates cocaine-induced conditioned place preference and convulsion, but not behavioral sensitization in mice: Importance of GABA(A) receptors. *Curr. Neuropharmacol.* 9, 26–29. 10.2174/157015911795017326 21886556 PMC3137195

[B124] Simons M. Nave K. A. (2015). Oligodendrocytes: Myelination and axonal support. *Cold. Spring Harb. Perspect. Biol.* 8:a020479. 10.1101/cshperspect.a020479 26101081 PMC4691794

[B125] Sui Y. Bian L. Ai Q. Yao Y. Yu M. Gao H. et al. (2019). Gastrodin inhibits inflammasome through the STAT3 signal pathways in TNA2 astrocytes and reactive astrocytes in experimentally induced cerebral ischemia in rats. *Neuromolecular. Med.* 21, 275–286. 10.1007/s12017-019-08544-8 31218587

[B126] Sun M. Liu H. Hou Z. Wang Q. Zhong W. (2026). Parishin B attenuates PTZ-induced seizures in zebrafish and is associated with neurotransmitter balance and ACLY-related metabolic pathways. *Metabolites* 16, 275. 10.3390/metabo16040275 42042920 PMC13117408

[B127] Sun Q. Hampel H. Blennow K. Lista S. Levey A. Tang B. et al. (2014). Increased plasma TACE activity in subjects with mild cognitive impairment and patients with Alzheimer’s disease. *J. Alzheimers Dis.* 41, 877–886. 10.3233/jad-140177 24685635 PMC4153789

[B128] Sun W. Lu H. Lyu L. Yang P. Lin Z. Li L. et al. (2019). Gastrodin ameliorates microvascular reperfusion injury-induced pyroptosis by regulating the NLRP3/caspase-1 pathway. *J. Physiol. Biochem.* 75, 531–547. 10.1007/s13105-019-00702-7 31440987

[B129] Tan W. Zheng Q. Feng K. Feng X. Zhong W. Liao C. et al. (2022). Neuroprotection of Gastrodia elata polyphenols against H(2)O(2)-induced PC12 cell cytotoxicity by reducing oxidative stress. *Front. Pharmacol.* 13:1050775. 10.3389/fphar.2022.1050775 36438797 PMC9684467

[B130] Tang C. Wang L. Liu X. Cheng M. Xiao H. (2015). Pharmacokinetic study of gastrodia elata in rats. *Anal. Bioanal. Chem.* 407, 8903–8910. 10.1007/s00216-015-9054-y 26416021

[B131] Tang C. Wang L. Liu X. Cheng M. Qu Y. Xiao H. (2015). Comparative pharmacokinetics of gastrodin in rats after intragastric administration of free gastrodin, parishin and gastrodia elata extract. *J. Ethnopharmacol.* 176, 49–54. 10.1016/j.jep.2015.10.007 26471288

[B132] Tao J. Xiao T. Zhang Z. Cheng J. Tan J. Zhao Z. et al. (2026). Multi-omics reveals the protective mechanisms of Gastrodia elata ethyl acetate extract in vascular dementia. *Front. Pharmacol.* 17:1630783. 10.3389/fphar.2026.1630783 41660514 PMC12876206

[B133] Taoka T. Masutani Y. Kawai H. Nakane T. Matsuoka K. Yasuno F. et al. (2017). Evaluation of glymphatic system activity with the diffusion MR technique: Diffusion tensor image analysis along the perivascular space (DTI-ALPS) in Alzheimer’s disease cases. *Jpn. J. Radiol.* 35, 172–178. 10.1007/s11604-017-0617-z 28197821

[B134] Thakur S. Dhapola R. Sarma P. Medhi B. Reddy D. H. (2023). Neuroinflammation in Alzheimer’s disease: Current progress in molecular signaling and therapeutics. *Inflammation* 46, 1–17. 10.1007/s10753-022-01721-1 35986874

[B135] Tian Z. Ji X. Liu J. (2022). Neuroinflammation in vascular cognitive impairment and dementia: Current evidence, advances, and prospects. *Int. J. Mol. Sci.* 23:6224. 10.3390/ijms23116224 35682903 PMC9181710

[B136] Turovsky E. A. Varlamova E. G. Plotnikov E. Y. (2021). Mechanisms underlying the protective effect of the peroxiredoxin-6 are mediated via the protection of astrocytes during ischemia/reoxygenation. *Int. J. Mol. Sci.* 22:8805. 10.3390/ijms22168805 34445509 PMC8396200

[B137] Tuttolomondo A. Puleo M. G. Velardo M. C. Corpora F. Daidone M. Pinto A. (2020). Molecular biology of atherosclerotic ischemic strokes. *Int. J. Mol. Sci.* 21:9372. 10.3390/ijms21249372 33317034 PMC7763838

[B138] Udi O. A. Omuruka T. C. (2025). Gastrodia elata as a neuroprotective agent: A mini-review of its bioactive compounds and mechanisms of action. *Int. Neuropsychiatr. Dis. J.* 22, 33–42. 10.9734/indj/2025/v22i2473

[B139] Wang D. Liu W. Lu M. Xu Q. (2025). Neuropharmacological effects of gastrodia elata blume and its active ingredients. *Front. Neurol.* 16:1574277. 10.3389/fneur.2025.1574277 40371076 PMC12074926

[B140] Wang L. Li B. Tang Z. Wang Y. Peng Y. Sun T. et al. (2025). Gastrodin alleviates tau pathology by targeting the Alzheimer’s risk gene FERMT2, reversing the reduction in brain viscoelasticity. *CNS Neurosci. Ther.* 31:e70283. 10.1111/cns.70283 40119586 PMC11928745

[B141] Wang L. Wang H. Duan Z. Zhang J. Zhang W. (2018). Mechanism of gastrodin in cell apoptosis in rat hippocampus tissue induced by desflurane. *Exp. Ther. Med.* 15, 2767–2772. 10.3892/etm.2018.5770 29541166 PMC5838295

[B142] Wang N. Liang H. Zen K. (2014). Molecular mechanisms that influence the macrophage m1-m2 polarization balance. *Front. Immunol.* 5:614. 10.3389/fimmu.2014.00614 25506346 PMC4246889

[B143] Wang Q. Chen G. Zeng S. (2007). Pharmacokinetics of gastrodin in rat plasma and CSF after i.n. and i.v. *Int. J. Pharm.* 341, 20–25. 10.1016/j.ijpharm.2007.03.041 17482780

[B144] Wang T. Chen H. Xia S. Chen X. Sun H. Xu Z. (2021). Ameliorative effect of parishin c against cerebral ischemia-induced brain tissue injury by reducing oxidative stress and inflammatory responses in rat model. *Neuropsychiatr. Dis. Treat.* 17, 1811–1823. 10.2147/ndt.S309065 34113111 PMC8187103

[B145] Wang W. Wang Y. Wang F. Xie G. Liu S. Li Z. et al. (2024). Gastrodin regulates the TLR4/TRAF6/NF-κB pathway to reduce neuroinflammation and microglial activation in an AD model. *Phytomedicine* 128:155518. 10.1016/j.phymed.2024.155518 38552431

[B146] Wang Y. Liu J. Wu W. Xue J. Mu D. Wang F. et al. (2026). Ethanol extract of gastrodia elata blume alleviates ischemic stroke by inhibiting oxidative stress and ferroptosis through the PI3K/AKT/Nrf2 pathway. *J. Ethnopharmacol.* 358:120956. 10.1016/j.jep.2025.120956 41285231

[B147] Wang Y. Luo K. Li J. Liao Y. Liao C. Chen W. S. et al. (2022). Focused ultrasound promotes the delivery of gastrodin and enhances the protective effect on dopaminergic neurons in a mouse model of Parkinson’s disease. *Front. Cell. Neurosci.* 16:884788. 10.3389/fncel.2022.884788 35656407 PMC9152004

[B148] Wang Y. Wu W. Wu X. Shah B. A. Lombardo M. Ye G. (2025). Parishin C attenuates oxidative stress and inflammation in HT22 hippocampal neurons and BV2 microglia through Nrf2 signaling pathway. *Int. J. Mol. Sci.* 26:7263. 10.3390/ijms26157263 40806395 PMC12346758

[B149] Wang Y. Zhao M. Xie C. Li L. Lin L. Li Q. et al. (2024). Fermented Gastrodia elata Bl. Alleviates cognitive deficits by regulating neurotransmitters and gut microbiota in D-Gal/AlCl(3)-induced Alzheimer’s disease-like mice. *Foods* 13:2154. 10.3390/foods13132154 38998659 PMC11241452

[B150] Wiatrak B. Kubis-Kubiak A. Piwowar A. Barg E. (2020). PC12 cell line: Cell types, coating of culture vessels, differentiation and other culture conditions. *Cells* 9:958. 10.3390/cells9040958 32295099 PMC7227003

[B151] Wolters F. J. Ikram M. A. (2019). Epidemiology of vascular dementia. *Arterioscler. Thromb. Vasc. Biol.* 39, 1542–1549. 10.1161/atvbaha.119.311908 31294622

[B152] Wong C. H. Crack P. J. (2008). Modulation of neuro-inflammation and vascular response by oxidative stress following cerebral ischemia-reperfusion injury. *Curr. Med. Chem.* 15, 1–14. 10.2174/092986708783330665 18220759

[B153] Wu L. Xiong X. Wu X. Ye Y. Jian Z. Zhi Z. et al. (2020). Targeting oxidative stress and inflammation to prevent ischemia-reperfusion injury. *Front. Mol. Neurosci.* 13:28. 10.3389/fnmol.2020.00028 32194375 PMC7066113

[B154] Wu S. Huang R. Zhang R. Xiao C. Wang L. Luo M. et al. (2023). Gastrodin and gastrodigenin improve energy metabolism disorders and mitochondrial dysfunction to antagonize vascular dementia. *Molecules* 28:2598. 10.3390/molecules28062598 36985572 PMC10059574

[B155] Wu S. Ren Z. Yang M. Xiong Y. Li X. Wu X. et al. (2025). Gastrodin injection relieves hypoxic-ischemic brain injury in newborn rats by regulating the P62/Nrf2/HO-1 pathway. *Mol. Neurobiol.* 63:150. 10.1007/s12035-025-05522-z 41276691 PMC12641047

[B156] Xia C. Zhou H. Xu X. Jiang T. Li S. Wang D. et al. (2020). Identification and Investigation of miRNAs from gastrodia elata blume and their potential function. *Front. Pharmacol.* 11:542405. 10.3389/fphar.2020.542405 33101016 PMC7545038

[B157] Xiao G. Tang R. Yang N. Chen Y. (2023). Review on pharmacological effects of gastrodin. *Arch. Pharm. Res.* 46, 744–770. 10.1007/s12272-023-01463-0 37749449

[B158] Xiao T. Yu X. Tao J. Tan J. Zhao Z. Zhang C. et al. (2025). Mechanism of P-hydroxy benzyl alcohol against cerebral ischemia based on metabonomics analysis. *Int. J. Mol. Sci.* 26:317. 10.3390/ijms26010317 39796170 PMC11719616

[B159] Xicoy H. Wieringa B. Martens G. J. (2017). The SH-SY5Y cell line in Parkinson’s disease research: A systematic review. *Mol. Neurodegener* 12:10. 10.1186/s13024-017-0149-0 28118852 PMC5259880

[B160] Xiong X. Y. Liu L. Yang Q. W. (2018). Refocusing neuroprotection in cerebral reperfusion era: New challenges and strategies. *Front. Neurol.* 9:249. 10.3389/fneur.2018.00249 29740385 PMC5926527

[B161] Xu L. He G. P. Li A. Ro H. S. (1994). Molecular characterization of the mouse ribosomal protein S24 multigene family: A uniquely expressed intron-containing gene with cell-specific expression of three alternatively spliced mRNAs. *Nucleic. Acids. Res.* 22, 646–655. 10.1093/nar/22.4.646 8127713 PMC307856

[B162] Xu R. Wang J. Xu J. Song X. Huang H. Feng Y. et al. (2020). Rhynchophylline loaded-mPEG-PLGA nanoparticles coated with tween-80 for preliminary study in Alzheimer’s disease. *Int. J. Nanomedicine.* 15, 1149–1160. 10.2147/ijn.S236922 32110013 PMC7035889

[B163] Yamamoto M. Kiyota T. Horiba M. Buescher J. L. Walsh S. M. Gendelman H. E. et al. (2007). Interferon-gamma and tumor necrosis factor-alpha regulate amyloid-beta plaque deposition and beta-secretase expression in Swedish mutant APP transgenic mice. *Am. J. Pathol.* 170, 680–692. 10.2353/ajpath.2007.060378 17255335 PMC1851864

[B164] Yan J. Yang Z. Zhao N. Li Z. Cao X. (2019). Gastrodin protects dopaminergic neurons via insulin-like pathway in a Parkinson’s disease model. *BMC Neurosci.* 20:31. 10.1186/s12868-019-0512-x 31208386 PMC6580469

[B165] Yang C. S. Chiu S. C. Liu P. Y. Wu S. N. Lai M. C. Huang C. W. (2021). Gastrodin alleviates seizure severity and neuronal excitotoxicities in the rat lithium-pilocarpine model of temporal lobe epilepsy via enhancing GABAergic transmission. *J. Ethnopharmacol* 269, 113751. 10.1016/j.jep.2020.113751 33359863

[B166] Yang C. Hawkins K. E. Doré S. Candelario-Jalil E. (2019). Neuroinflammatory mechanisms of blood-brain barrier damage in ischemic stroke. *Am. J. Physiol. Cell. Physiol.* 316, C135–C153. 10.1152/ajpcell.00136.2018 30379577 PMC6397344

[B167] Yang L. Tsai T. H. (2022). Neuroprotective effect and herbal-drug pharmacokinetic interaction of Gastrodia elata extract on valproic acid. *Biomed. Pharmacother* 156:113938. 10.1016/j.biopha.2022.113938 36411625

[B168] Yang Y. Estrada E. Y. Thompson J. F. Liu W. Rosenberg G. A. (2007). Matrix metalloproteinase-mediated disruption of tight junction proteins in cerebral vessels is reversed by synthetic matrix metalloproteinase inhibitor in focal ischemia in rat. *J. Cereb. Blood Flow. Metab.* 27, 697–709. 10.1038/sj.jcbfm.9600375 16850029

[B169] Yao W. Cao Q. Luo S. He L. Yang C. Chen J. et al. (2022). Microglial ERK-NRBP1-CREB-BDNF signaling in sustained antidepressant actions of (R)-ketamine. *Mol. Psychiatry* 27, 1618–1629. 10.1038/s41380-021-01377-7 34819637 PMC9095473

[B170] Yao Y. Y. Ling E. A. Lu D. (2020). Microglia mediated neuroinflammation - signaling regulation and therapeutic considerations with special reference to some natural compounds. *Histol. Histopathol.* 35, 1229–1250. 10.14670/hh-18-239 32662061

[B171] Yip K. L. Koon C. M. Chen Z. Y. Chook P. Leung P. C. Schachter S. et al. (2020). The antiepileptic effect of gastrodiae rhizoma through modulating overexpression of mTOR and attenuating astrogliosis in pilocarpine mice model. *Epilepsia. Open* 5, 50–60. 10.1002/epi4.12372 32140643 PMC7049815

[B172] Yu X. Tao J. Xiao T. Duan X. (2024a). P-hydroxybenzaldehyde protects caenorhabditis elegans from oxidative stress and β-amyloid toxicity. *Front. Aging. Neurosci.* 16:1414956. 10.3389/fnagi.2024.1414956 38841104 PMC11150654

[B173] Yu X. Tao J. Xiao T. Duan X. (2024b). 4,4’-methylenediphenol reduces Aβ-induced toxicity in a caenorhabditis elegans model of Alzheimer’s disease. *Front. Aging. Neurosci.* 16:1393721. 10.3389/fnagi.2024.1393721 38872629 PMC11171718

[B174] Yu Y. Tang L. Cui F. Jiao F. Zhang D. Ma J. et al. (2021). Effect of qizhitongluo capsule on lower limb rehabilitation after stroke: A randomized clinical trial. *Pharmacol. Res.* 165:105464. 10.1016/j.phrs.2021.105464 33515707

[B175] Zhang D. Y. Cheng Y. B. Guo Q. H. Shan X. L. Wei F. F. Lu F. et al. (2020). Treatment of masked hypertension with a chinese herbal formula: A randomized, placebo-controlled trial. *Circulation* 142, 1821–1830. 10.1161/circulationaha.120.046685 33019798

[B176] Zhang D. Wu J. Feng H. Tang P. Zhou Y. Zhao C. et al. (2025). Gastrodin ameliorates ulcerative colitis via modulating gut microbial tryptophan metabolism and AhR/NLRP3 pathway. *Phytomedicine* 147:157217. 10.1016/j.phymed.2025.157217 40907403

[B177] Zhang H. S. Liu M. F. Ji X. Y. Jiang C. R. Li Z. L. OuYang B. (2019). Gastrodin combined with rhynchophylline inhibits cerebral ischaemia-induced inflammasome activation via upregulating miR-21-5p and miR-331-5p. *Life Sci.* 239:116935. 10.1016/j.lfs.2019.116935 31610203

[B178] Zhang J. S. Zhou S. F. Wang Q. Guo J. N. Liang H. M. Deng J. B. et al. (2016). Gastrodin suppresses BACE1 expression under oxidative stress condition via inhibition of the PKR/eIF2α pathway in Alzheimer’s disease. *Neuroscience* 325, 1–9. 10.1016/j.neuroscience.2016.03.024 26987953

[B179] Zhang M. Zhang Y. Peng J. Huang Y. Gong Z. Lu H. et al. (2024a). Gastrodin against oxidative stress-inflammation crosstalk via inhibiting mtDNA/TLR9 and JAK2/STAT3 signaling to ameliorate ischemic stroke injury. *Int. Immunopharmacol.* 141:113012. 10.1016/j.intimp.2024.113012 39182268

[B180] Zhang R. L. Zhang Z. G. Zhang L. Chopp M. (2001). Proliferation and differentiation of progenitor cells in the cortex and the subventricular zone in the adult rat after focal cerebral ischemia. *Neuroscience* 105, 33–41. 10.1016/s0306-4522(01)00117-8 11483298

[B181] Zhang X. X. Zuo J. Q. Wang Y. T. Duan H. Y. Yuan J. H. Hu Y. H. (2022). Paeoniflorin in Paeoniaceae: Distribution, influencing factors, and biosynthesis. *Front. Plant. Sci.* 13:980854. 10.3389/fpls.2022.980854 36119574 PMC9478390

[B182] Zhang Y. Ye P. Zhu H. Gu L. Li Y. Feng S. et al. (2024b). Neutral polysaccharide from Gastrodia elata alleviates cerebral ischemia-reperfusion injury by inhibiting ferroptosis-mediated neuroinflammation via the NRF2/HO-1 signaling pathway. *CNS Neurosci. Ther.* 30:e14456. 10.1111/cns.14456 37752806 PMC10916450

[B183] Zhao M. Zhou Y. Sheng R. Zhang H. Xiang J. Wang J. et al. (2024). Gastrodin relieves Parkinson’s disease-related motor deficits by facilitating the MEK-dependent VMAT2 to maintain dopamine homeostasis. *Phytomedicine* 132, 155819. 10.1016/j.phymed.2024.155819 38885579

[B184] Zhao Z. Lei M. Bai Y. Qiao H. (2026). Excavating precursors from the traditional Chinese pair herbs polygala tenuifolia and gastrodia elata: Synthesis, anticonvulsant activity evaluation of 3,4,5-trimethoxycinnamic acid (TMCA) peptide analogs. *Pharmaceuticals* 19:265. 10.3390/ph19020265 41754806 PMC12943239

[B185] Zheng X. Gong T. Tang C. Zhong Y. Shi L. Fang X. et al. (2022). Gastrodin improves neuroinflammation-induced cognitive dysfunction in rats by regulating NLRP3 inflammasome. *BMC Anesthesiol.* 22:371. 10.1186/s12871-022-01915-y 36456961 PMC9714247

[B186] Zhou R. Wang Y. Cao X. Yuan X. Li L. Zhang W. (2025). Gastrodin from the gastrodia elata blume alleviates neuronal inflammation and injury via regulates NOX2/Nrf2 balance. *Neuropharmacology* 279:110620. 10.1016/j.neuropharm.2025.110620 40784543

[B187] Zhou X. Han M. Yan S. Wang M. Zu S. Zhong S. (2025). From molecules to medicine: A systematic review of Gastrodia elata’s bioactive metabolites and therapeutic potential. *Front. Pharmacol.* 16:1641443. 10.3389/fphar.2025.1641443 41282615 PMC12634510

[B188] Zhu J. Cui Y. Zhang J. Yan R. Su D. Zhao D. et al. (2024). Temporal trends in the prevalence of Parkinson’s disease from 1980 to 2023: A systematic review and meta-analysis. *Lancet Healthy Longev.* 5, e464–e479. 10.1016/s2666-7568(24)00094-1 38945129

[B189] Zimprich A. Biskup S. Leitner P. Lichtner P. Farrer M. Lincoln S. et al. (2004). Mutations in LRRK2 cause autosomal-dominant parkinsonism with pleomorphic pathology. *Neuron* 44, 601–607. 10.1016/j.neuron.2004.11.005 15541309

