# Supplementary material for: Gastrodia elata in neurological disorders: a systematic review of anti-inflammatory mechanisms and cross-disease therapeutic potential
Source: Front Aging Neurosci. 2026 Oct 26;18:1967946. doi: 10.3389/fnagi.2026.1967946 (PMC13745468; doi:10.3389/fnagi.2026.1967946)
Supplement: Supplementary file 2 [file Table_2.docx]

**Supplementary Material A. PubMed Search Strategy**

1. Information sources

Database: Medline (U.S. National Library of Medicine).

Interface: https://pubmed.ncbi.nlm.nih.gov/ (Advanced Search Builder).

Date of final search: 31 May 2026.

Export format: NBIB (PubMed), imported into EndNote X9 for de-duplication.

2. Search design

The strategy consisted of two Boolean blocks combined with AND: (i) the intervention block (Gastrodia elata and its main bioactive constituents), and (ii) the condition block (each of five neurological diseases). Within each block, Medical Subject Headings (MeSH) terms and free-text synonyms were combined with OR. MeSH terms were auto-exploded by PubMed. Multi-word phrases were searched as quoted phrases in the Title/Abstract field. No date, language, publication-type, or species limits were imposed at the search stage; all such restrictions were applied during screening.

3. Block 1 — Gastrodia elata

(Gastrodia[MeSH Terms]) OR (Gastrodia OR Gastrodias OR Gastrodia elata OR Tien ma)

4. Block 2 — Disease-specific sub-blocks

4.1 Block 2.1 — Parkinson Disease

(Parkinson Disease[MeSH Terms]) OR (Idiopathic Parkinson Disease OR Idiopathic Parkinson's Disease OR Lewy Body Parkinson Disease OR Lewy Body Parkinson's Disease OR Paralysis Agitans OR Parkinson Disease, Idiopathic OR Parkinson's Disease OR Parkinson's Disease, Idiopathic OR Parkinson's Disease, Lewy Body OR Primary Parkinsonism OR Parkinsonism, Primary)

4.2 Block 2.2 — Alzheimer Disease

(Alzheimer Disease[MeSH Terms]) OR (Alzheimer Syndrome OR (Alzheimer-Type Dementia (ATD)) OR (Alzheimer Type Dementia (ATD)) OR (Dementia, Alzheimer-Type (ATD)) OR Alzheimer's Diseases OR Alzheimer Diseases OR Alzheimers Diseases OR Alzheimer Dementia OR Alzheimer Dementias OR Dementia, Alzheimer OR Alzheimer's Disease OR Dementia, Senile OR Senile Dementia OR Dementia, Alzheimer Type OR Alzheimer Type Dementia OR Senile Dementia, Alzheimer Type OR Alzheimer Type Senile Dementia OR Primary Senile Degenerative Dementia OR Alzheimer Sclerosis OR Sclerosis, Alzheimer OR Dementia, Primary Senile Degenerative OR Dementia, Presenile OR Presenile Dementia OR Acute Confusional Senile Dementia OR Senile Dementia, Acute Confusional OR Alzheimer Disease, Early Onset OR Early Onset Alzheimer Disease OR Presenile Alzheimer Dementia OR Alzheimer Disease, Late Onset OR Late Onset Alzheimer Disease OR Alzheimer's Disease, Focal Onset OR Focal Onset Alzheimer's Disease OR (Familial Alzheimer Disease (FAD)) OR (Alzheimer Disease, Familial (FAD)) OR (Familial Alzheimer Diseases (FAD)))

4.3 Block 2.3 — Ischemic Stroke

(Stroke[MeSH Terms]) OR (Stroke OR Strokes OR Cerebrovascular Accident OR Cerebrovascular Accidents OR Cerebral Stroke OR Cerebral Strokes OR Stroke, Cerebral OR Strokes, Cerebral OR Cerebrovascular Apoplexy OR Apoplexy, Cerebrovascular OR Vascular Accident, Brain OR Brain Vascular Accident OR Brain Vascular Accidents OR Vascular Accidents, Brain OR Cerebrovascular Stroke OR Cerebrovascular Strokes OR Stroke, Cerebrovascular OR Strokes, Cerebrovascular OR Apoplexy OR CVA (Cerebrovascular Accident) OR CVAs (Cerebrovascular Accident) OR Stroke, Acute OR Acute Stroke OR Acute Strokes OR Strokes, Acute OR Cerebrovascular Accident, Acute OR Acute Cerebrovascular Accident OR Acute Cerebrovascular Accidents OR Cerebrovascular Accidents, Acute)

4.4 Block 2.4 — Vascular Dementia

(Dementia, Vascular[MeSH Terms]) OR (Dementia, Vascular OR Dementias, Vascular OR Vascular Dementias OR Vascular Dementia OR Arteriosclerotic Dementia OR Arteriosclerotic Dementias OR Dementia, Arteriosclerotic OR Dementias, Arteriosclerotic OR Binswanger Disease OR Disease, Binswanger OR Encephalopathy, Binswanger OR Chronic Progressive Subcortical Encephalopathy OR Binswanger Encephalopathy OR Leukoencephalopathy, Subcortical OR Leukoencephalopathies, Subcortical OR Subcortical Leukoencephalopathies OR Encephalopathy, Subcortical Arteriosclerotic OR Encephalopathy, Chronic Progressive Subcortical OR Encephalopathy, Subcortical, Chronic Progressive OR Subcortical Encephalopathy, Chronic Progressive OR Subcortical Leukoencephalopathy OR Subcortical Arteriosclerotic Encephalopathy OR Arteriosclerotic Encephalopathy, Subcortical OR Arteriosclerotic Encephalopathies, Subcortical OR Encephalopathies, Subcortical Arteriosclerotic OR Subcortical Arteriosclerotic Encephalopathies OR Encephalopathy, Binswanger's OR Binswanger's Encephalopathy OR Encephalopathy, Binswangers OR Binswanger's Disease OR Binswangers Disease OR Disease, Binswanger's OR Subcortical Vascular Dementia OR Dementias, Subcortical Vascular OR Dementia, Subcortical Vascular OR Subcortical Vascular Dementias OR Vascular Dementias, Subcortical OR Vascular Dementia, Subcortical OR Vascular Dementia, Acute Onset OR Acute Onset Vascular Dementia)

4.5 Block 2.5 — Epilepsy

(Epilepsy[MeSH Terms]) OR (Epilepsy OR Epilepsies OR Seizure Disorder OR Seizure Disorders OR Epilepsy, Cryptogenic OR Cryptogenic Epilepsies OR Cryptogenic Epilepsy OR Epilepsies, Cryptogenic OR Aura OR Auras OR Awakening Epilepsy OR Epilepsy, Awakening[MeSH Terms])

5. Final combined PubMed queries (one per disease block)

Each line below is the complete, copy-paste-ready search string for one disease block. Format: Block 1 AND Block 2.

5.1 Equation 1 Gastrodia AND Parkinson Disease

Records retrieved: 46

("gastrodia"[MeSH Terms] OR ("gastrodia elata extract"[Supplementary Concept] OR "gastrodia elata extract"[All Fields] OR "gastrodia"[All Fields] OR "gastrodia"[MeSH Terms] OR "gastrodiae"[All Fields] OR ("gastrodia"[MeSH Terms] OR "gastrodia"[All Fields]) OR ("gastrodia elata extract"[Supplementary Concept] OR "gastrodia elata extract"[All Fields] OR "gastrodia elata"[All Fields] OR "gastrodia"[MeSH Terms] OR "gastrodia"[All Fields] OR ("gastrodia"[All Fields] AND "elata"[All Fields])) OR ("gastrodia"[MeSH Terms] OR "gastrodia"[All Fields] OR ("tien"[All Fields] AND "ma"[All Fields]) OR "tien ma"[All Fields]))) AND ("parkinson disease"[MeSH Terms] OR ("parkinson disease"[MeSH Terms] OR ("parkinson"[All Fields] AND "disease"[All Fields]) OR "parkinson disease"[All Fields] OR ("idiopathic"[All Fields] AND "parkinson"[All Fields] AND "disease"[All Fields]) OR "idiopathic parkinson disease"[All Fields] OR ("parkinson disease"[MeSH Terms] OR ("parkinson"[All Fields] AND "disease"[All Fields]) OR "parkinson disease"[All Fields] OR ("idiopathic"[All Fields] AND "parkinson s"[All Fields] AND "disease"[All Fields]) OR "idiopathic parkinson s disease"[All Fields]) OR ("parkinson disease"[MeSH Terms] OR ("parkinson"[All Fields] AND "disease"[All Fields]) OR "parkinson disease"[All Fields] OR ("lewy"[All Fields] AND "body"[All Fields] AND "parkinson"[All Fields] AND "disease"[All Fields]) OR "lewy body parkinson disease"[All Fields]) OR ("parkinson disease"[MeSH Terms] OR ("parkinson"[All Fields] AND "disease"[All Fields]) OR "parkinson disease"[All Fields] OR ("lewy"[All Fields] AND "body"[All Fields] AND "parkinson s"[All Fields] AND "disease"[All Fields]) OR "lewy body parkinson s disease"[All Fields]) OR ("parkinson disease"[MeSH Terms] OR ("parkinson"[All Fields] AND "disease"[All Fields]) OR "parkinson disease"[All Fields] OR ("paralysis"[All Fields] AND "agitans"[All Fields]) OR "paralysis agitans"[All Fields]) OR ("parkinson disease"[MeSH Terms] OR ("parkinson"[All Fields] AND "disease"[All Fields]) OR "parkinson disease"[All Fields] OR ("parkinson"[All Fields] AND "disease"[All Fields] AND "idiopathic"[All Fields]) OR "parkinson disease idiopathic"[All Fields]) OR ("parkinson disease"[MeSH Terms] OR ("parkinson"[All Fields] AND "disease"[All Fields]) OR "parkinson disease"[All Fields] OR ("parkinson s"[All Fields] AND "disease"[All Fields]) OR "parkinson s disease"[All Fields]) OR ("parkinson disease"[MeSH Terms] OR ("parkinson"[All Fields] AND "disease"[All Fields]) OR "parkinson disease"[All Fields] OR ("parkinson s"[All Fields] AND "disease"[All Fields] AND "idiopathic"[All Fields]) OR "parkinson s disease idiopathic"[All Fields]) OR ("parkinson disease"[MeSH Terms] OR ("parkinson"[All Fields] AND "disease"[All Fields]) OR "parkinson disease"[All Fields] OR ("parkinson s"[All Fields] AND "disease"[All Fields] AND "lewy"[All Fields] AND "body"[All Fields]) OR "parkinson s disease lewy body"[All Fields]) OR ("parkinson disease"[MeSH Terms] OR ("parkinson"[All Fields] AND "disease"[All Fields]) OR "parkinson disease"[All Fields] OR ("primary"[All Fields] AND "parkinsonism"[All Fields]) OR "primary parkinsonism"[All Fields]) OR ("parkinson disease"[MeSH Terms] OR ("parkinson"[All Fields] AND "disease"[All Fields]) OR "parkinson disease"[All Fields] OR ("parkinsonism"[All Fields] AND "primary"[All Fields]) OR "parkinsonism primary"[All Fields])))

5.2 Equation 2 — Gastrodia AND Alzheimer Disease

Records retrieved: 65

("alzheimer disease"[MeSH Terms] OR ("alzheimer disease"[MeSH Terms] OR ("alzheimer"[All Fields] AND "disease"[All Fields]) OR "alzheimer disease"[All Fields] OR ("alzheimer"[All Fields] AND "syndrome"[All Fields]) OR "alzheimer syndrome"[All Fields] OR (("alzheimer disease"[MeSH Terms] OR ("alzheimer"[All Fields] AND "disease"[All Fields]) OR "alzheimer disease"[All Fields] OR ("alzheimer"[All Fields] AND "type"[All Fields] AND "dementia"[All Fields]) OR "alzheimer type dementia"[All Fields]) AND "ATD"[All Fields]) OR (("alzheimer disease"[MeSH Terms] OR ("alzheimer"[All Fields] AND "disease"[All Fields]) OR "alzheimer disease"[All Fields] OR ("alzheimer"[All Fields] AND "type"[All Fields] AND "dementia"[All Fields]) OR "alzheimer type dementia"[All Fields]) AND "ATD"[All Fields]) OR (("alzheimer disease"[MeSH Terms] OR ("alzheimer"[All Fields] AND "disease"[All Fields]) OR "alzheimer disease"[All Fields] OR ("dementia"[All Fields] AND "alzheimer"[All Fields] AND "type"[All Fields]) OR "dementia alzheimer type"[All Fields]) AND "ATD"[All Fields]) OR ("alzheimer disease"[MeSH Terms] OR ("alzheimer"[All Fields] AND "disease"[All Fields]) OR "alzheimer disease"[All Fields] OR ("alzheimer s"[All Fields] AND "diseases"[All Fields]) OR "alzheimer s diseases"[All Fields]) OR ("alzheimer disease"[MeSH Terms] OR ("alzheimer"[All Fields] AND "disease"[All Fields]) OR "alzheimer disease"[All Fields] OR ("alzheimer"[All Fields] AND "diseases"[All Fields]) OR "alzheimer diseases"[All Fields]) OR ("alzheimer disease"[MeSH Terms] OR ("alzheimer"[All Fields] AND "disease"[All Fields]) OR "alzheimer disease"[All Fields] OR ("alzheimers"[All Fields] AND "diseases"[All Fields]) OR "alzheimers diseases"[All Fields]) OR ("alzheimer disease"[MeSH Terms] OR ("alzheimer"[All Fields] AND "disease"[All Fields]) OR "alzheimer disease"[All Fields] OR ("alzheimer"[All Fields] AND "dementia"[All Fields]) OR "alzheimer dementia"[All Fields]) OR ("alzheimer disease"[MeSH Terms] OR ("alzheimer"[All Fields] AND "disease"[All Fields]) OR "alzheimer disease"[All Fields] OR ("alzheimer"[All Fields] AND "dementias"[All Fields]) OR "alzheimer dementias"[All Fields]) OR ("alzheimer disease"[MeSH Terms] OR ("alzheimer"[All Fields] AND "disease"[All Fields]) OR "alzheimer disease"[All Fields] OR ("dementia"[All Fields] AND "alzheimer"[All Fields]) OR "dementia alzheimer"[All Fields]) OR ("alzheimer disease"[MeSH Terms] OR ("alzheimer"[All Fields] AND "disease"[All Fields]) OR "alzheimer disease"[All Fields] OR ("alzheimer s"[All Fields] AND "disease"[All Fields]) OR "alzheimer s disease"[All Fields]) OR ("alzheimer disease"[MeSH Terms] OR ("alzheimer"[All Fields] AND "disease"[All Fields]) OR "alzheimer disease"[All Fields] OR ("dementia"[All Fields] AND "senile"[All Fields]) OR "dementia senile"[All Fields]) OR ("alzheimer disease"[MeSH Terms] OR ("alzheimer"[All Fields] AND "disease"[All Fields]) OR "alzheimer disease"[All Fields] OR ("senile"[All Fields] AND "dementia"[All Fields]) OR "senile dementia"[All Fields]) OR ("alzheimer disease"[MeSH Terms] OR ("alzheimer"[All Fields] AND "disease"[All Fields]) OR "alzheimer disease"[All Fields] OR ("dementia"[All Fields] AND "alzheimer"[All Fields] AND "type"[All Fields]) OR "dementia alzheimer type"[All Fields]) OR ("alzheimer disease"[MeSH Terms] OR ("alzheimer"[All Fields] AND "disease"[All Fields]) OR "alzheimer disease"[All Fields] OR ("alzheimer"[All Fields] AND "type"[All Fields] AND "dementia"[All Fields]) OR "alzheimer type dementia"[All Fields]) OR ("alzheimer disease"[MeSH Terms] OR ("alzheimer"[All Fields] AND "disease"[All Fields]) OR "alzheimer disease"[All Fields] OR ("senile"[All Fields] AND "dementia"[All Fields] AND "alzheimer"[All Fields] AND "type"[All Fields]) OR "senile dementia alzheimer type"[All Fields]) OR ("alzheimer disease"[MeSH Terms] OR ("alzheimer"[All Fields] AND "disease"[All Fields]) OR "alzheimer disease"[All Fields] OR ("alzheimer"[All Fields] AND "type"[All Fields] AND "senile"[All Fields] AND "dementia"[All Fields]) OR "alzheimer type senile dementia"[All Fields]) OR ("alzheimer disease"[MeSH Terms] OR ("alzheimer"[All Fields] AND "disease"[All Fields]) OR "alzheimer disease"[All Fields] OR ("primary"[All Fields] AND "senile"[All Fields] AND "degenerative"[All Fields] AND "dementia"[All Fields]) OR "primary senile degenerative dementia"[All Fields]) OR ("alzheimer disease"[MeSH Terms] OR ("alzheimer"[All Fields] AND "disease"[All Fields]) OR "alzheimer disease"[All Fields] OR ("alzheimer"[All Fields] AND "sclerosis"[All Fields]) OR "alzheimer sclerosis"[All Fields]) OR ("alzheimer disease"[MeSH Terms] OR ("alzheimer"[All Fields] AND "disease"[All Fields]) OR "alzheimer disease"[All Fields] OR ("sclerosis"[All Fields] AND "alzheimer"[All Fields]) OR "sclerosis alzheimer"[All Fields]) OR ("alzheimer disease"[MeSH Terms] OR ("alzheimer"[All Fields] AND "disease"[All Fields]) OR "alzheimer disease"[All Fields] OR ("dementia"[All Fields] AND "primary"[All Fields] AND "senile"[All Fields] AND "degenerative"[All Fields]) OR "dementia primary senile degenerative"[All Fields]) OR ("alzheimer disease"[MeSH Terms] OR ("alzheimer"[All Fields] AND "disease"[All Fields]) OR "alzheimer disease"[All Fields] OR ("dementia"[All Fields] AND "presenile"[All Fields]) OR "dementia presenile"[All Fields]) OR ("alzheimer disease"[MeSH Terms] OR ("alzheimer"[All Fields] AND "disease"[All Fields]) OR "alzheimer disease"[All Fields] OR ("presenile"[All Fields] AND "dementia"[All Fields]) OR "presenile dementia"[All Fields]) OR ("alzheimer disease"[MeSH Terms] OR ("alzheimer"[All Fields] AND "disease"[All Fields]) OR "alzheimer disease"[All Fields] OR ("acute"[All Fields] AND "confusional"[All Fields] AND "senile"[All Fields] AND "dementia"[All Fields]) OR "acute confusional senile dementia"[All Fields]) OR ("alzheimer disease"[MeSH Terms] OR ("alzheimer"[All Fields] AND "disease"[All Fields]) OR "alzheimer disease"[All Fields] OR ("senile"[All Fields] AND "dementia"[All Fields] AND "acute"[All Fields] AND "confusional"[All Fields])) OR ("alzheimer disease"[MeSH Terms] OR ("alzheimer"[All Fields] AND "disease"[All Fields]) OR "alzheimer disease"[All Fields] OR ("alzheimer"[All Fields] AND "disease"[All Fields] AND "early"[All Fields] AND "onset"[All Fields]) OR "alzheimer disease early onset"[All Fields]) OR ("alzheimer disease"[MeSH Terms] OR ("alzheimer"[All Fields] AND "disease"[All Fields]) OR "alzheimer disease"[All Fields] OR ("early"[All Fields] AND "onset"[All Fields] AND "alzheimer"[All Fields] AND "disease"[All Fields]) OR "early onset alzheimer disease"[All Fields]) OR ("alzheimer disease"[MeSH Terms] OR ("alzheimer"[All Fields] AND "disease"[All Fields]) OR "alzheimer disease"[All Fields] OR ("presenile"[All Fields] AND "alzheimer"[All Fields] AND "dementia"[All Fields]) OR "presenile alzheimer dementia"[All Fields]) OR ("alzheimer disease"[MeSH Terms] OR ("alzheimer"[All Fields] AND "disease"[All Fields]) OR "alzheimer disease"[All Fields] OR ("alzheimer"[All Fields] AND "disease"[All Fields] AND "late"[All Fields] AND "onset"[All Fields]) OR "alzheimer disease late onset"[All Fields]) OR ("alzheimer disease"[MeSH Terms] OR ("alzheimer"[All Fields] AND "disease"[All Fields]) OR "alzheimer disease"[All Fields] OR ("late"[All Fields] AND "onset"[All Fields] AND "alzheimer"[All Fields] AND "disease"[All Fields]) OR "late onset alzheimer disease"[All Fields]) OR ("alzheimer disease"[MeSH Terms] OR ("alzheimer"[All Fields] AND "disease"[All Fields]) OR "alzheimer disease"[All Fields] OR ("alzheimer s"[All Fields] AND "disease"[All Fields] AND "focal"[All Fields] AND "onset"[All Fields])) OR ("alzheimer disease"[MeSH Terms] OR ("alzheimer"[All Fields] AND "disease"[All Fields]) OR "alzheimer disease"[All Fields] OR ("focal"[All Fields] AND "onset"[All Fields] AND "alzheimer s"[All Fields] AND "disease"[All Fields])) OR (("alzheimer disease"[MeSH Terms] OR ("alzheimer"[All Fields] AND "disease"[All Fields]) OR "alzheimer disease"[All Fields] OR ("familial"[All Fields] AND "alzheimer"[All Fields] AND "disease"[All Fields]) OR "familial alzheimer disease"[All Fields]) AND "FAD"[All Fields]) OR ((("alzheimer disease"[MeSH Terms] OR ("alzheimer"[All Fields] AND "disease"[All Fields]) OR "alzheimer disease"[All Fields]) AND ("familialities"[All Fields] OR "familiality"[All Fields] OR "familially"[All Fields] OR "familials"[All Fields] OR "familie"[All Fields] OR "family"[MeSH Terms] OR "family"[All Fields] OR "familial"[All Fields] OR "families"[All Fields] OR "family s"[All Fields] OR "familys"[All Fields])) AND "FAD"[All Fields]) OR ((("familialities"[All Fields] OR "familiality"[All Fields] OR "familially"[All Fields] OR "familials"[All Fields] OR "familie"[All Fields] OR "family"[MeSH Terms] OR "family"[All Fields] OR "familial"[All Fields] OR "families"[All Fields] OR "family s"[All Fields] OR "familys"[All Fields]) AND ("alzheimer disease"[MeSH Terms] OR ("alzheimer"[All Fields] AND "disease"[All Fields]) OR "alzheimer disease"[All Fields] OR ("alzheimer"[All Fields] AND "diseases"[All Fields]) OR "alzheimer diseases"[All Fields])) AND "FAD"[All Fields]))) AND ("gastrodia"[MeSH Terms] OR ("gastrodia elata extract"[Supplementary Concept] OR "gastrodia elata extract"[All Fields] OR "gastrodia"[All Fields] OR "gastrodia"[MeSH Terms] OR "gastrodiae"[All Fields] OR ("gastrodia"[MeSH Terms] OR "gastrodia"[All Fields]) OR ("gastrodia elata extract"[Supplementary Concept] OR "gastrodia elata extract"[All Fields] OR "gastrodia elata"[All Fields] OR "gastrodia"[MeSH Terms] OR "gastrodia"[All Fields] OR ("gastrodia"[All Fields] AND "elata"[All Fields])) OR ("gastrodia"[MeSH Terms] OR "gastrodia"[All Fields] OR ("tien"[All Fields] AND "ma"[All Fields]) OR "tien ma"[All Fields])))

5.3 Equation 3 Gastrodia AND ischemic stroke

Records retrieved: 93

("gastrodia"[MeSH Terms] OR ("gastrodia elata extract"[Supplementary Concept] OR "gastrodia elata extract"[All Fields] OR "gastrodia"[All Fields] OR "gastrodia"[MeSH Terms] OR "gastrodiae"[All Fields] OR ("gastrodia"[MeSH Terms] OR "gastrodia"[All Fields]) OR ("gastrodia elata extract"[Supplementary Concept] OR "gastrodia elata extract"[All Fields] OR "gastrodia elata"[All Fields] OR "gastrodia"[MeSH Terms] OR "gastrodia"[All Fields] OR ("gastrodia"[All Fields] AND "elata"[All Fields])) OR ("gastrodia"[MeSH Terms] OR "gastrodia"[All Fields] OR ("tien"[All Fields] AND "ma"[All Fields]) OR "tien ma"[All Fields]))) AND ("stroke"[MeSH Terms] OR ((((("stroke"[MeSH Terms] OR "stroke"[All Fields] OR "strokes"[All Fields] OR "stroke s"[All Fields] OR ("stroke"[MeSH Terms] OR "stroke"[All Fields] OR "strokes"[All Fields] OR "stroke s"[All Fields]) OR ("stroke"[MeSH Terms] OR "stroke"[All Fields] OR ("cerebrovascular"[All Fields] AND "accident"[All Fields]) OR "cerebrovascular accident"[All Fields]) OR ("stroke"[MeSH Terms] OR "stroke"[All Fields] OR ("cerebrovascular"[All Fields] AND "accidents"[All Fields]) OR "cerebrovascular accidents"[All Fields]) OR ("stroke"[MeSH Terms] OR "stroke"[All Fields] OR ("cerebral"[All Fields] AND "stroke"[All Fields]) OR "cerebral stroke"[All Fields]) OR ("stroke"[MeSH Terms] OR "stroke"[All Fields] OR ("cerebral"[All Fields] AND "strokes"[All Fields]) OR "cerebral strokes"[All Fields]) OR ("stroke"[MeSH Terms] OR "stroke"[All Fields] OR ("stroke"[All Fields] AND "cerebral"[All Fields]) OR "stroke cerebral"[All Fields]) OR ("stroke"[MeSH Terms] OR "stroke"[All Fields] OR ("strokes"[All Fields] AND "cerebral"[All Fields]) OR "strokes cerebral"[All Fields]) OR ("stroke"[MeSH Terms] OR "stroke"[All Fields] OR ("cerebrovascular"[All Fields] AND "apoplexy"[All Fields]) OR "cerebrovascular apoplexy"[All Fields]) OR ("stroke"[MeSH Terms] OR "stroke"[All Fields] OR ("apoplexy"[All Fields] AND "cerebrovascular"[All Fields]) OR "apoplexy cerebrovascular"[All Fields]) OR ("stroke"[MeSH Terms] OR "stroke"[All Fields] OR ("vascular"[All Fields] AND "accident"[All Fields] AND "brain"[All Fields]) OR "vascular accident brain"[All Fields]) OR ("stroke"[MeSH Terms] OR "stroke"[All Fields] OR ("brain"[All Fields] AND "vascular"[All Fields] AND "accident"[All Fields]) OR "brain vascular accident"[All Fields]) OR ("stroke"[MeSH Terms] OR "stroke"[All Fields] OR ("brain"[All Fields] AND "vascular"[All Fields] AND "accidents"[All Fields]) OR "brain vascular accidents"[All Fields]) OR ("stroke"[MeSH Terms] OR "stroke"[All Fields] OR ("vascular"[All Fields] AND "accidents"[All Fields] AND "brain"[All Fields])) OR ("stroke"[MeSH Terms] OR "stroke"[All Fields] OR ("cerebrovascular"[All Fields] AND "stroke"[All Fields]) OR "cerebrovascular stroke"[All Fields]) OR ("stroke"[MeSH Terms] OR "stroke"[All Fields] OR ("cerebrovascular"[All Fields] AND "strokes"[All Fields]) OR "cerebrovascular strokes"[All Fields]) OR ("stroke"[MeSH Terms] OR "stroke"[All Fields] OR ("stroke"[All Fields] AND "cerebrovascular"[All Fields]) OR "stroke cerebrovascular"[All Fields]) OR ("stroke"[MeSH Terms] OR "stroke"[All Fields] OR ("strokes"[All Fields] AND "cerebrovascular"[All Fields]) OR "strokes cerebrovascular"[All Fields]) OR ("apoplexies"[All Fields] OR "stroke"[MeSH Terms] OR "stroke"[All Fields] OR "apoplexy"[All Fields]) OR ("stroke"[MeSH Terms] OR "stroke"[All Fields] OR "cva"[All Fields])) AND ("stroke"[MeSH Terms] OR "stroke"[All Fields] OR ("cerebrovascular"[All Fields] AND "accident"[All Fields]) OR "cerebrovascular accident"[All Fields])) OR "CVAs"[All Fields]) AND ("stroke"[MeSH Terms] OR "stroke"[All Fields] OR ("cerebrovascular"[All Fields] AND "accident"[All Fields]) OR "cerebrovascular accident"[All Fields])) OR ("stroke"[MeSH Terms] OR "stroke"[All Fields] OR ("stroke"[All Fields] AND "acute"[All Fields]) OR "stroke acute"[All Fields]) OR ("stroke"[MeSH Terms] OR "stroke"[All Fields] OR ("acute"[All Fields] AND "stroke"[All Fields]) OR "acute stroke"[All Fields]) OR ("stroke"[MeSH Terms] OR "stroke"[All Fields] OR ("acute"[All Fields] AND "strokes"[All Fields]) OR "acute strokes"[All Fields]) OR ("stroke"[MeSH Terms] OR "stroke"[All Fields] OR ("strokes"[All Fields] AND "acute"[All Fields]) OR "strokes acute"[All Fields]) OR ("stroke"[MeSH Terms] OR "stroke"[All Fields] OR ("cerebrovascular"[All Fields] AND "accident"[All Fields] AND "acute"[All Fields]) OR "cerebrovascular accident acute"[All Fields]) OR ("stroke"[MeSH Terms] OR "stroke"[All Fields] OR ("acute"[All Fields] AND "cerebrovascular"[All Fields] AND "accident"[All Fields]) OR "acute cerebrovascular accident"[All Fields]) OR ("stroke"[MeSH Terms] OR "stroke"[All Fields] OR ("acute"[All Fields] AND "cerebrovascular"[All Fields] AND "accidents"[All Fields]) OR "acute cerebrovascular accidents"[All Fields]) OR ("stroke"[MeSH Terms] OR "stroke"[All Fields] OR ("cerebrovascular"[All Fields] AND "accidents"[All Fields] AND "acute"[All Fields]) OR "cerebrovascular accidents acute"[All Fields])))

5.4 Equation 4 — Gastrodia AND Vascular Dementia

Records retrieved: 10

("gastrodia"[MeSH Terms] OR ("gastrodia elata extract"[Supplementary Concept] OR "gastrodia elata extract"[All Fields] OR "gastrodia"[All Fields] OR "gastrodia"[MeSH Terms] OR "gastrodiae"[All Fields] OR ("gastrodia"[MeSH Terms] OR "gastrodia"[All Fields]) OR ("gastrodia elata extract"[Supplementary Concept] OR "gastrodia elata extract"[All Fields] OR "gastrodia elata"[All Fields] OR "gastrodia"[MeSH Terms] OR "gastrodia"[All Fields] OR ("gastrodia"[All Fields] AND "elata"[All Fields])) OR ("gastrodia"[MeSH Terms] OR "gastrodia"[All Fields] OR ("tien"[All Fields] AND "ma"[All Fields]) OR "tien ma"[All Fields]))) AND ("dementia, vascular"[MeSH Terms] OR ("dementia, vascular"[MeSH Terms] OR ("dementia"[All Fields] AND "vascular"[All Fields]) OR "vascular dementia"[All Fields] OR "dementia vascular"[All Fields] OR ("dementia, vascular"[MeSH Terms] OR ("dementia"[All Fields] AND "vascular"[All Fields]) OR "vascular dementia"[All Fields] OR ("dementias"[All Fields] AND "vascular"[All Fields]) OR "dementias vascular"[All Fields]) OR ("dementia, vascular"[MeSH Terms] OR ("dementia"[All Fields] AND "vascular"[All Fields]) OR "vascular dementia"[All Fields] OR ("vascular"[All Fields] AND "dementias"[All Fields]) OR "vascular dementias"[All Fields]) OR ("dementia, vascular"[MeSH Terms] OR ("dementia"[All Fields] AND "vascular"[All Fields]) OR "vascular dementia"[All Fields] OR ("vascular"[All Fields] AND "dementia"[All Fields])) OR ("dementia, vascular"[MeSH Terms] OR ("dementia"[All Fields] AND "vascular"[All Fields]) OR "vascular dementia"[All Fields] OR ("arteriosclerotic"[All Fields] AND "dementia"[All Fields]) OR "arteriosclerotic dementia"[All Fields]) OR ("dementia, vascular"[MeSH Terms] OR ("dementia"[All Fields] AND "vascular"[All Fields]) OR "vascular dementia"[All Fields] OR ("arteriosclerotic"[All Fields] AND "dementias"[All Fields]) OR "arteriosclerotic dementias"[All Fields]) OR ("dementia, vascular"[MeSH Terms] OR ("dementia"[All Fields] AND "vascular"[All Fields]) OR "vascular dementia"[All Fields] OR ("dementia"[All Fields] AND "arteriosclerotic"[All Fields]) OR "dementia arteriosclerotic"[All Fields]) OR ("dementia, vascular"[MeSH Terms] OR ("dementia"[All Fields] AND "vascular"[All Fields]) OR "vascular dementia"[All Fields] OR ("dementias"[All Fields] AND "arteriosclerotic"[All Fields])) OR ("dementia, vascular"[MeSH Terms] OR ("dementia"[All Fields] AND "vascular"[All Fields]) OR "vascular dementia"[All Fields] OR ("binswanger"[All Fields] AND "disease"[All Fields]) OR "binswanger disease"[All Fields]) OR ("dementia, vascular"[MeSH Terms] OR ("dementia"[All Fields] AND "vascular"[All Fields]) OR "vascular dementia"[All Fields] OR ("disease"[All Fields] AND "binswanger"[All Fields]) OR "disease binswanger"[All Fields]) OR ("dementia, vascular"[MeSH Terms] OR ("dementia"[All Fields] AND "vascular"[All Fields]) OR "vascular dementia"[All Fields] OR ("encephalopathy"[All Fields] AND "binswanger"[All Fields]) OR "encephalopathy binswanger"[All Fields]) OR ("dementia, vascular"[MeSH Terms] OR ("dementia"[All Fields] AND "vascular"[All Fields]) OR "vascular dementia"[All Fields] OR ("chronic"[All Fields] AND "progressive"[All Fields] AND "subcortical"[All Fields] AND "encephalopathy"[All Fields]) OR "chronic progressive subcortical encephalopathy"[All Fields]) OR ("dementia, vascular"[MeSH Terms] OR ("dementia"[All Fields] AND "vascular"[All Fields]) OR "vascular dementia"[All Fields] OR ("binswanger"[All Fields] AND "encephalopathy"[All Fields]) OR "binswanger encephalopathy"[All Fields]) OR ("dementia, vascular"[MeSH Terms] OR ("dementia"[All Fields] AND "vascular"[All Fields]) OR "vascular dementia"[All Fields] OR ("leukoencephalopathy"[All Fields] AND "subcortical"[All Fields]) OR "leukoencephalopathy subcortical"[All Fields]) OR ("dementia, vascular"[MeSH Terms] OR ("dementia"[All Fields] AND "vascular"[All Fields]) OR "vascular dementia"[All Fields] OR ("leukoencephalopathies"[All Fields] AND "subcortical"[All Fields])) OR ("dementia, vascular"[MeSH Terms] OR ("dementia"[All Fields] AND "vascular"[All Fields]) OR "vascular dementia"[All Fields] OR ("subcortical"[All Fields] AND "leukoencephalopathies"[All Fields])) OR ("dementia, vascular"[MeSH Terms] OR ("dementia"[All Fields] AND "vascular"[All Fields]) OR "vascular dementia"[All Fields] OR ("encephalopathy"[All Fields] AND "subcortical"[All Fields] AND "arteriosclerotic"[All Fields]) OR "encephalopathy subcortical arteriosclerotic"[All Fields]) OR ("dementia, vascular"[MeSH Terms] OR ("dementia"[All Fields] AND "vascular"[All Fields]) OR "vascular dementia"[All Fields] OR ("encephalopathy"[All Fields] AND "chronic"[All Fields] AND "progressive"[All Fields] AND "subcortical"[All Fields])) OR ("dementia, vascular"[MeSH Terms] OR ("dementia"[All Fields] AND "vascular"[All Fields]) OR "vascular dementia"[All Fields] OR ("encephalopathy"[All Fields] AND "subcortical"[All Fields] AND "chronic"[All Fields] AND "progressive"[All Fields])) OR ("dementia, vascular"[MeSH Terms] OR ("dementia"[All Fields] AND "vascular"[All Fields]) OR "vascular dementia"[All Fields] OR ("subcortical"[All Fields] AND "encephalopathy"[All Fields] AND "chronic"[All Fields] AND "progressive"[All Fields])) OR ("dementia, vascular"[MeSH Terms] OR ("dementia"[All Fields] AND "vascular"[All Fields]) OR "vascular dementia"[All Fields] OR ("subcortical"[All Fields] AND "leukoencephalopathy"[All Fields]) OR "subcortical leukoencephalopathy"[All Fields]) OR ("dementia, vascular"[MeSH Terms] OR ("dementia"[All Fields] AND "vascular"[All Fields]) OR "vascular dementia"[All Fields] OR ("subcortical"[All Fields] AND "arteriosclerotic"[All Fields] AND "encephalopathy"[All Fields]) OR "subcortical arteriosclerotic encephalopathy"[All Fields]) OR ("dementia, vascular"[MeSH Terms] OR ("dementia"[All Fields] AND "vascular"[All Fields]) OR "vascular dementia"[All Fields] OR ("arteriosclerotic"[All Fields] AND "encephalopathy"[All Fields] AND "subcortical"[All Fields])) OR ("dementia, vascular"[MeSH Terms] OR ("dementia"[All Fields] AND "vascular"[All Fields]) OR "vascular dementia"[All Fields] OR ("arteriosclerotic"[All Fields] AND "encephalopathies"[All Fields] AND "subcortical"[All Fields])) OR ("dementia, vascular"[MeSH Terms] OR ("dementia"[All Fields] AND "vascular"[All Fields]) OR "vascular dementia"[All Fields] OR ("encephalopathies"[All Fields] AND "subcortical"[All Fields] AND "arteriosclerotic"[All Fields])) OR ("dementia, vascular"[MeSH Terms] OR ("dementia"[All Fields] AND "vascular"[All Fields]) OR "vascular dementia"[All Fields] OR ("subcortical"[All Fields] AND "arteriosclerotic"[All Fields] AND "encephalopathies"[All Fields])) OR ("dementia, vascular"[MeSH Terms] OR ("dementia"[All Fields] AND "vascular"[All Fields]) OR "vascular dementia"[All Fields] OR ("encephalopathy"[All Fields] AND "binswanger s"[All Fields]) OR "encephalopathy binswanger s"[All Fields]) OR ("dementia, vascular"[MeSH Terms] OR ("dementia"[All Fields] AND "vascular"[All Fields]) OR "vascular dementia"[All Fields] OR ("binswanger s"[All Fields] AND "encephalopathy"[All Fields]) OR "binswanger s encephalopathy"[All Fields]) OR ("dementia, vascular"[MeSH Terms] OR ("dementia"[All Fields] AND "vascular"[All Fields]) OR "vascular dementia"[All Fields] OR ("encephalopathy"[All Fields] AND "binswangers"[All Fields]) OR "encephalopathy binswangers"[All Fields]) OR ("dementia, vascular"[MeSH Terms] OR ("dementia"[All Fields] AND "vascular"[All Fields]) OR "vascular dementia"[All Fields] OR ("binswanger s"[All Fields] AND "disease"[All Fields]) OR "binswanger s disease"[All Fields]) OR ("dementia, vascular"[MeSH Terms] OR ("dementia"[All Fields] AND "vascular"[All Fields]) OR "vascular dementia"[All Fields] OR ("binswangers"[All Fields] AND "disease"[All Fields]) OR "binswangers disease"[All Fields]) OR ("dementia, vascular"[MeSH Terms] OR ("dementia"[All Fields] AND "vascular"[All Fields]) OR "vascular dementia"[All Fields] OR ("disease"[All Fields] AND "binswanger s"[All Fields]) OR "disease binswanger s"[All Fields]) OR ("dementia, vascular"[MeSH Terms] OR ("dementia"[All Fields] AND "vascular"[All Fields]) OR "vascular dementia"[All Fields] OR ("subcortical"[All Fields] AND "vascular"[All Fields] AND "dementia"[All Fields]) OR "subcortical vascular dementia"[All Fields]) OR ("dementia, vascular"[MeSH Terms] OR ("dementia"[All Fields] AND "vascular"[All Fields]) OR "vascular dementia"[All Fields] OR ("dementias"[All Fields] AND "subcortical"[All Fields] AND "vascular"[All Fields])) OR ("dementia, vascular"[MeSH Terms] OR ("dementia"[All Fields] AND "vascular"[All Fields]) OR "vascular dementia"[All Fields] OR ("dementia"[All Fields] AND "subcortical"[All Fields] AND "vascular"[All Fields]) OR "dementia subcortical vascular"[All Fields]) OR ("dementia, vascular"[MeSH Terms] OR ("dementia"[All Fields] AND "vascular"[All Fields]) OR "vascular dementia"[All Fields] OR ("subcortical"[All Fields] AND "vascular"[All Fields] AND "dementias"[All Fields]) OR "subcortical vascular dementias"[All Fields]) OR ("dementia, vascular"[MeSH Terms] OR ("dementia"[All Fields] AND "vascular"[All Fields]) OR "vascular dementia"[All Fields] OR ("vascular"[All Fields] AND "dementias"[All Fields] AND "subcortical"[All Fields]) OR "vascular dementias subcortical"[All Fields]) OR ("dementia, vascular"[MeSH Terms] OR ("dementia"[All Fields] AND "vascular"[All Fields]) OR "vascular dementia"[All Fields] OR ("vascular"[All Fields] AND "dementia"[All Fields] AND "subcortical"[All Fields]) OR "vascular dementia subcortical"[All Fields]) OR ("dementia, vascular"[MeSH Terms] OR ("dementia"[All Fields] AND "vascular"[All Fields]) OR "vascular dementia"[All Fields] OR ("vascular"[All Fields] AND "dementia"[All Fields] AND "acute"[All Fields] AND "onset"[All Fields])) OR ("dementia, vascular"[MeSH Terms] OR ("dementia"[All Fields] AND "vascular"[All Fields]) OR "vascular dementia"[All Fields] OR ("acute"[All Fields] AND "onset"[All Fields] AND "vascular"[All Fields] AND "dementia"[All Fields]) OR "acute onset vascular dementia"[All Fields])))

5.5 Equation 5 — Gastrodia AND Epilepsy

Records retrieved: 81

("gastrodia"[MeSH Terms] OR ("gastrodia elata extract"[Supplementary Concept] OR "gastrodia elata extract"[All Fields] OR "gastrodia"[All Fields] OR "gastrodia"[MeSH Terms] OR "gastrodiae"[All Fields] OR ("gastrodia"[MeSH Terms] OR "gastrodia"[All Fields]) OR ("gastrodia elata extract"[Supplementary Concept] OR "gastrodia elata extract"[All Fields] OR "gastrodia elata"[All Fields] OR "gastrodia"[MeSH Terms] OR "gastrodia"[All Fields] OR ("gastrodia"[All Fields] AND "elata"[All Fields])) OR ("gastrodia"[MeSH Terms] OR "gastrodia"[All Fields] OR ("tien"[All Fields] AND "ma"[All Fields]) OR "tien ma"[All Fields]))) AND ("epilepsy"[MeSH Terms] OR ("epilepsie"[All Fields] OR "epilepsy"[MeSH Terms] OR "epilepsy"[All Fields] OR "epilepsies"[All Fields] OR "epilepsy s"[All Fields] OR ("epilepsie"[All Fields] OR "epilepsy"[MeSH Terms] OR "epilepsy"[All Fields] OR "epilepsies"[All Fields] OR "epilepsy s"[All Fields]) OR ("epilepsy"[MeSH Terms] OR "epilepsy"[All Fields] OR ("seizure"[All Fields] AND "disorder"[All Fields]) OR "seizure disorder"[All Fields]) OR ("epilepsy"[MeSH Terms] OR "epilepsy"[All Fields] OR ("seizure"[All Fields] AND "disorders"[All Fields]) OR "seizure disorders"[All Fields]) OR ("epilepsy"[MeSH Terms] OR "epilepsy"[All Fields] OR ("epilepsy"[All Fields] AND "cryptogenic"[All Fields]) OR "epilepsy cryptogenic"[All Fields]) OR ("epilepsy"[MeSH Terms] OR "epilepsy"[All Fields] OR ("cryptogenic"[All Fields] AND "epilepsies"[All Fields]) OR "cryptogenic epilepsies"[All Fields]) OR ("epilepsy"[MeSH Terms] OR "epilepsy"[All Fields] OR ("cryptogenic"[All Fields] AND "epilepsy"[All Fields]) OR "cryptogenic epilepsy"[All Fields]) OR ("epilepsy"[MeSH Terms] OR "epilepsy"[All Fields] OR ("epilepsies"[All Fields] AND "cryptogenic"[All Fields]) OR "epilepsies cryptogenic"[All Fields]) OR ("epilepsy"[MeSH Terms] OR "epilepsy"[All Fields] OR "aura"[All Fields]) OR ("epilepsy"[MeSH Terms] OR "epilepsy"[All Fields] OR "auras"[All Fields]) OR ("epilepsy"[MeSH Terms] OR "epilepsy"[All Fields] OR ("awakening"[All Fields] AND "epilepsy"[All Fields]) OR "awakening epilepsy"[All Fields]) OR "epilepsy"[MeSH Terms]))

6. Cross-disease combination

The five disease-specific sets were combined with Boolean OR and de-duplicated in EndNote X9. The equivalent PubMed Advanced Search Builder query is:

((((((Parkinson Disease[MeSH Terms]) OR (Idiopathic Parkinson Disease OR Idiopathic Parkinson's Disease OR Lewy Body Parkinson Disease OR Lewy Body Parkinson's Disease OR Paralysis Agitans OR Parkinson Disease, Idiopathic OR Parkinson's Disease OR Parkinson's Disease, Idiopathic OR Parkinson's Disease, Lewy Body OR Primary Parkinsonism OR Parkinsonism, Primary)) OR ((Alzheimer Disease[MeSH Terms]) OR (Alzheimer Syndrome OR (Alzheimer-Type Dementia (ATD)) OR (Alzheimer Type Dementia (ATD)) OR (Dementia, Alzheimer-Type (ATD)) OR Alzheimer's Diseases OR Alzheimer Diseases OR Alzheimers Diseases OR Alzheimer Dementia OR Alzheimer Dementias OR Dementia, Alzheimer OR Alzheimer's Disease OR Dementia, Senile OR Senile Dementia OR Dementia, Alzheimer Type OR Alzheimer Type Dementia OR Senile Dementia, Alzheimer Type OR Alzheimer Type Senile Dementia OR Primary Senile Degenerative Dementia OR Alzheimer Sclerosis OR Sclerosis, Alzheimer OR Dementia, Primary Senile Degenerative OR Dementia, Presenile OR Presenile Dementia OR Acute Confusional Senile Dementia OR Senile Dementia, Acute Confusional OR Alzheimer Disease, Early Onset OR Early Onset Alzheimer Disease OR Presenile Alzheimer Dementia OR Alzheimer Disease, Late Onset OR Late Onset Alzheimer Disease OR Alzheimer's Disease, Focal Onset OR Focal Onset Alzheimer's Disease OR (Familial Alzheimer Disease (FAD)) OR (Alzheimer Disease, Familial (FAD)) OR (Familial Alzheimer Diseases (FAD))))) OR ((Stroke[MeSH Terms]) OR (Stroke OR Strokes OR Cerebrovascular Accident OR Cerebrovascular Accidents OR Cerebral Stroke OR Cerebral Strokes OR Stroke, Cerebral OR Strokes, Cerebral OR Cerebrovascular Apoplexy OR Apoplexy, Cerebrovascular OR Vascular Accident, Brain OR Brain Vascular Accident OR Brain Vascular Accidents OR Vascular Accidents, Brain OR Cerebrovascular Stroke OR Cerebrovascular Strokes OR Stroke, Cerebrovascular OR Strokes, Cerebrovascular OR Apoplexy OR CVA (Cerebrovascular Accident) OR CVAs (Cerebrovascular Accident) OR Stroke, Acute OR Acute Stroke OR Acute Strokes OR Strokes, Acute OR Cerebrovascular Accident, Acute OR Acute Cerebrovascular Accident OR Acute Cerebrovascular Accidents OR Cerebrovascular Accidents, Acute))) OR ((Dementia, Vascular[MeSH Terms]) OR (Dementia, Vascular OR Dementias, Vascular OR Vascular Dementias OR Vascular Dementia OR Arteriosclerotic Dementia OR Arteriosclerotic Dementias OR Dementia, Arteriosclerotic OR Dementias, Arteriosclerotic OR Binswanger Disease OR Disease, Binswanger OR Encephalopathy, Binswanger OR Chronic Progressive Subcortical Encephalopathy OR Binswanger Encephalopathy OR Leukoencephalopathy, Subcortical OR Leukoencephalopathies, Subcortical OR Subcortical Leukoencephalopathies OR Encephalopathy, Subcortical Arteriosclerotic OR Encephalopathy, Chronic Progressive Subcortical OR Encephalopathy, Subcortical, Chronic Progressive OR Subcortical Encephalopathy, Chronic Progressive OR Subcortical Leukoencephalopathy OR Subcortical Arteriosclerotic Encephalopathy OR Arteriosclerotic Encephalopathy, Subcortical OR Arteriosclerotic Encephalopathies, Subcortical OR Encephalopathies, Subcortical Arteriosclerotic OR Subcortical Arteriosclerotic Encephalopathies OR Encephalopathy, Binswanger's OR Binswanger's Encephalopathy OR Encephalopathy, Binswangers OR Binswanger's Disease OR Binswangers Disease OR Disease, Binswanger's OR Subcortical Vascular Dementia OR Dementias, Subcortical Vascular OR Dementia, Subcortical Vascular OR Subcortical Vascular Dementias OR Vascular Dementias, Subcortical OR Vascular Dementia, Subcortical OR Vascular Dementia, Acute Onset OR Acute Onset Vascular Dementia))) OR ((Epilepsy[MeSH Terms]) OR (Epilepsy OR Epilepsies OR Seizure Disorder OR Seizure Disorders OR Epilepsy, Cryptogenic OR Cryptogenic Epilepsies OR Cryptogenic Epilepsy OR Epilepsies, Cryptogenic OR Aura OR Auras OR Awakening Epilepsy OR Epilepsy, Awakening[MeSH Terms]))) AND ((Gastrodia[MeSH Terms]) OR (Gastrodia OR Gastrodias OR Gastrodia elata OR Tien ma))

7. Filters and limits

Date range: none (records from database inception through 31 May 2026).

Language: none at search stage; English and Chinese articles were eligible; non-English/non-Chinese records were excluded only during eligibility screening.

Publication type: none at search stage; reviews, meta-analyses, commentaries, editorials, conference abstracts and case reports were excluded during screening.

Species: none at search stage; animal, in vitro and human studies were all eligible for retrieval.

Document type: journal articles indexed in PubMed; indexed preprints were eligible.

8. Search results (final search: 31 May 2026)

8.1 Gastrodia AND Parkinson Disease Records retrieved: 46

8.2 Gastrodia AND Alzheimer Disease Records retrieved: 65

8.3 Gastrodia AND ischemic stroke Records retrieved: 93

8.4 Gastrodia AND Vascular Dementia Records retrieved: 10

8.5 Gastrodia AND Epilepsy Records retrieved: 81

8.6 Cross-disease combination: 244

9. Reproducibility

The five strings in Section 5 can be copy-pasted directly into the PubMed Advanced Search Builder (https://pubmed.ncbi.nlm.nih.gov/advanced/) to reproduce the result counts reported in Section 8. MeSH terms are auto-exploded by PubMed and should not be disabled. Wildcard truncation was avoided; full word forms were used to prevent unintended matches.
